# Screening of a Small Molecule Compound Library Identifies Toosendanin as an Inhibitor Against Bunyavirus and SARS-CoV-2

**DOI:** 10.3389/fphar.2021.735223

**Published:** 2021-11-11

**Authors:** Shufen Li, Meidi Ye, Yuanqiao Chen, Yulan Zhang, Jiachen Li, Wei Liu, Hao Li, Ke Peng

**Affiliations:** ^1^ State Key Laboratory of Virology, Center for Biosafety Mega-Science, Wuhan Institute of Virology, Chinese Academy of Sciences, Wuhan, China; ^2^ Savaid Medical School, University of Chinese Academy of Sciences, Beijing, China; ^3^ State Key Laboratory of Pathogen and Biosecurity, Beijing Institute of Microbiology and Epidemiology, Beijing, China

**Keywords:** bunyavirus, SFTSV, SARS-CoV-2, anti-viral drug screening, toosendanin

## Abstract

Severe fever with thrombocytopenia syndrome virus (SFTSV) is an emerging tick-borne virus causing serious infectious disease with a high case-fatality of up to 50% in severe cases. Currently, no effective drug has been approved for the treatment of SFTSV infection. Here, we performed a high-throughput screening of a natural extracts library for compounds with activities against SFTSV infection. Three hit compounds, notoginsenoside Ft1, punicalin, and toosendanin were identified for displaying high anti-SFTSV efficacy, in which, toosendanin showed the highest inhibition potency. Mechanistic investigation indicated that toosendanin inhibited SFTSV infection at the step of virus internalization. The anti-viral effect of toosendanin against SFTSV was further verified in mouse infection models, and the treatment with toosendanin significantly reduced viral load and histopathological changes *in vivo*. The antiviral activity of toosendanin was further expanded to another bunyavirus and the emerging SARS-CoV-2. This study revealed a broad anti-viral effect of toosendanin and indicated its potential to be developed as an anti-viral drug for clinical use.

## Introduction

Severe fever with thrombocytopenia syndrome (SFTS) is an emerging infectious disease caused by a novel tick-borne virus (*Dabie bandavirus*, formerly *SFTS virus* and *Huaiyangshan banyangvirus*), which belongs to the genus *Bandavirus*, family *Phenuiviridae*, order *Bunyavirales* (Yoshikawa et al., 2021). Genome of the virus consists of three negative-stranded RNA segments, encoding four proteins, including RNA-dependent RNA polymerase (RdRp), glycoprotein precursor (GPC), nucleoprotein (N), and nonstructural protein (NSs). The clinical symptoms of SFTS are high fever, diarrhea, vomiting, thrombocytopenia, leukocytopenia, encephalitis, hemorrhage, and multiple organ failure with high case-fatality rate (approximately 12–50%) (Xu et al., 2011; Robles et al., 2018). SFTS was first identified in China and then in South Korea, Japan, and Vietnam (Xu et al., 2011; Yu et al., 2011; Kim et al., 2013; Takahashi et al., 2014; Tran et al., 2019; Peng et al., 2020). The spread of SFTSV is mainly through tick-bites with *Haemaphysalis longicornis* tick as the vector, which has been found in East Asia, Pacific, Oceania region, and North America (Hammer et al., 2015; Luo et al., 2015; Heath, 2016; Yun et al., 2017; Hutcheson et al., 2019). Person-to-person transmission and aerosol transmission through close contact or exposure to contaminated environment have also been reported (Bao et al., 2011; Moon et al., 2018; Jung et al., 2019). SFTSV has become a global health concern due to its high case-fatality and pandemic transmission risk.

Several compounds have been identified to have anti-SFTSV activities *in vitro* or *in vivo*, including ribavirin, favipiravir (T-705), and hexachlorophene (Liu et al., 2013; Tani et al., 2016; Gowen et al., 2017; Lee et al., 2017; Tani et al., 2018; Yuan et al., 2019). Our recent retrospective clinical study revealed that treatment of SFTS patient with calcium channel blocker (nifedipine) could facilitate virus clearance, clinical recovery, and reduction of the case-fatality rate (Li et al., 2019a). However, so far, no vaccines or specific drugs against SFTSV have been licensed or approved for use in the clinical treatment of SFTS. Hence the development of effective therapeutics for SFTS is in urgent need.

In order to identify more candidates with clinical application potential, a high-throughput screen assay was conducted with a natural extracts library. Further experiments were performed to identify the viral replication events that are inhibited by the compound. The inhibitory effect was also evaluated on SFTSV infected mouse model. Of this library, Toosendanin was identified to have anti-SFTSV effect both *in vitro* and *in vivo*, which also strongly inhibited the replication of another bunyavirus, Rift Valley fever virus, and the SARS-CoV-2. This study revealed the broad anti-viral effect of toosendanin and suggested its potential to be developed as an anti-viral drug for clinical use.

## Methods

### Cell Lines

Vero and HUVEC cells were obtained from American Type Culture Collection (ATCC), Huh7 and BSR-T7 cells were obtained from China Center for Type Culture Collection. All cells were cultured in Dulbecco’s modified Eagle’s medium (DMEM; Gibco) supplemented with 10% fetal bovine serum (Gibco) and 1% antibiotics (Gibco) in a humidified incubator of 5% CO_2_ at 37°C. The expression of T7 polymerase in BSR-T7 cells were selected by the addition of 1 mg/ml G418 (Thermo Fisher Scientific).

### Viruses

The SFTSV isolate HBMC16 (GenBank accession number: KY440775.1, KY440776.1 and KY440777.1) were obtained from China Centre for General Virus Culture Collection and was propagated in Vero cells. Viral titer was determined by focus-forming assay on Vero cell as described previously (Li et al., 2020). The SARS-CoV-2 (IVCAS 6.7512) was propagated and titered in Vero E6 cells (Zhou et al., 2020). Rift Valley fever virus (RVFV) was generated by using the infectious clones of RVFV BJ01 based on a T7 polymerase dependent system as described previously (Li et al., 2019b). All infection experiments with SARS-CoV-2 and RVFV were performed in a biosafety level 3 (BSL-3) facilities.

### High-Throughput Screening of Small Molecular Compound Library

A small molecular compound library of 1,058 compounds derived from natural products, including Melia Toosendanin Sieb. et Zucc and pomegranate rind, was purchased from Weikeqi Biotech (Si Chuan, China). The compounds were purified with HPLC, the purity of compounds was ≥98%. Vero cells pre-seeded in 96-well plates at a density of 10,000 cells per well were treated with each compound at a final concentration of 10 μM for 1 h and infected with SFTSV at an MOI of 0.125. Vehicle (DMSO) was used as a negative control and benidipine (10 μM) was used as a positive control based on our previous study (Li et al., 2019a). The percentage of DMSO in vehicle group was 1‰, same as the percentage of DMSO in compound group. At 36 h post infection (p.i.), cells were fixed with 4% paraformaldehyde (PFA), followed by permeabilization with 0.2% (v/v) Triton X-100 and blocking with 3% (w/v) bovine serum albumin (BSA). Then cells were incubated with rabbit anti-NP antibody, followed by anti-rabbit Alexa488 (ThermoFisher Scientific) and DAPI (Beyotime). The images were captured by an Operetta high-content imaging system (PerkinElmer) and analyzed by Harmony 3.5 software (Li et al., 2019a). The infection rate of DMSO control was set as 100%, and the infection rate of each drug was calculated by normalizing to DMSO control. The percentage inhibition was equal to 100% minus infection rate in the drug treated samples. The survival rate of DMSO control was set as 100%, and the survival rate of each drug was calculated by normalizing to DMSO control.

### Cell Viability Assay

Cells pre-seeded in 96-well plates were treated with different concentrations of drugs for 36 h. Cell viability was measured by Cell Counting kit-8 (CCK-8; Dojindo Molecular Technologies) according to the manufacturer’s instructions.

### RNA Isolation and Quantitative Reverse Transcription PCR

Total RNAs in infected cells were extracted with FastPure Cell/Tissue Total RNA Isolation Kit (Vazyme, China) following the manufacturer’s instructions. Quantitative reverse transcription PCR (qRT-PCR) was carried out with a two-step procedure. First, the cDNA of viral genome was generated by using the HiScript III 1st Strand cDNA Synthesis Kit (Vazyme, China), and then quantified by using ChamQ Universal SYBR qPCR Master Mix (Vazyme, China). Primer sequences are provided in Supplementary Table S1.

### Western Blot Analysis

Infected cells lysates were subjected to 12% SDS-polyacrylamide gel electrophoresis (PAGE), and then transferred to polyvinylidene difluoride (PVDF) membranes (Millipore). The proteins were immunoblotted with antibodies against viral nucleoproteins (Li et al., 2019a), Tubulin (Polyclonal rabbit, Proteintech, 11224-1-AP) or GAPDH (Monoclonal mouse, Proteintech, 60004-1-Ig) then HRP-conjugated goat anti-Rabbit IgG (Proteintech, SA00001-2) and anti-Mouse IgG (Proteintech, SA00001-1). Prestained protein ladder was purchased from ThermoFisher (26616). Bands were detected by a Chemiluminescence Analyzer (Chemiscope600pro) using an enhanced chemiluminescence (ECL) kit (Millipore).

### Antiviral Activity of Three Hits on Different Cell Lines

Vero, Huh-7 and HUVEC cells pre-treated with different concentrations of notoginsenoside Ft1, punicalin, and toosendanin were infected with SFTSV for 24 h. The viral RNA in the supernatant and infected cells was quantified with qRT-PCR. Infectious virus in supernatant was determined by focus-forming assay. The immunofluorescence assay was performed and analyzed as described above.

### Time-of-Addition Assay

For the determination of inactivation effect of punicalin and toosendanin. SFTSV was incubated with punicalin and toosendanin at 37°C for 1 h, and then diluted 10,000-fold for titer determination. Time-of-addition assay was performed as described previously with adjustment (Wang et al., 2017a). HUVEC cells were infected with SFTSV or RVFV at an MOI of 2 (0 h), the initial inoculum was removed at 2 h post infection. For the pre group, cells were pre-treated with toosendanin (1 μM) for 1 h, and incubated with virus for 2 h, and then drug and virus were removed. For the post group, cells were infected with SFTSV for 2 h, and toosendanin was added after the removal of virus. For the whole group, cells were treated with toosendanin from 1 h pre-infection to 12 h post infection. Infected cells were collected at 12 h post infection and subjected to qRT-PCR and western blot. Vero-E6 cells were infected with SARS-CoV-2 at an MOI of 0.01 for 1 h, and treated with toosendanin (1 μM) as described above. Infected cells were collected at 24 h post infection for qRT-PCR and western blot.

### Binding Assay

HUVEC cells were pretreated with different concentrations of toosendanin or vehicle (DMSO) at 37°C for 1 h. Then the cells were chilled at 4°C for 15 min, and then incubated with SFTSV (MOI = 5) at 4°C for 2 h. The unbound virions were removed by wash with pre-cooled PBS, cells were harvested and lysed and the relative level of bound virions was quantified by qRT-PCR.

### Internalization Assay

HUVEC cells pre-treated with different concentrations of toosendanin or vehicle (DMSO) for 1 h were chilled at 4°C for 15 min and incubated with SFTSV (MOI = 5) for 1 h. Cells were washed with pre-cooled PBS to eliminate the unbound virions and incubated at 37°C for 3 h in the presence of ammonium chloride (20 mM), then washed with PBS and treated with trypsin to remove the un-internalized virions. The relative level of internalized virions was determined by qRT-PCR.

For the visualizing assay, HUVEC cells pre-treated with toosendanin (2 μM) or vehicle (DMSO) were infected with SFTSV (MOI = 100) for 2 h in the presence of ammonium chloride (20 mM). Cells were washed with PBS and treated with trypsin, then fixed and subjected to immunofluorescence assay. Images were capture by a confocal microscope (Andor Dragonfly 202), and internalized viral particles were analyzed and quantitated by Imaris software (Imaris V9.2.1, Bitplane Inc.).

### Syncytium Formation Assay

HUVEC cells were infected with SFTSV (MOI = 5) for 24 h and treated with indicated concentrations of toosendanin or vehicle (DMSO) for 1 h, followed by incubation of citric acid-sodium citrate buffer (0.1 M citric acid, 0.1 M sodium citrate, pH 5.0) for 20 min. Cells were fixed by methanol and stained by Giemsa solution (Sigma). Syncytium formation was imaged by an epifluorescence microscope (Olympus IX73).

### Mini-Genome Assay

T7 mini-genome-eGFP plasmid and viral proteins (L and NP) expression plasmids were co-transfected to BSR-T7 cells using lipofectamine 3000 (Thermo Fisher Scientific). Different concentrations of toosendanin or vehicle (DMSO) were added 6 h post transfection, and benidipine (20 μM) was used as a positive control. The eGFP signal was captured by an epifluorescence microscope (Olympus IX73) at 24 h post transfection and analyzed with Fiji.

### Animal Study

Animal experiment was approved by the Animal Care Committee of the Wuhan Institute of Virology (permit number: WIVA38202007) and was performed in accordance with the ethical guidelines.

#### C57BL/6 Mouse Model

Eight-week-old female C57BL/6 mice purchased from Charles River Laboratories (Beijing, China) were divided into three groups (5 mice per group): Mock-treated group, vehicle (0.25% DMSO) group, and toosendanin group. Toosendanin or the same volume of vehicle was given to mice by intraperitoneal injection with the dose of 1 mg/kg/d 3 days prior to the infection. Mice were intraperitoneally inoculated with 10^5^ FFU of SFTSV and the administration of toosendanin continued for 3 days. The C57BL/6 mice were monitored daily and sacrificed on 3 days post infection. Spleen samples were collected for viral load determination, hematoxylin-eosin staining and immunohistochemistry.

#### Anti-IFNAR1 IgG Treated C57BL/6 Mouse Model

Five-week-old female C57BL/6 mice purchased from Charles River Laboratories (Beijing, China) were divided into two groups (7 mice per group): vehicle (1% DMSO) group and toosendanin group and treated with anti-IFNAR1 IgG 1 day prior to the infection (Li et al., 2020). Mice were intraperitoneally infected with 2000 FFU of SFTSV. Toosendanin or the same volume of vehicle was given to mice by intraperitoneal injection with the dose of 4 mg/kg/d 1 h post infection. The mice were monitored daily and sacrificed on 4 days post infection. Spleen samples were collected for immunohistochemistry and serum samples were harvested for viral load determination.

### Statistical Analyses

All statistical analyses were performed in GraphPad Prism version 8.4.3, as defined in the text and figure legends.

## Results

### High-Throughput Screening for Inhibitors of SFTSV Infection From a Small Molecule Compound Library

**FIGURE 1:**
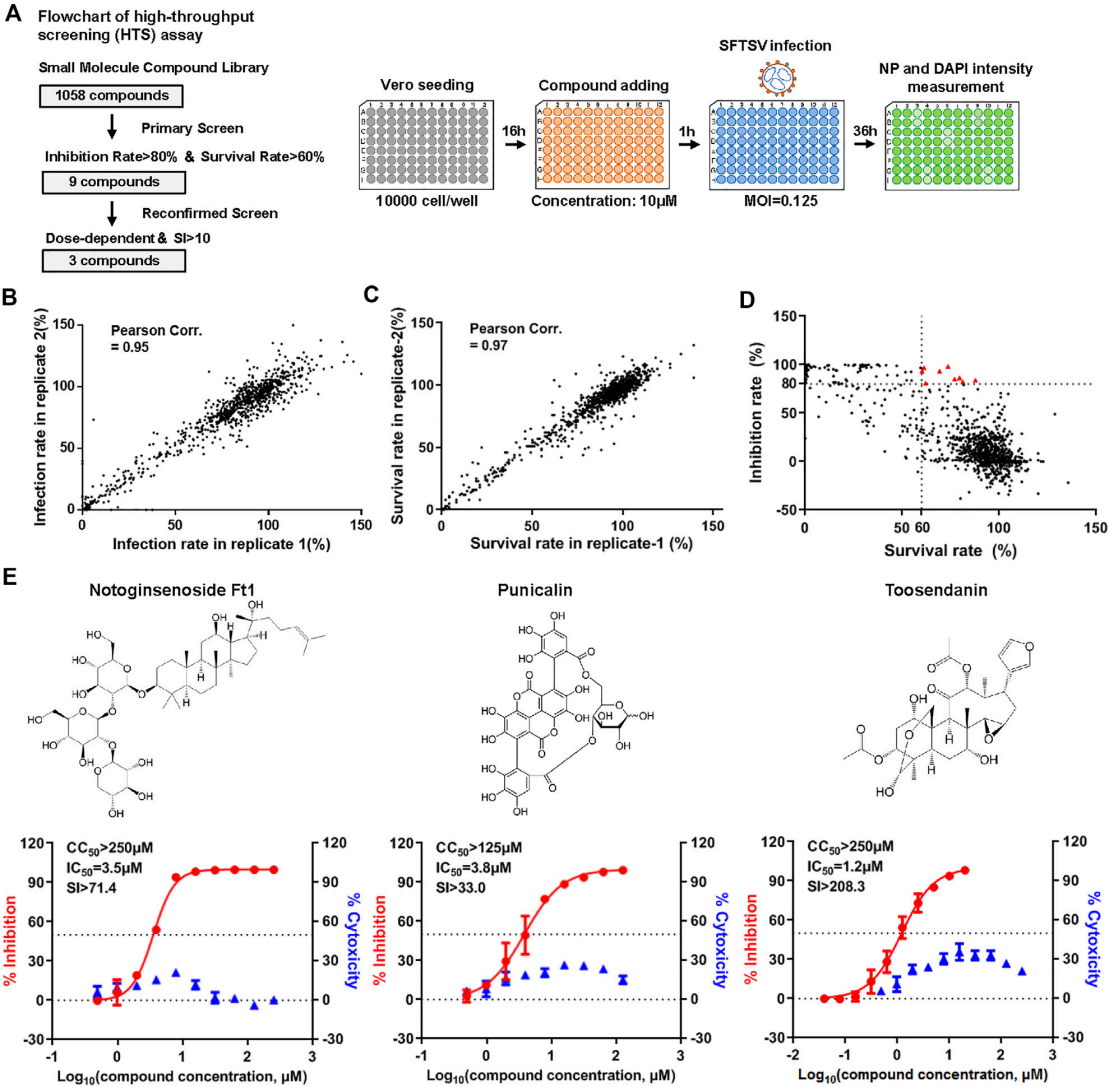
High-throughput screening (HTS) of SFTSV inhibitors from a natural extracts library. **(A)** Flowchart of HTS. **(B)** The correlation of infection rate between two replicates. **(C)** The correlation of survival rate between two replicates. **(D)** Primary screen identified nine candidates with inhibition rate >80% and survival rate is >60% (red triangle). **(E)** Reconfirmed screen identified three compounds with dose-dependent inhibitory effect against SFTSV infection. The left and right *Y*-axis represent inhibition and cytotoxicity rate of the drugs, respectively.

In order to identify potent inhibitors against SFTSV infection, we performed a high-throughput screening (HTS) of a small molecular compound library consisting of 1,058 natural extracts (Figure 1A). Briefly, Vero cells pre-treated with the compounds (10 μM) using vehicle (DMSO) as a negative control and benidipine as a positive control were infected with SFTSV for 36 h. The infection rate was determined with the immunofluorescence analysis (IFA) assay using antibody against the viral nucleoprotein (NP) (Li et al., 2019a). The correlation between two replicates was evaluated by Pearson correlation coefficient analysis (Figures 1B,C). Nine compounds were identified as candidates with inhibition rate >80% and the treated cell survival rate >60% (Figure 1D). Another screen was carried out to identify compounds with anti-viral effect in a dose-dependent manner. Three compounds, notoginsenoside Ft1, punicalin, and toosendanin, were identified as hits showing an anti-viral effect in a dose-dependent manner and with the selective index >10 (SI, 50% cytotoxic concentration [CC_50_]/50% inhibitory concentration [IC_50_]) (Figure 1E).

### Validation of the Anti-Viral Activity of the Hit Compounds

**FIGURE 2:**
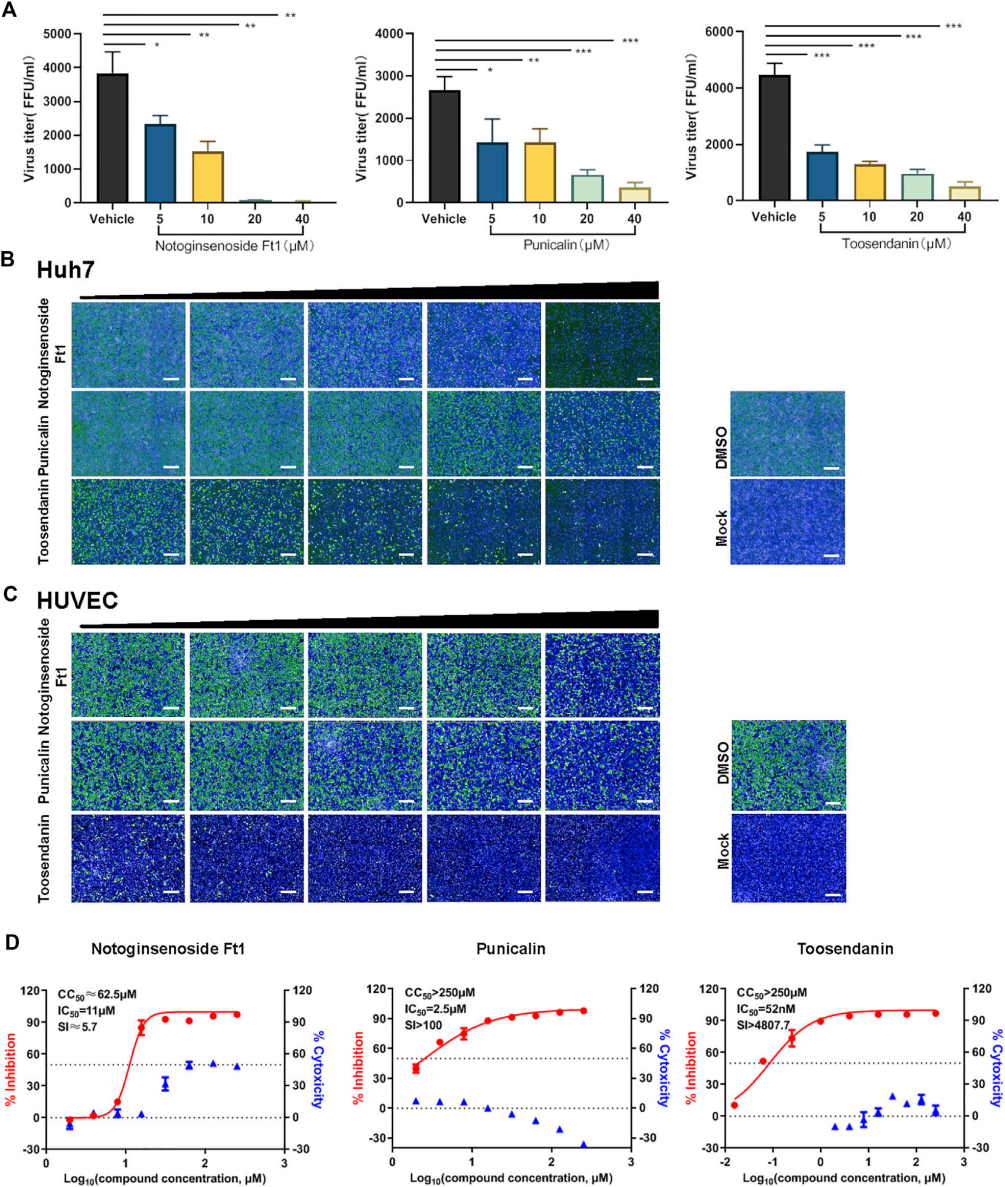
Validation of the anti-viral activity of the hit compounds in different cell lines. **(A)** Vero cells pre-treated with different concentrations of drugs were infected with SFTSV at an MOI of 0.125. Cell culture supernatants were harvested for viral titration assay at 24 hpi. **(B,C)** The anti-SFTSV activity of three compounds on Huh7 **(B)** and HUVEC **(C)** cells. Cells were pre-treated with different concentrations of drugs and infected with SFTSV at an MOI of 1. Cells were fixed for immunofluorescence assay at 36 hpi. Bars represent 500 μm. **(D)** The dose-dependent inhibitory activity of three compounds against SFTSV infection on HUVEC cells. The left and right *Y*-axis represent inhibition and cytotoxicity rate of the drugs, respectively. Data shown are means ± SD (*n* = 3). Comparison of mean values **(A)** between two groups were analyzed by Student’s t test. *p < 0.05; **p < 0.01; ***p < 0.001.

Virus titration assay revealed that all three compounds inhibited the production of infectious virions in a dose dependent manner (Figure 2A). Similar inhibitory effects were observed on the intracellular and extracellular vRNA levels and the production of the viral nucleoprotein under drug treatment (Supplementary Figure S1). The inhibitory activity of these selected compounds on SFTSV infection was further validated in additional cell lines, including Huh7 and HUVEC cells, which are susceptible to SFTSV infection (Sun et al., 2014). In consistent with the result on Vero cells, anti-SFTSV effects were observed in a dose dependent manner (Figures 2B,C). HUVEC cells are vascular endothelial cells, which might represent the vascular endothelial injury induced by SFTSV infection (Li et al., 2018), thus the subsequent experiments were performed mainly on HUVEC cells. The SI of punicalin and toosendanin on HUVEC cells were determined to be >100 and 4,800, respectively (Figure 2D). These two compounds were subjected to further investigation.

### Toosendanin Inhibits SFTSV Infection at the Entry Step

**FIGURE 3:**
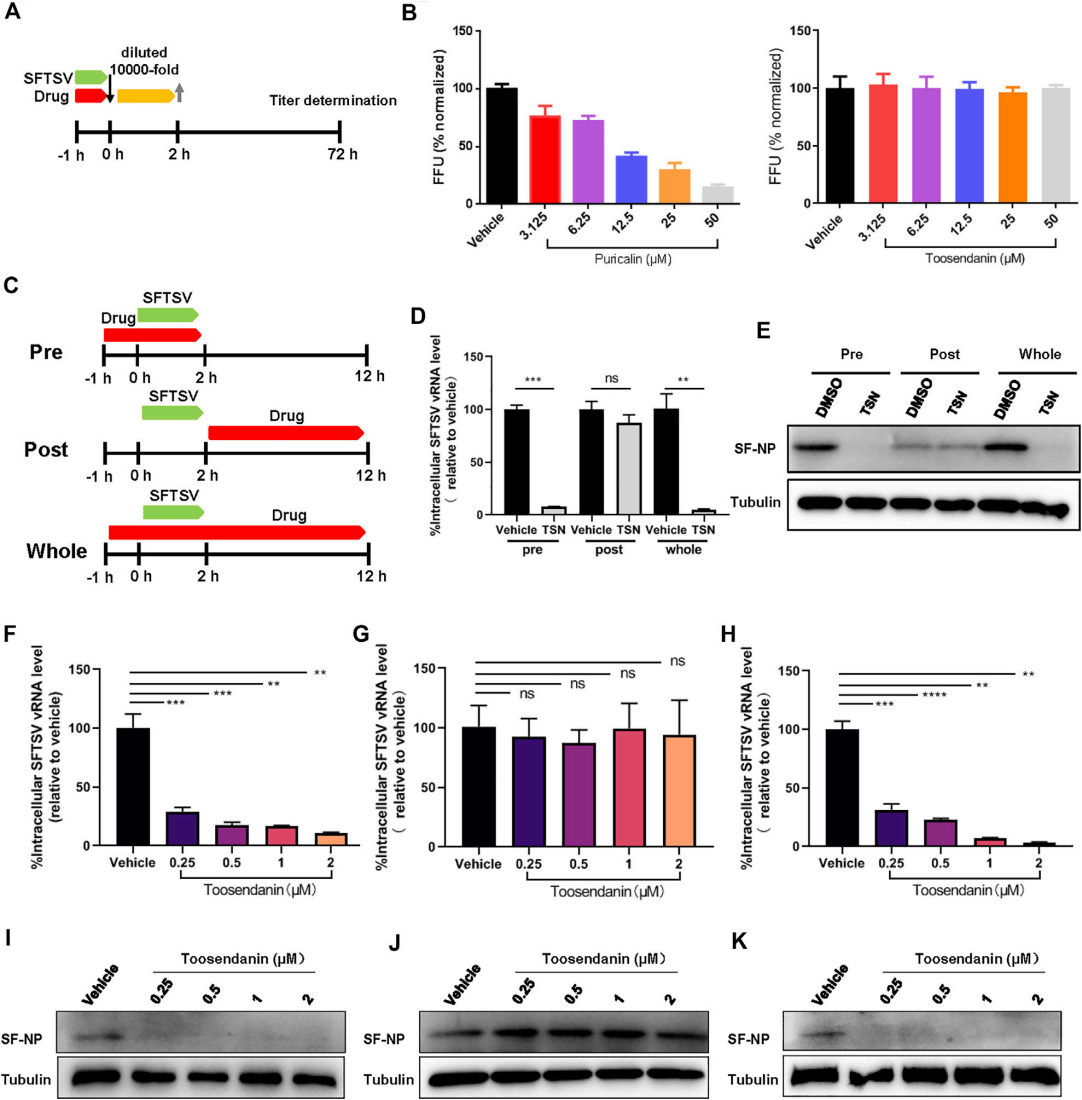
Toosendanin inhibits the entry step of SFTSV infection. **(A)** Schematic diagram of the virucidal effect assay. **(B)** The virucidal effect of punicalin and toosendanin. SFTSV was incubated with indicated concentrations of punicalin and toosendanin for 37°C for 1 h. The mixture was diluted 10,000 fold and subjected to titer determination. **(C)** Schematic diagram of time-of-addition assay of toosendanin. **(D,E)** HUVEC cells infected with SFTSV at an MOI of 2 for 2 h were treated with toosendanin (1 μM) for 1 h pre-infection (pre), post-infection (post), and whole time (whole). Infected cells were collected at 12 h post infection for viral RNA quantification **(D)** and western blot **(E)**. **(F–K)** Time-of-addition assay was conducted under the indicated concentrations of toosendanin. The inhibitory effect of toosendanin on pre **(F,I)**, post **(G,J)**, and whole **(H,K)** were determined by qRT-PCR and western blot. TSN, toosendanin. Data shown are means ± SD (*n* = 3). Comparison of mean values **(D,F,G,H)** between two groups were analyzed by Student’s t test. **p < 0.01; ***p < 0.001; ****p < 0.0001; ns, no significance.

Next, we determined the inhibition mechanisms of punicalin and toosendanin. To investigate whether punicalin and toosendanin have virucidal effect on SFTSV virions, SFTSV was incubated with the compounds at the indicated concentrations for 1 h, and then the infectivity of the treated viruses were analyzed (Figure 3A). As shown in Figure 3B, punicalin directly inactivated virions in a dose dependent manner and the treatment of toosendanin demonstrated no virucidal effect on SFTSV.

A time-of-addition assay was performed to identify the SFTSV replication stage that was inhibited by toosendanin (Figure 3C). Toosendanin was added pre or post SFTSV infection and the infected cells were harvested at 12 hpi. Intracellular level of vRNA and expression level of viral protein were determined by quantitative RT-PCR and western blot, respectively. As shown in Figures 3D,E, vRNA and viral protein expression decreased significantly when toosendanin was added prior to but not post SFTSV infection, indicating that the drug inhibited SFTSV during the entry step. Similar results were observed when using a serial concentrations of drug (0.25–2 μM), confirming that toosendanin inhibits the entry rather than the viral replication stage (Figures 3F–K). Furthermore, the mini-genome (MG) assay revealed that toosendanin treatment did not affect the viral RNA replication and transcription (Supplementary Figure S2A).

### Toosendanin Inhibits the Internalization of SFTSV

**FIGURE 4:**
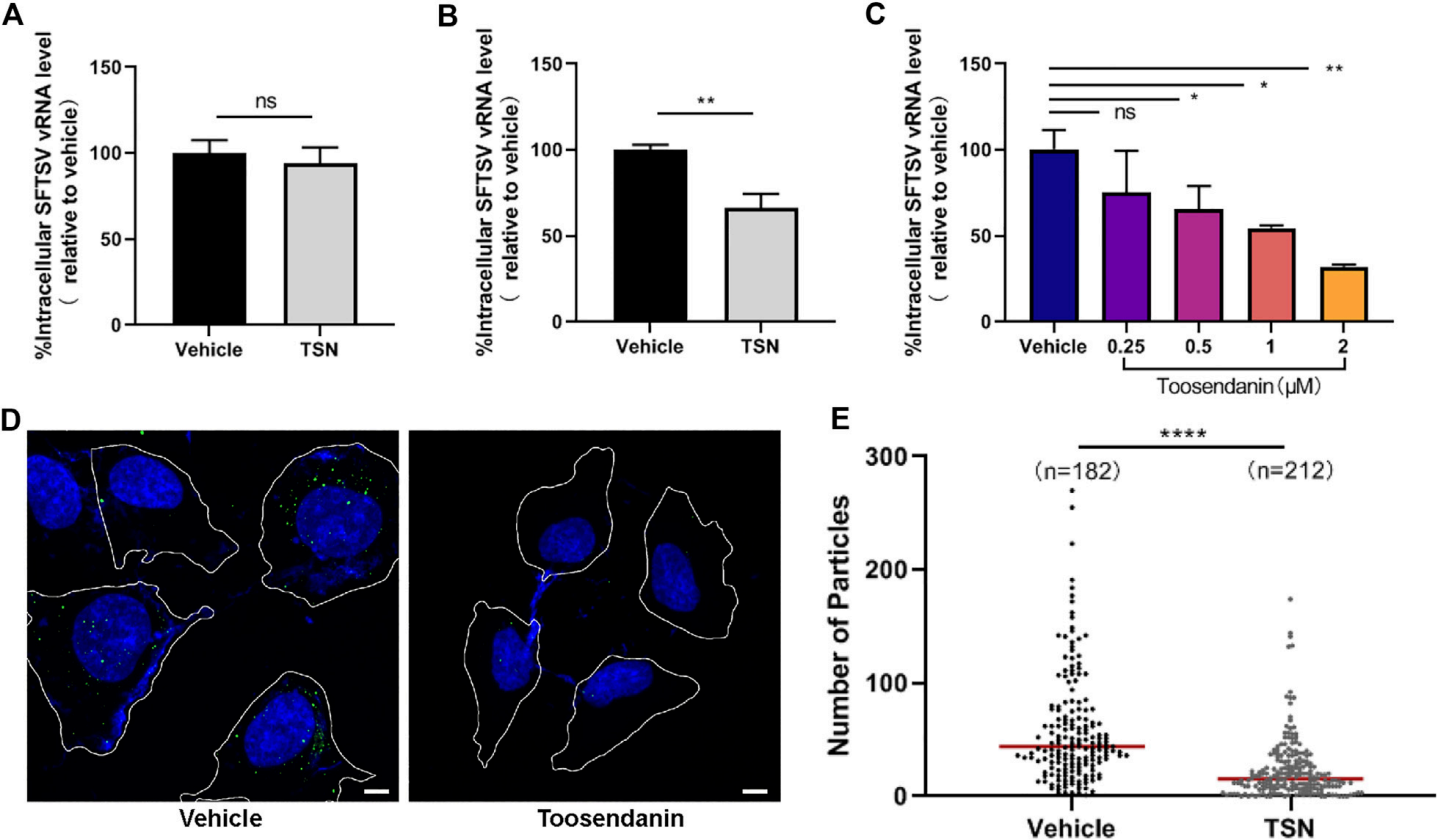
Toosendanin inhibits SFTSV internalization. **(A)** Effect of toosendanin on SFTSV binding. HUVEC cells were pretreated with toosendanin (1 μM) or vehicle (DMSO) at 37°C for 1 h, chilled at 4°C for 15 min, and incubated with SFTSV (MOI = 5) at 4°C for 2 h. The unbound virions were removed with pre-cooled PBS, and cells were harvested for bound virions quantification. **(B,C)** Effect of toosendanin on SFTSV internalization. HUVEC cells pre-treated with 1 μM toosendanin **(B)** or a series concentration of toosendanin **(C)** for 1 h were chilled at 4°C for 15 min and incubated with SFTSV (MOI = 5) for 1 h. Cells were washed with pre-cooled PBS to eliminate the unbound virions and incubated at 37°C for 3 h in the presence of ammonium chloride (20 mM), then washed and treated with trypsin. The relative level of internalized virions was determined by qRT-PCR. **(D)** Effect of toosendanin on SFTSV internalization was analyzed by immunofluorescence. Cell boundaries (white lines), NP staining (green), DAPI staining (blue). Bars represent 5 μm. **(E)** Quantification of intracellular virions captured in **(D)**. Median value (red line), number of cells under quantification (n). TSN, toosendanin. Data shown are means ± SD (*n* = 3). Comparison of mean values **(A,B,C)** between two groups were analyzed by Student’s t test. *p < 0.05; **p < 0.01; ns, no significance. Comparison of median values **(E)** between two groups were analyzed by Mann-Whitney test. ****p < 0.0001.

The virus entry can be further divided into binding, internalizing and fusion events. We next investigated the specific viral entry event that is targeted by toosendanin. First, HUVEC cells pre-treated with toosendanin or vehicle (DMSO) were incubated with SFTSV at 4°C for 1 h to synchronize the binding process of virus. The un-bound virions were removed by three times of washing, and the relative level of virions bound to cells was analyzed. As shown in Figure 4A, vRNA level in toosendanin treated group was comparable to the vehicle control, suggesting that toosendanin did not inhibit SFTSV binding. To evaluate the effect of toosendanin on viral internalization, HUVEC cells were infected with SFTSV in the presence of toosendanin or vehicle, and the ammonium chloride was added to prevent low-pH-dependent membrane fusion to block viral entry at the step of fusion. The relative intracellular vRNA was significantly reduced in the toosendanin treated cells, compared with the vehicle control (Figure 4B). Furthermore, toosendanin treatment showed a dose dependent inhibition effect on accumulation of vRNA (Figure 4C). Virus internalization was further visualized with microscopy analysis. Internalized viral particles were stained with polyclonal antibody against the viral protein NP and images of the cross-section of infected cells were acquired. The intracellular viral particles were analyzed and quantified. As shown in Figures 4D,E, toosendanin treatment significantly reduced the amount of internalized viral particles. To evaluate whether toosendanin treatment inhibits viral fusion, syncytium formation assay was performed. Briefly, infected cells were treated with low pH medium, followed by addition of different concentrations of toosendanin and the syncytium formation was monitored. No significant difference of syncytium formation was observed between toosendanin treated and vehicle treated control (Supplementary Figure S2B), indicating that toosendanin did not inhibit the viral fusion step. These results suggested that toosendanin inhibited the internalization event of SFTSV infection.

### Toosendanin Demonstrates Anti-SFTSV Efficacy *in vivo*

**FIGURE 5:**
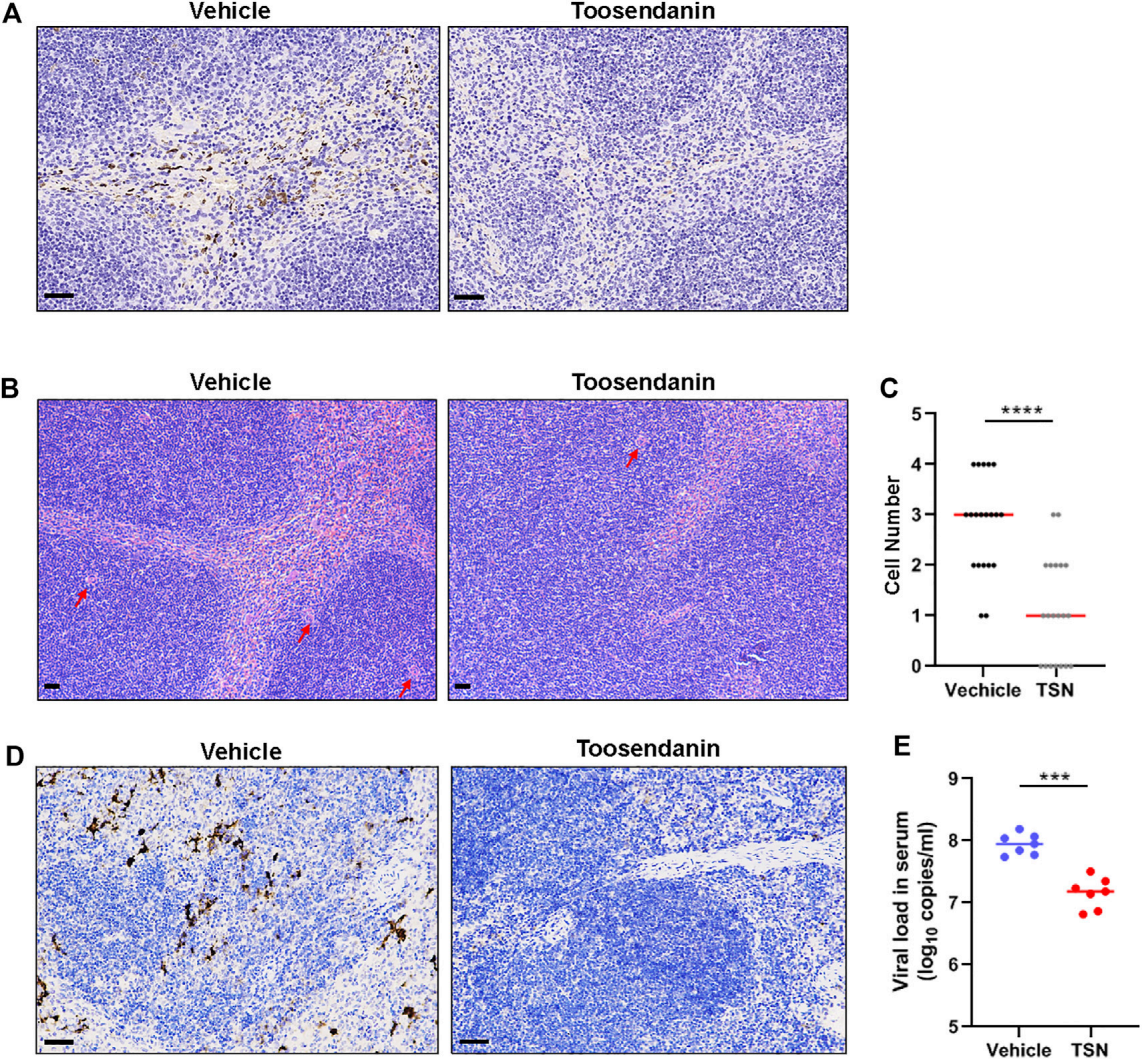
Toosendanin inhibits SFTSV infection in mouse models. **(A–C)** C57BL/6 were pretreated with toosendanin for 3 days and infected intraperitoneally with SFTSV (10^5^ FFU). Toosendanin (1 mg/kg/d) or vehicle was administered daily. Spleen samples were collected from mice at 3 days post infection for immunostaining of viral nucleocapsid protein **(A)**, and H&E staining **(B)**. **(C)** Quantification of megakaryocytes in spleens. **(D,E)** C57BL/6 mice were treated with anti-IFNAR1 IgG 1 day prior to the infection and intraperitoneally infected with 2000 FFU of SFTSV. Toosendanin or the same volume of vehicle was given to mice by intraperitoneal injection with the dose of 4 mg/kg/d 1 h post infection. Spleen and serum samples were harvested for immunohistochemistry (D) and viral load determination **(E)**. TSN, toosendanin. **(A,B,D)** Bars represent 50 μm. Comparison of median values **(C)** between two groups was analyzed by Mann-Whitney test. ****p < 0.0001. Comparison of mean values **(E)** between two groups were analyzed by Mann-Whitney test. **p < 0.01; ***p < 0.001.

The anti-SFTSV effect of toosendanin was further evaluated on C57BL/6 mice, an established mouse model of SFTSV infection (Jin et al., 2012). Eight-week old female C57BL/6 mice were pretreated with toosendanin through intraperitoneal administration for 3 days and infected intraperitoneally with SFTSV. Toosendanin or vehicle was administered on a daily basis. Toosendanin treatment and/or SFTSV infection did not lead to weight loss in this infected mouse model (Supplementary Figure S3). Spleen samples were collected from mice treated with toosendanin (*n* = 5) or vehicle (*n* = 5) and the viral antigen was determined by immunohistochemistry staining, less viral protein positive cells was detected in the spleen sections of mice treated with toosendanin (Figure 5A). The infection of SFTSV lead to an increase of megakaryocytes in the spleen of infected mice, which is correlated to the compensatory phenomenon of circulating platelet (PLT) depletion induced by SFTSV infection (Jin et al., 2012). The hematoxylin and eosin (H&E) staining of spleen sections revealed that toosendanin treatment reduced the megakaryocytes accumulation in the spleens of infected mice compared with the vehicle control (Figures 5B,C). The results indicated that toosendanin treatment can inhibit SFTSV replication and reduce histopathological changes in the infected C57BL/6 mouse model. Similar results were also observed in an anti-IFNAR1 IgG treated C57BL/6 mouse model, in which the administration of toosendanin reduced the antigen of SFTSV nucleoprotein in the spleen sections of infected mice (Figure 5D). Furthermore, viral load in the serum of toosendanin treated mice was significantly lower than that of the mice in the vehicle control group (Figure 5E).

### Toosendanin Inhibits the Replication of Rift Valley Fever Virus and SARS-CoV-2

**FIGURE 6:**
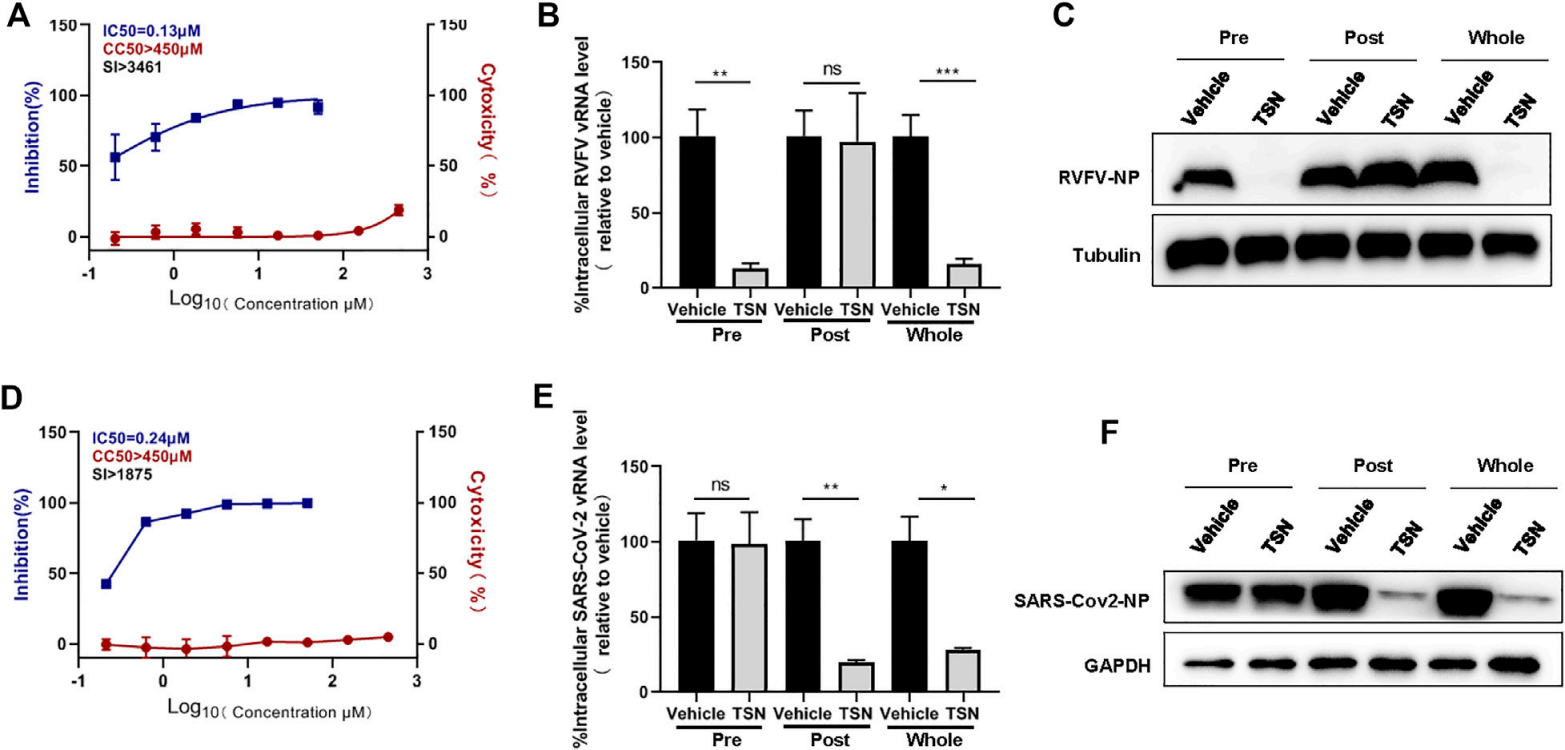
Inhibitory effects of toosendanin on Rift Valley fever virus (RVFV) and SARS-CoV-2 infection. **(A)** The dose-dependent inhibitory activity of toosendanin against RVFV infection on HUVEC cells. HUVEC cells pre-treated with indicated concentrations of toosendanin were infected with RVFV (MOI = 2), cell supernatants were harvested for viral RNA quantification by qRT-PCR. Cell viability on HUVEC cells was determined by CCK-8. The left and right *Y*-axis represent inhibition and cytotoxicity rate of the toosendanin. **(B,C)** Time-of-addition assay of toosendanin on RVFV infection. HUVEC cells infected with RVFV (MOI = 2) for 2 h were treated with toosendanin (1 μM) for 1 h pre-infection (pre), post-infection (post), and whole time (whole). Infected cells were collected at 12 h post infection for viral RNA quantification **(B)** and western blot **(C)**. **(D)** The dose-dependent inhibitory activity of toosendanin against SARS-CoV-2 infection on Vero-E6 cells. Vero-E6 cells pre-treated with indicated concentrations of toosendanin were infected with SARS-CoV-2 (MOI = 0.01), cell supernatants were harvested for viral RNA quantification by qRT-PCR. Cell viability on Vero-E6 cells was determined by CCK-8. The left and right *Y*-axis represent inhibition and cytotoxicity rate of toosendanin. **(E,F)** Time-of-addition assay of toosendanin on SARS-CoV-2 infection. Vero-E6 cells infected with SARS-CoV-2 (MOI = 0.01) for 1 h were treated with toosendanin (1 μM) for 1 h pre-infection (pre), post-infection (post), and whole time (whole). Infected cells were collected at 24 h post infection for viral RNA quantification **(E)** and western blot **(F)**. TSN, toosendanin. Data shown are means ± SD (*n* = 3). Comparison of mean values **(B,E)** between two groups were analyzed by Student’s t test. *p < 0.05; **p < 0.01; ***p < 0.001; ns, no significance.

In order to determine whether toosendanin has a broad anti-viral effect, we analyzed the potential inhibitory effect of this compound against another bunyavirus, Rift Valley fever virus (RVFV) and the recently emerging SARS-CoV-2. Toosendanin showed potent inhibition on the infection of RVFV (IC_50_ = 0.13 μM, CC_50_ > 450 μM, SI > 3,461 μM) and SARS-CoV-2 (IC_50_ = 0.24 μM, CC_50_ > 450 μM, SI > 1875 μM) (Figures 6A,D). Time-of-addition assay indicated that toosendanin inhibited the entry step of RVFV (Figures 6B,C), which is similar with the inhibition mechanism against SFTSV infection. Differently, in the case of SARS-CoV-2, toosendanin revealed inhibitory effect when added 1 h post infection (Figures 6E,F), suggesting that it targets the viral replication event.

## Discussion

SFTSV is an emerging tick-borne virus causing severe infectious disease with high case-fatality. Currently, no effective drug against SFTSV infection has been approved for the treatment of SFTSV patients. In this study, we performed high-throughput screening of a natural extracts library and three compounds were identified to exhibit high anti-viral activity against SFTSV infection *in vitro*. Mechanistic investigation of the most potent compound, toosendanin, revealed that the compound inhibits viral internalization in the early stage of infection. Furthermore, treatment with toosendanin reduced SFTSV infection and pathological changes in the SFTSV infected mouse models.

Natural extracts provide a variety of small compounds for drug development in clinical use, including the treatment of infectious diseases (Hensel et al., 2020). A major advantage of natural extracts to be developed as anti-viral drug is the large pool of natural compounds with various structures, which provide the possibility to target different viral components and replication steps against different viruses (Mohan et al., 2020). Recent studies revealed that lignans are potential candidates for the development of anti-viral drugs, which demonstrated high anti-viral activity against HIV and human papillomavirus (HPV) *in vitro* and hepatitis B virus in clinical settings (Schröder et al., 1990; Bao and Liu, 2008; Zalesak et al., 2019). Also, Guo et al. performed a high-throughput screening of a library consisting of natural extracts to select compounds against Japanese Encephalitis Virus infection, and identify two Na/K-ATPase inhibitors as potential candidates for exerting anti-JEV activity both *in vitro* and *in vivo* (Guo et al., 2020). These reports and the results in this study indicate that screening with natural extracts is a feasible strategy for identifying compound for anti-viral drug development.

Toosendanin is a triterpenoid saponin extracted from the fruit of *Melia toosendan Sieb et Zucc*, which has been demonstrated to selectively block the release of neurotransmitter (Shi and Chen, 1999), and showed anti-inflammatory and anti-botulismic effect (Xie et al., 2008; Fang and Cui, 2011). Recent studies revealed the broad anti-cancer activities of toosendanin against glioblastoma, osteosarcoma, gastric carcinoma and breast cancer (Cao et al., 2016; Wang et al., 2017b; Zhang et al., 2017; Kai et al., 2018). Toosendanin has been used in the clinics as anthelmintic to expel parasites from alimentary tract in China (Shi and Li, 2007). Toosendanin was reported to suppress hepatitis C virus infection through the enhancement of alpha interferon signaling pathway and inhibit influenza A virus replication by interfering the nuclear localization of viral polymerase PA protein (Watanabe et al., 2011; Jin et al., 2019). Here, we report that toosendanin inhibits SFTSV infection both *in vitro* and *in vivo*, by interfering with the SFTSV entry step. In addition, toosendanin demonstrated inhibitory effect towards the entry step of RVFV and the replication stage of the emerging SARS-CoV-2, indicating its broad anti-viral effect through different mechanisms. Toosendanin is an agonist of L-type voltage-dependent calcium channels, which might participate in the regulation of intracellular calcium homeostasis. Our recent work revealed that the regulation of intracellular Ca^2+^ concentration is involved in the entry step of SFTSV infection (Li et al., 2019a). It is recently reported that several calcium channel blockers show inhibitory effects against a number of viruses including bunyaviruses (Li et al., 2019a), Marburg virus (DeWald et al., 2018), JEV (Wang et al., 2017a) and SARS-CoV-2 (Zhang et al., 2020). Whether Toosendanin’s broad anti-viral effect against SFTSV, RVFV and SARS-CoV-2 is associated with regulation of intracellular calcium concentration worth further investigation.

Due to the high mutation frequency of RNA viruses, anti-viral drugs targeting viral proteins often lead to drug resistant mutants that escape the anti-viral effect (Andrei and De Clercq, 1993; Lingappa et al., 2013). In order to investigate whether toosendanin treatment can lead to drug-resistant mutations, serial passaging of SFTSV in the presence of toosendanin was performed. However, no resistant variant was recovered after 20 serial passages (data not shown). This result indicated that toosendanin might target host factors/pathways to inhibit SFTSV replication and may represent a host-directed anti-viral compound. The host-directed therapy (HDT) is an emerging strategy in the field of anti-viral drug development (Kaufmann et al., 2018). A major advantage of HDT is to interfere with host cell factors that are required by a pathogen for replication or persistence, to enhance protective immune responses against a pathogen, to reduce exacerbated inflammation and to balance immune reactivity (Kaufmann et al., 2018). Whether toosendanin can be developed as a host-directed drug that can be applied in the clinical settings as a broad anti-viral drug would worth further investigation.

## Data Availability

The original contributions presented in the study are included in the article/Supplementary Material, further inquiries can be directed to the corresponding authors.
