# Supplementary figures and images for: Screening of a Small Molecule Compound Library Identifies Toosendanin as an Inhibitor Against Bunyavirus and SARS-CoV-2
Source: Front Pharmacol. 2021 Nov 11;12:735223. doi: 10.3389/fphar.2021.735223 (PMC8632254; doi:10.3389/fphar.2021.735223)

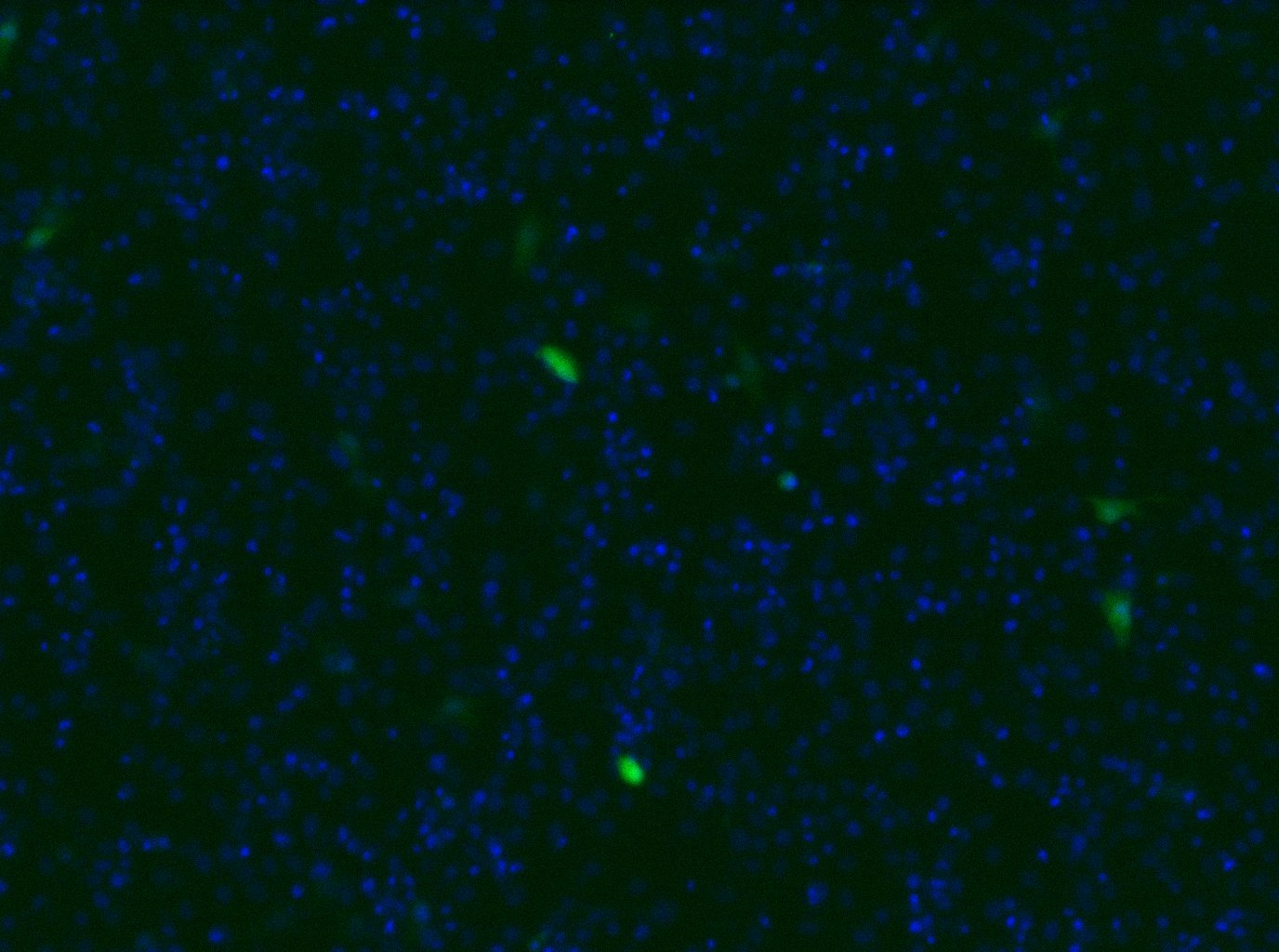

Supplement: Supplementary file 1 [file DataSheet3.ZIP › Sup Fig2A microscopy images/Benidipine-Composite.jpg]

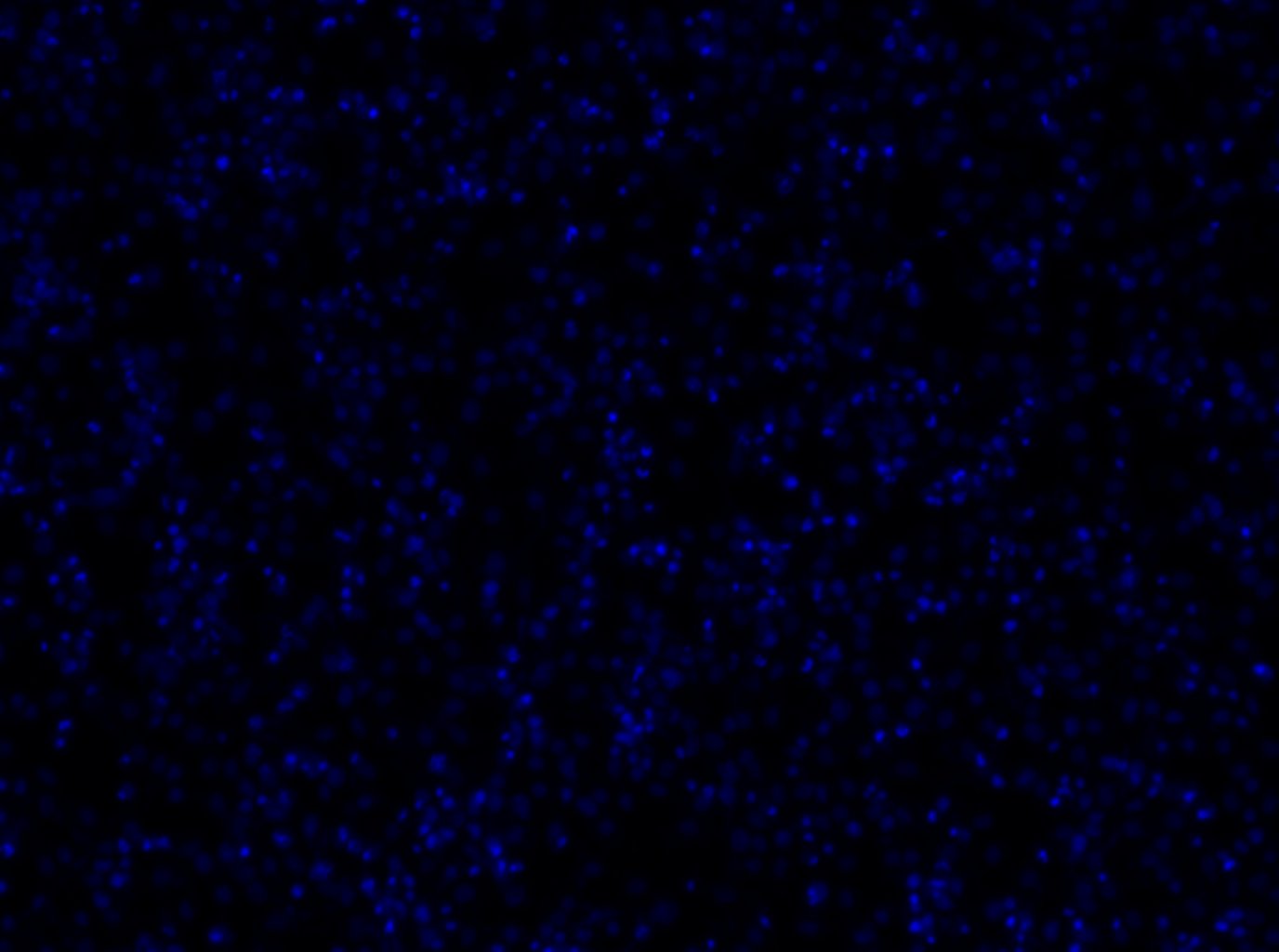

Supplement: Supplementary file 1 [file DataSheet3.ZIP › Sup Fig2A microscopy images/Benidipine-DAPI.jpg]

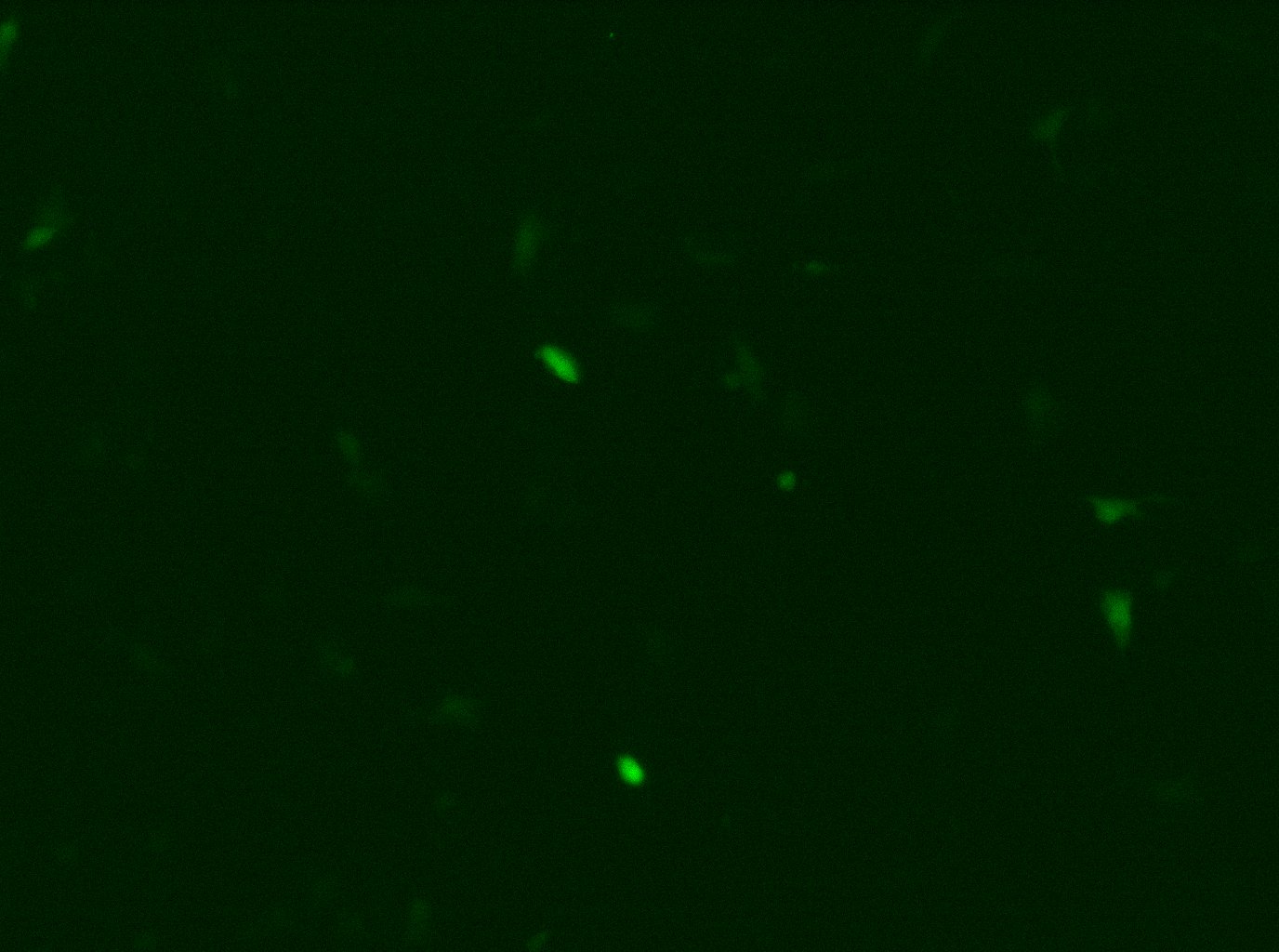

Supplement: Supplementary file 1 [file DataSheet3.ZIP › Sup Fig2A microscopy images/Benidipine-eGFP.jpg]

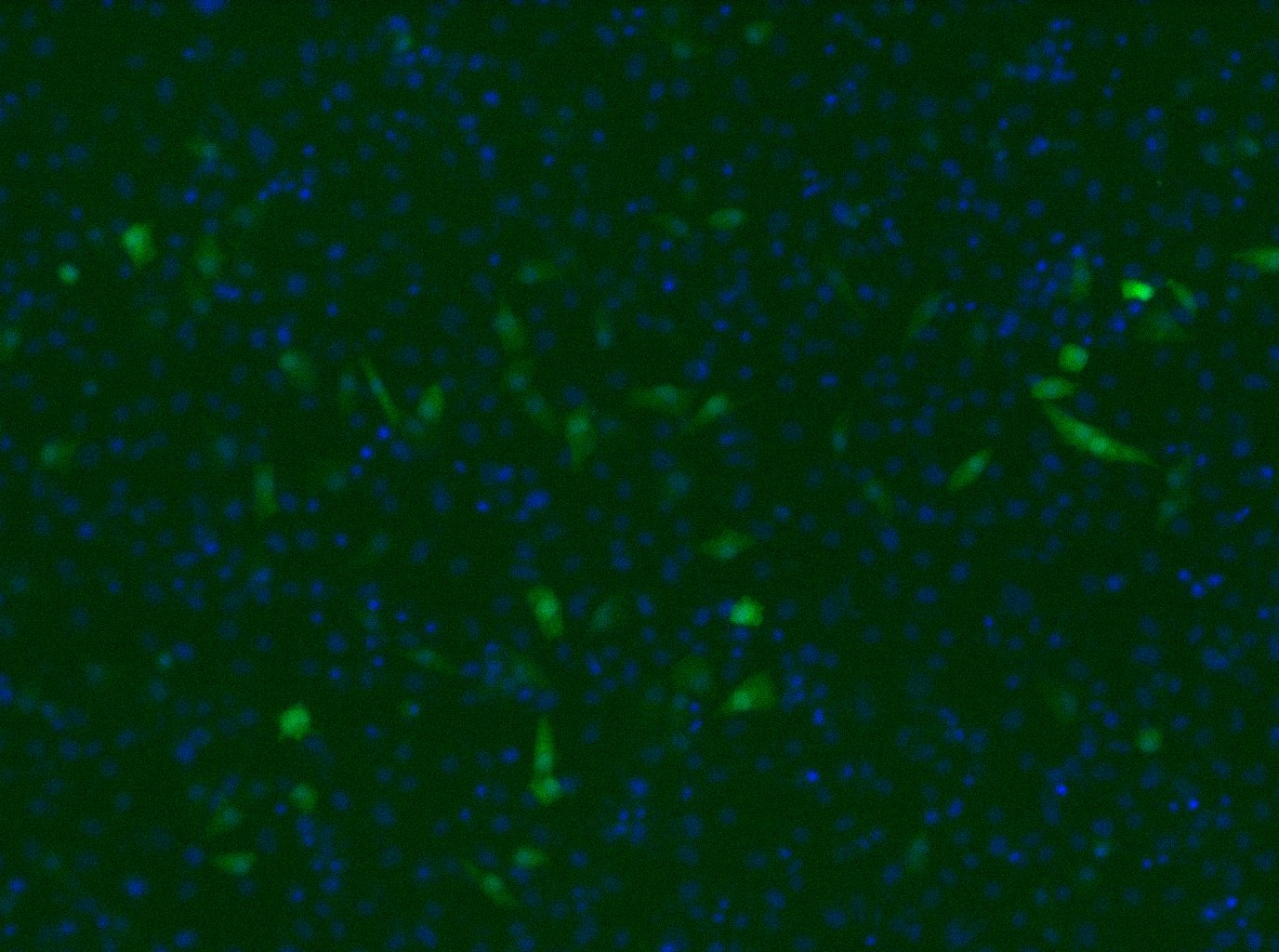

Supplement: Supplementary file 1 [file DataSheet3.ZIP › Sup Fig2A microscopy images/Toosendanin-10μM-Composite.jpg]

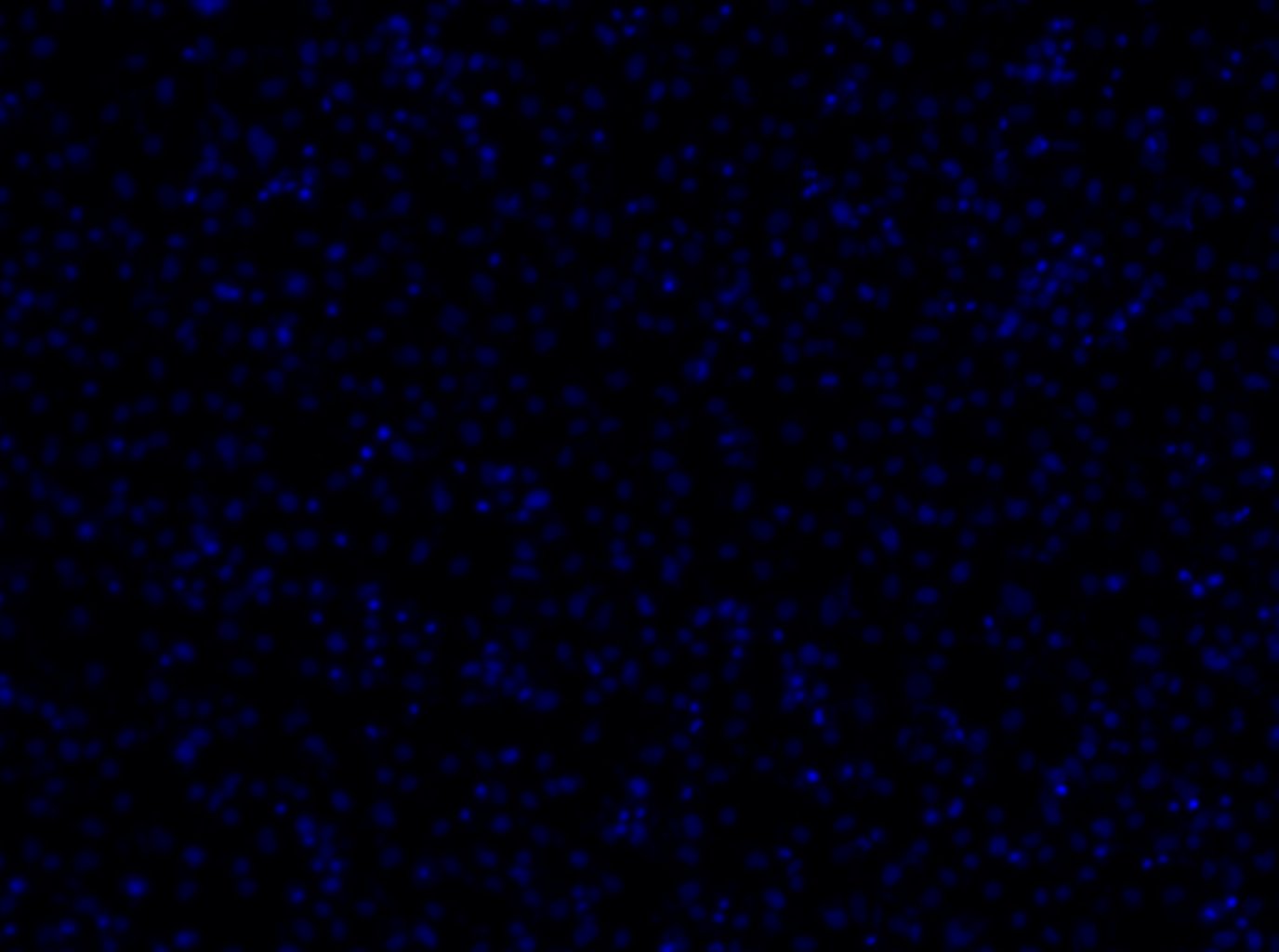

Supplement: Supplementary file 1 [file DataSheet3.ZIP › Sup Fig2A microscopy images/Toosendanin-10μM-DAPI.jpg]

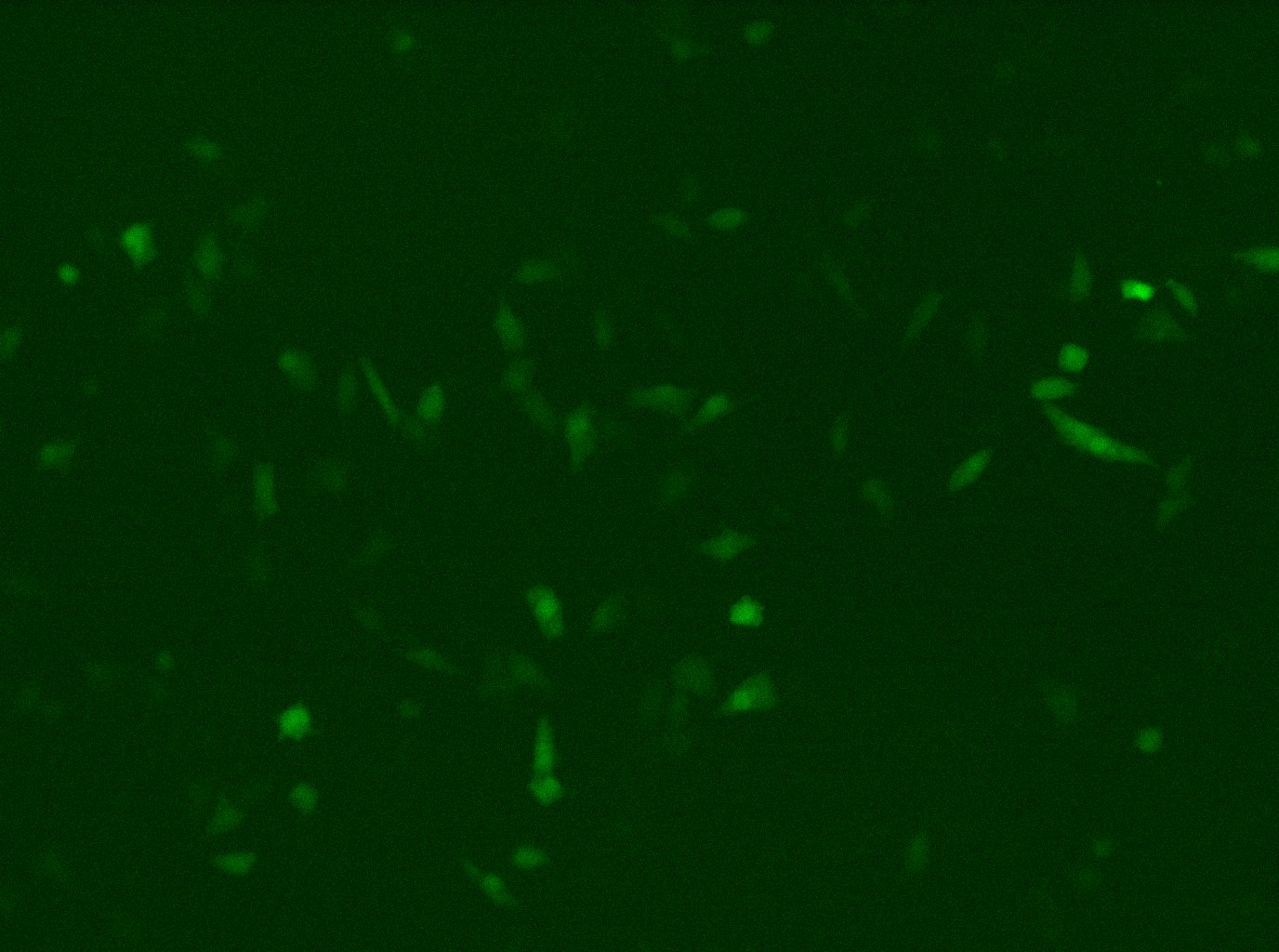

Supplement: Supplementary file 1 [file DataSheet3.ZIP › Sup Fig2A microscopy images/Toosendanin-10μM-eGFP.jpg]

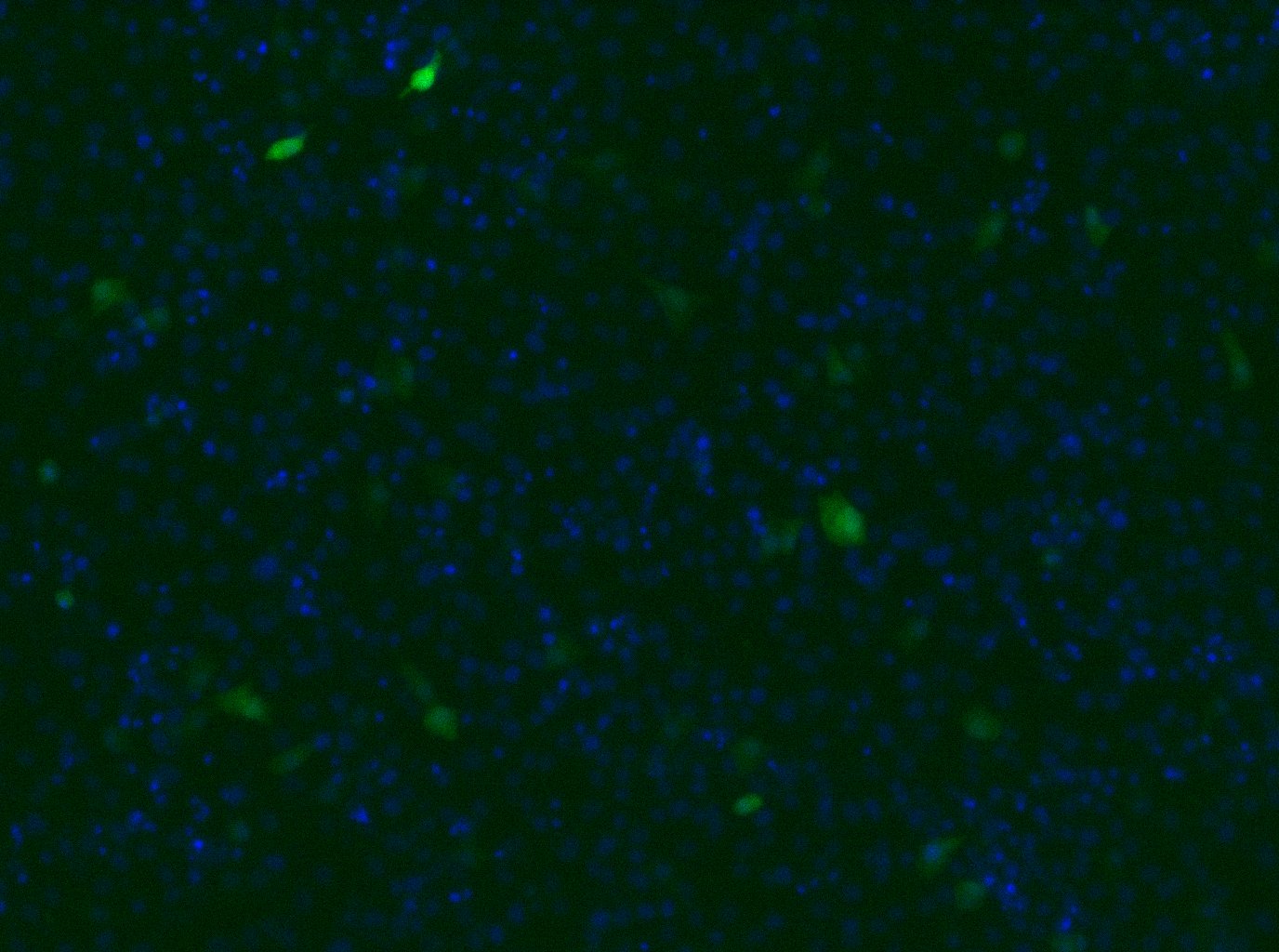

Supplement: Supplementary file 1 [file DataSheet3.ZIP › Sup Fig2A microscopy images/Toosendanin-1μM-Composite.jpg]

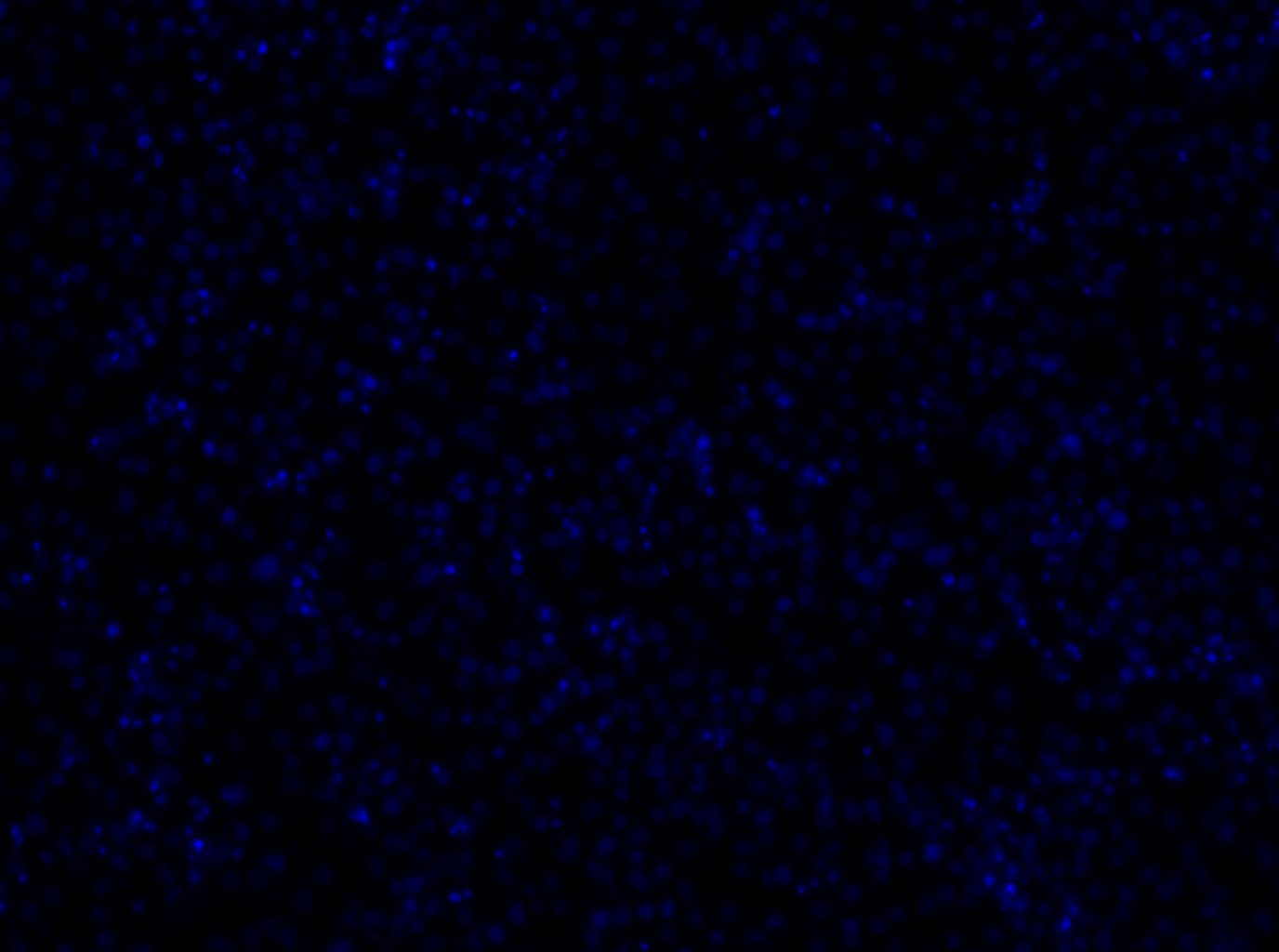

Supplement: Supplementary file 1 [file DataSheet3.ZIP › Sup Fig2A microscopy images/Toosendanin-1μM-DAPI.jpg]

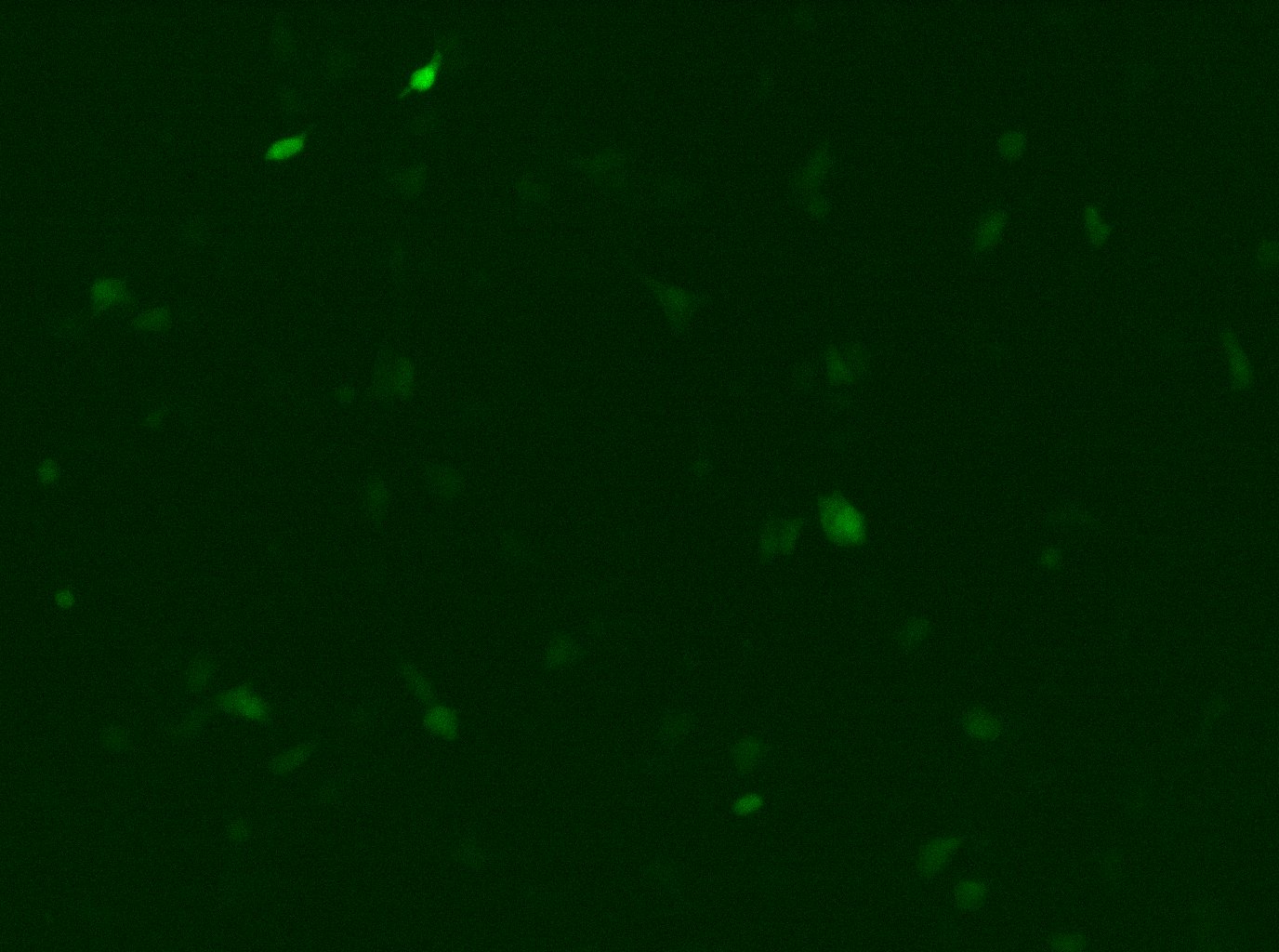

Supplement: Supplementary file 1 [file DataSheet3.ZIP › Sup Fig2A microscopy images/Toosendanin-1μM-eGFP.jpg]

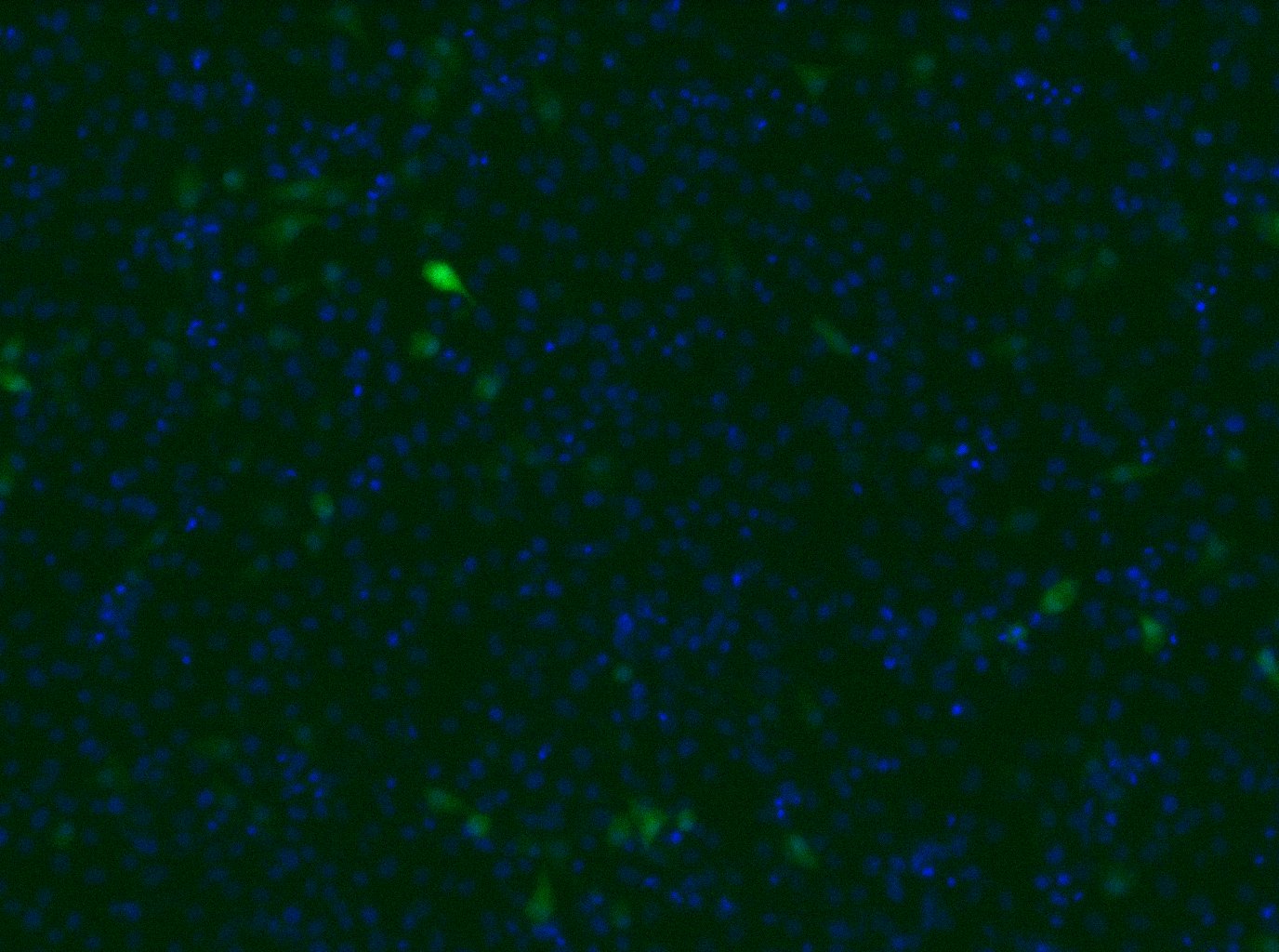

Supplement: Supplementary file 1 [file DataSheet3.ZIP › Sup Fig2A microscopy images/Toosendanin-5μM-Composite.jpg]

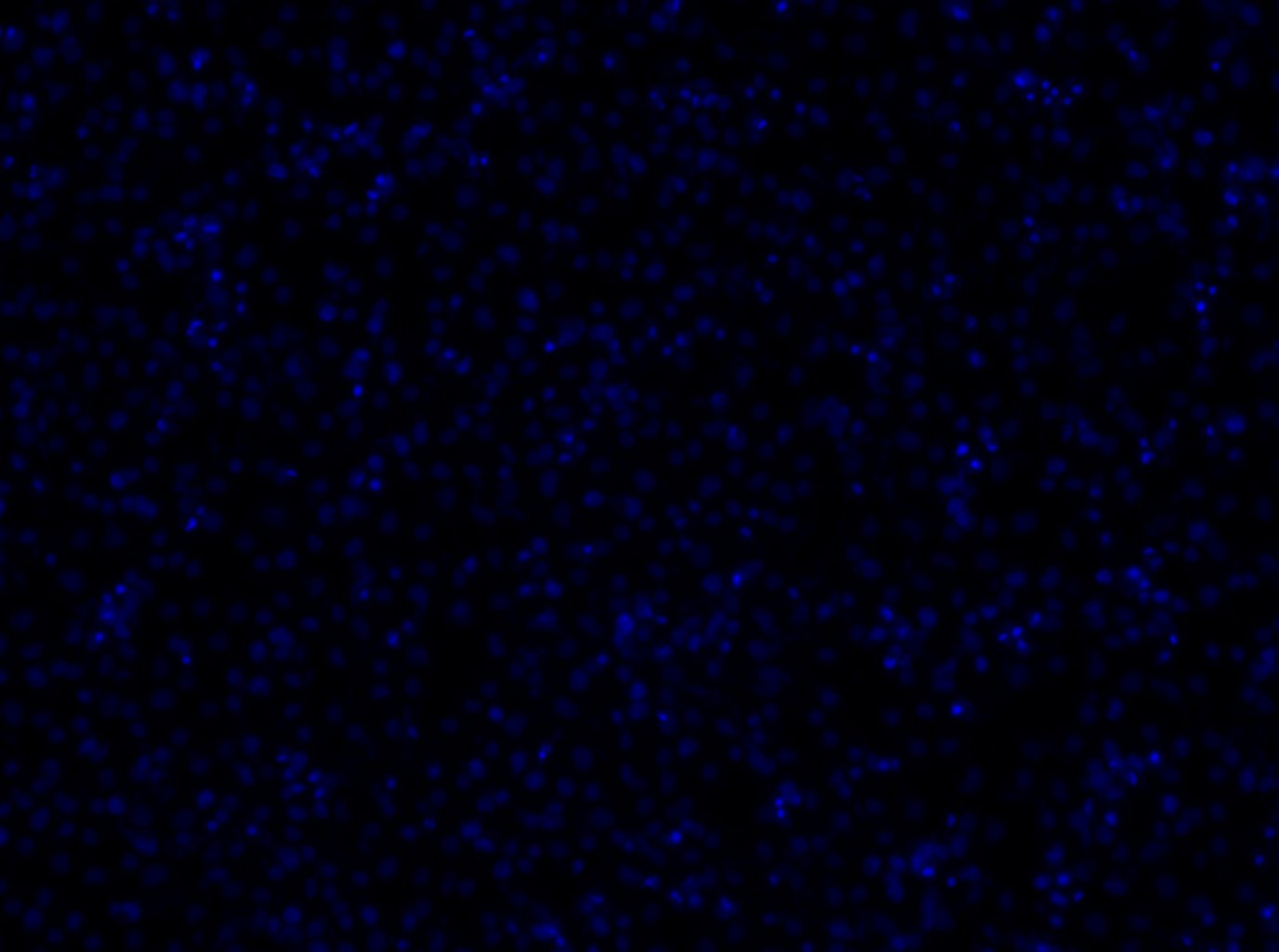

Supplement: Supplementary file 1 [file DataSheet3.ZIP › Sup Fig2A microscopy images/Toosendanin-5μM-DAPI.jpg]

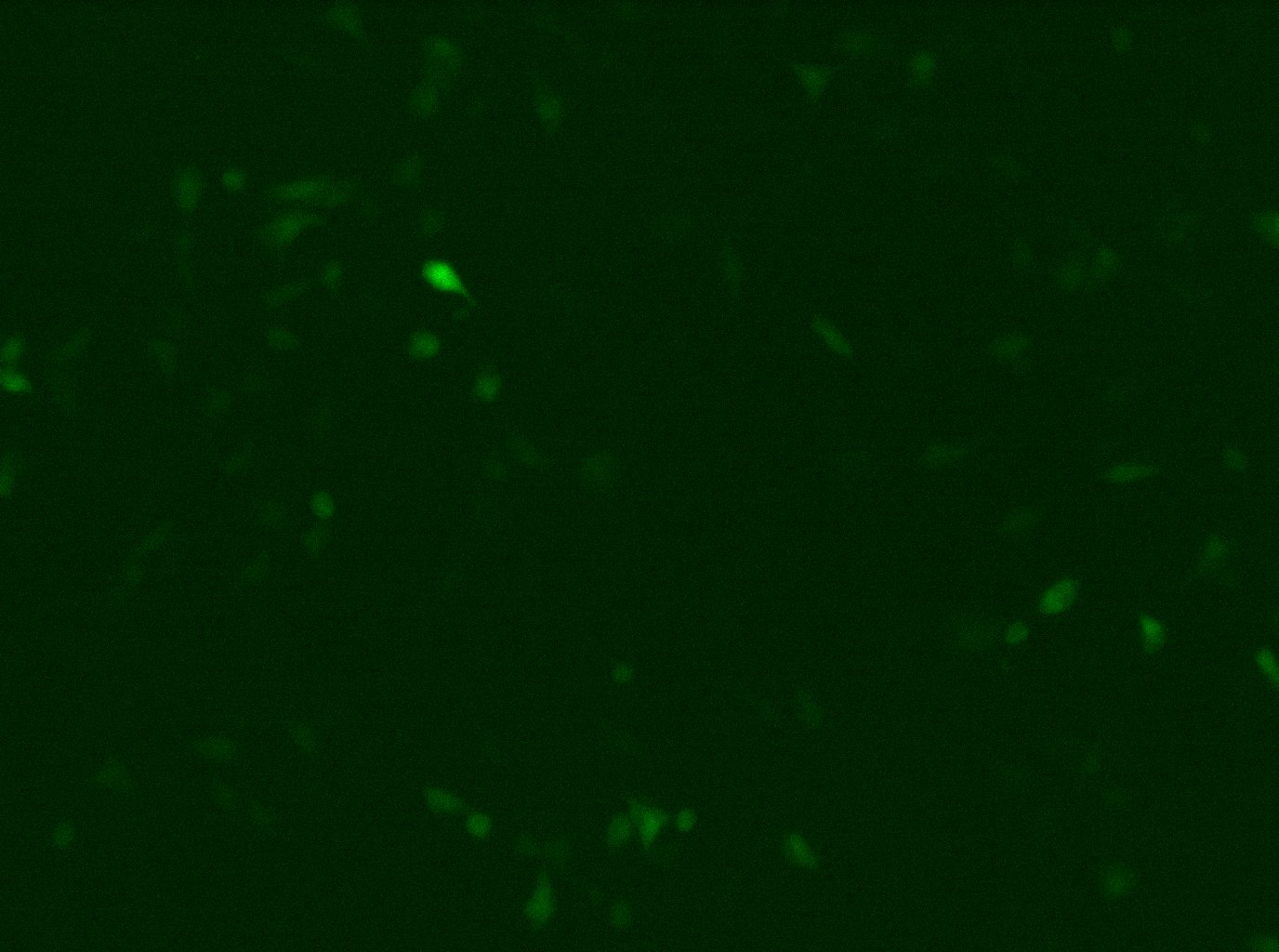

Supplement: Supplementary file 1 [file DataSheet3.ZIP › Sup Fig2A microscopy images/Toosendanin-5μM-eGFP.jpg]

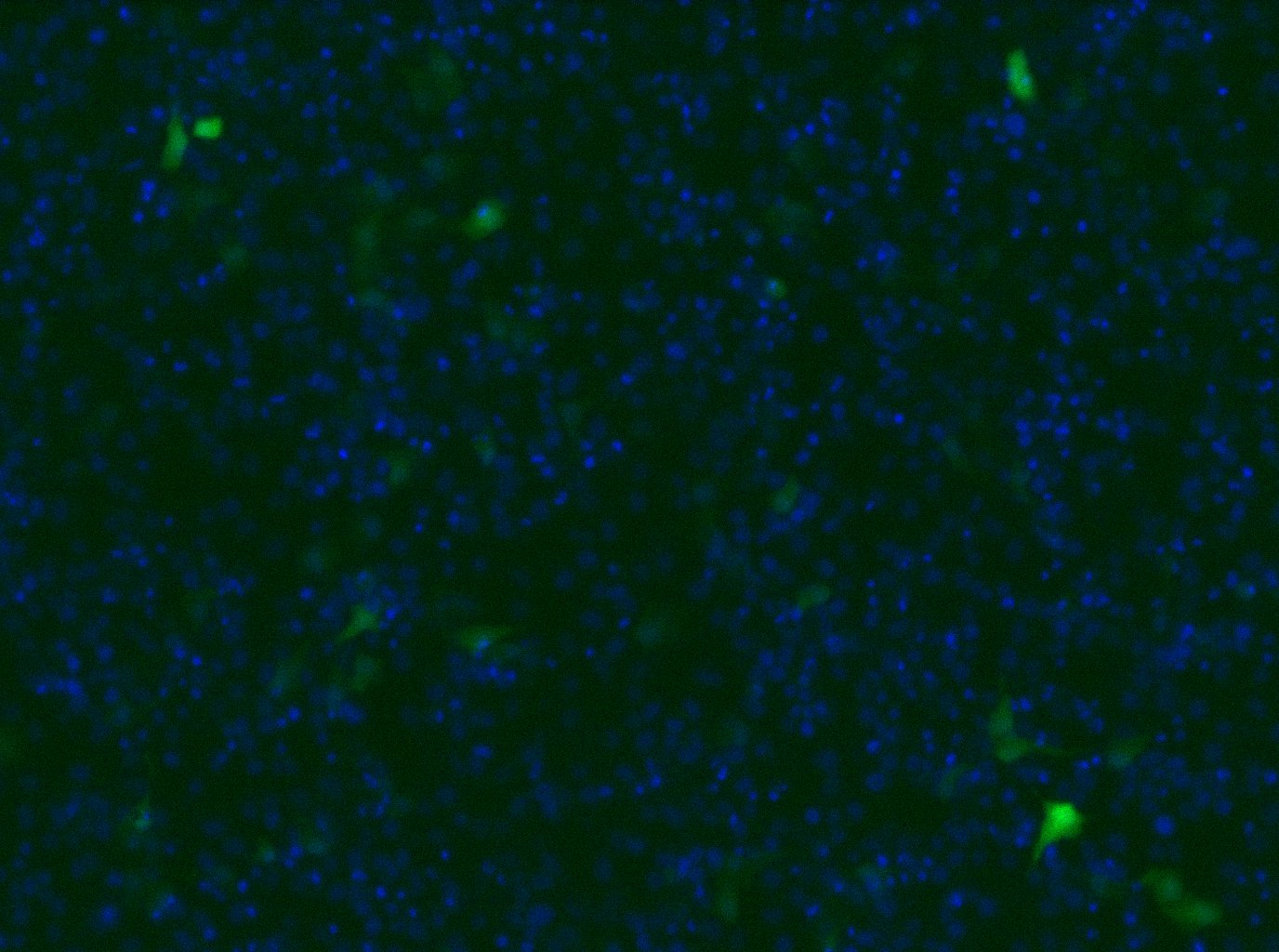

Supplement: Supplementary file 1 [file DataSheet3.ZIP › Sup Fig2A microscopy images/Vehicle-Composite.jpg]

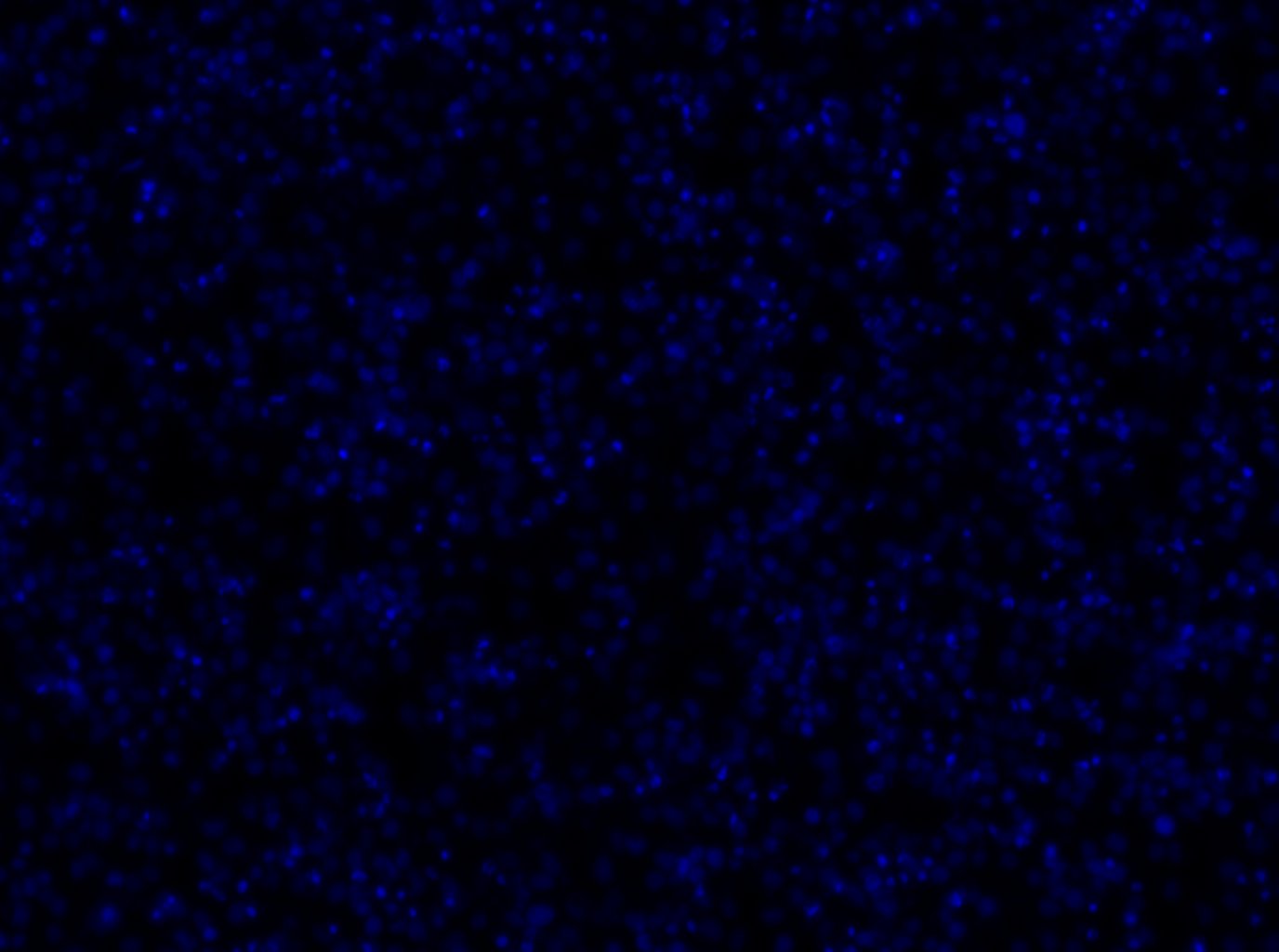

Supplement: Supplementary file 1 [file DataSheet3.ZIP › Sup Fig2A microscopy images/Vehicle-DAPI.jpg]

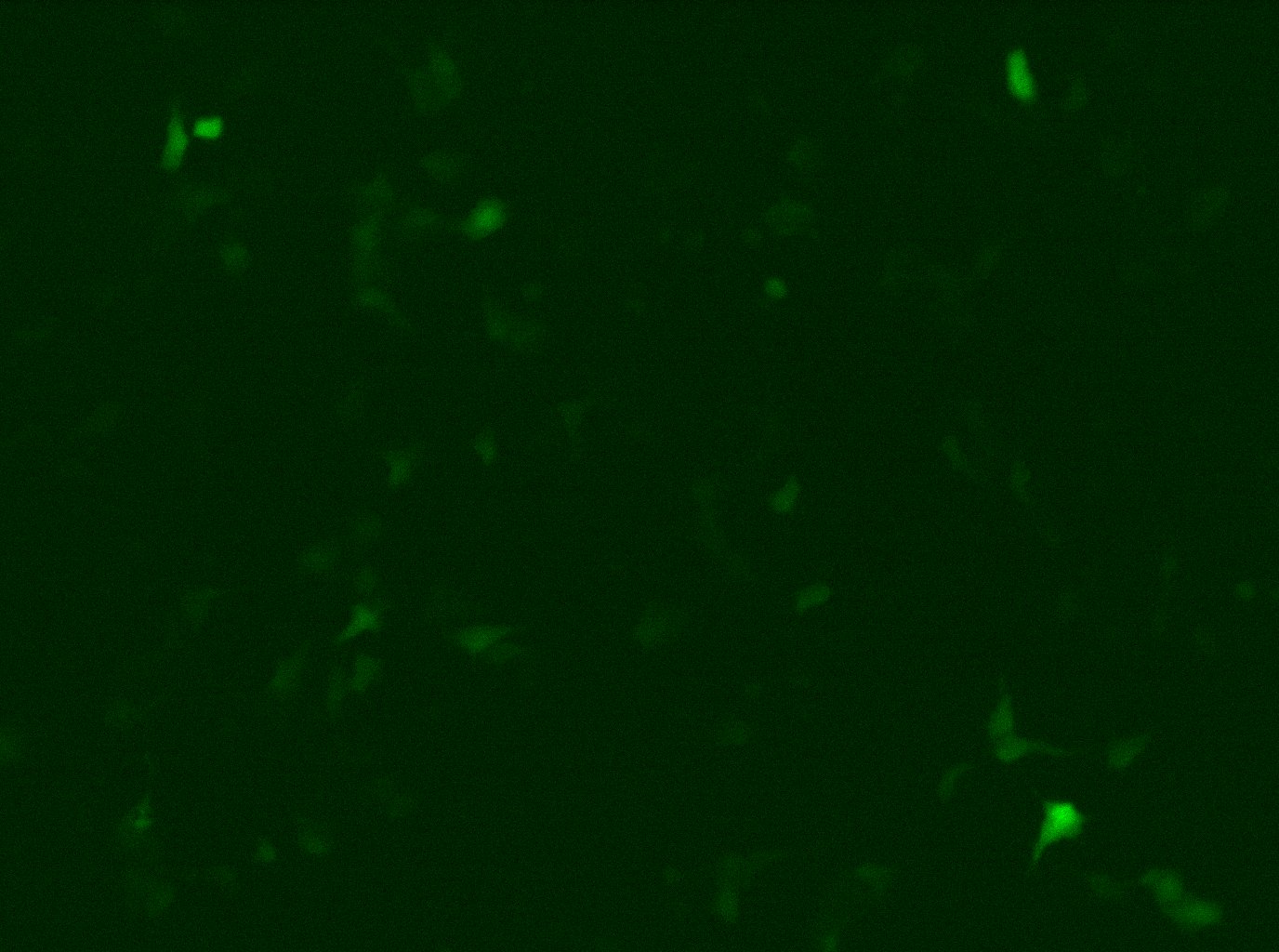

Supplement: Supplementary file 1 [file DataSheet3.ZIP › Sup Fig2A microscopy images/Vehicle-eGFP.jpg]

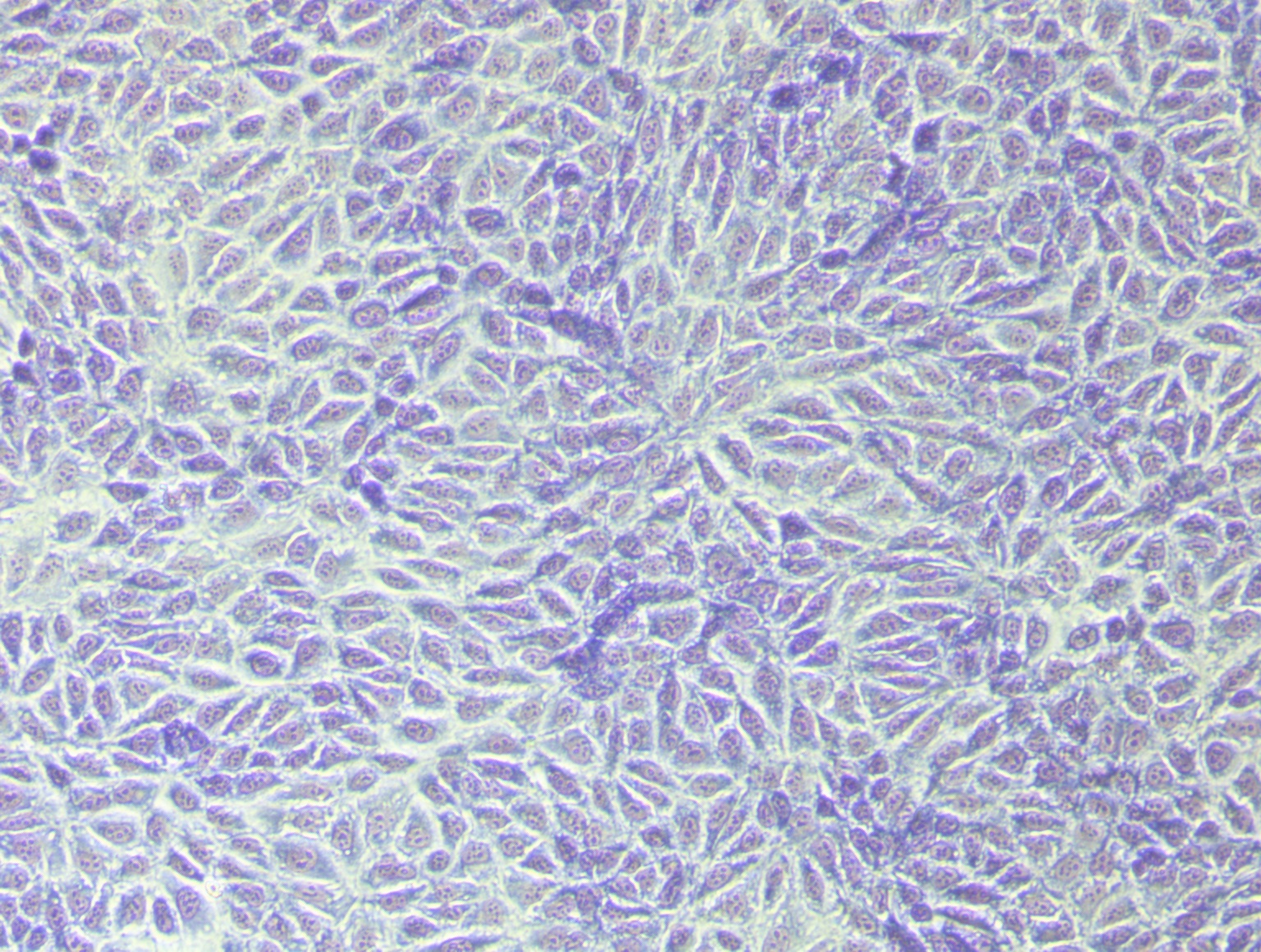

Supplement: Supplementary file 5 [file DataSheet4.ZIP › Sup Fig2B microscopy images/mock.jpg]

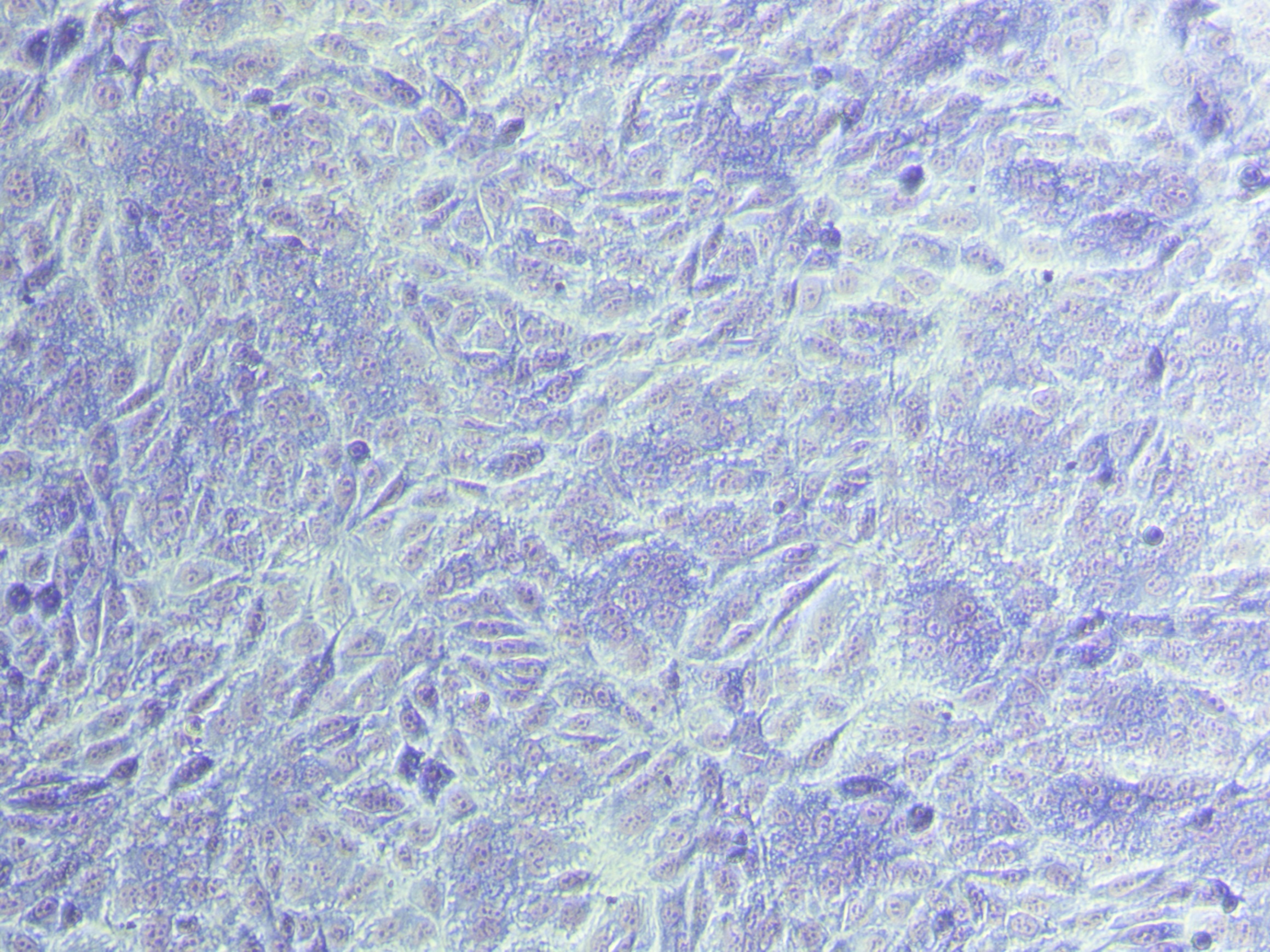

Supplement: Supplementary file 5 [file DataSheet4.ZIP › Sup Fig2B microscopy images/Toosendanin 0.5μM.jpg]

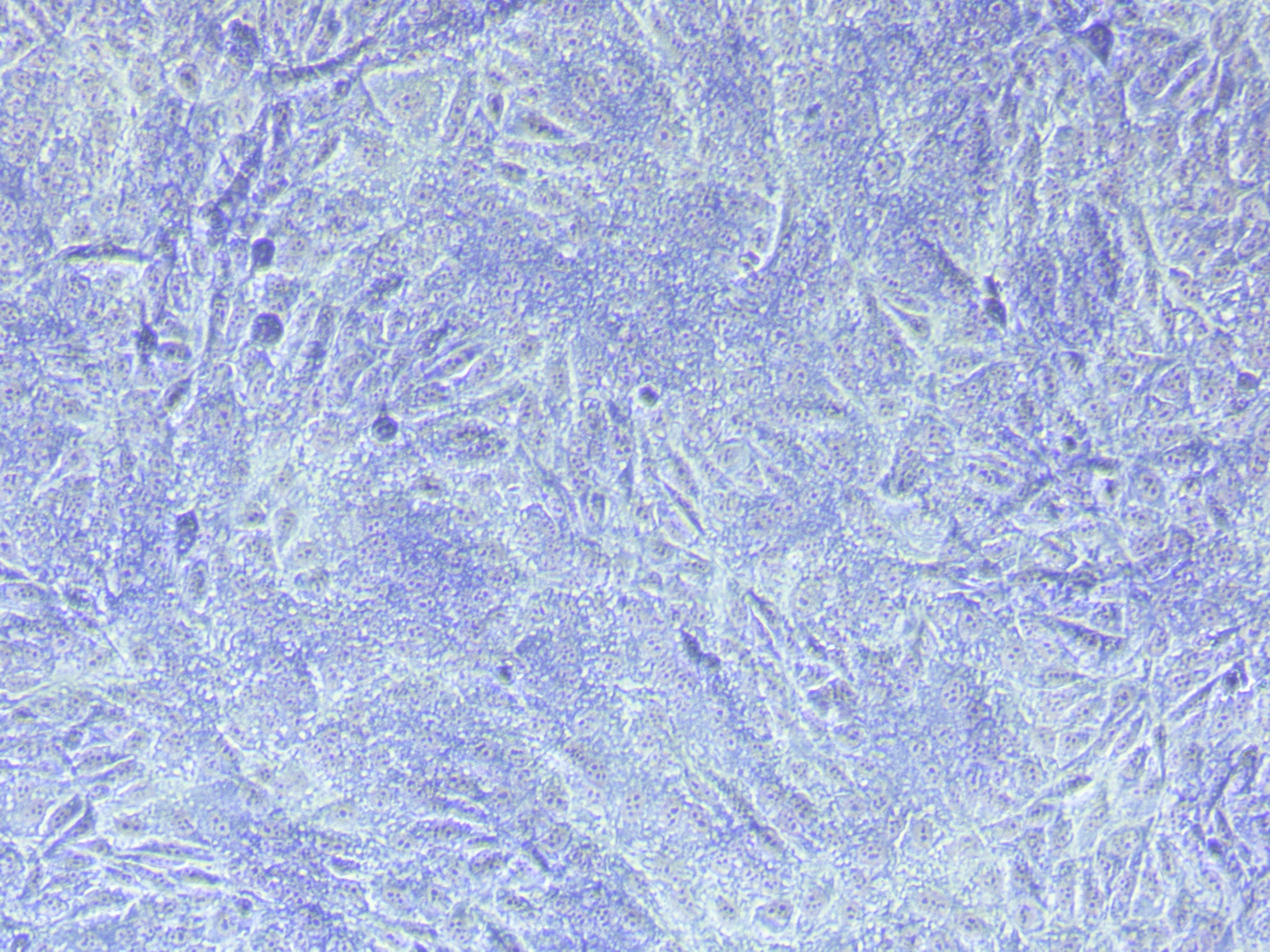

Supplement: Supplementary file 5 [file DataSheet4.ZIP › Sup Fig2B microscopy images/Toosendanin 10μM.jpg]

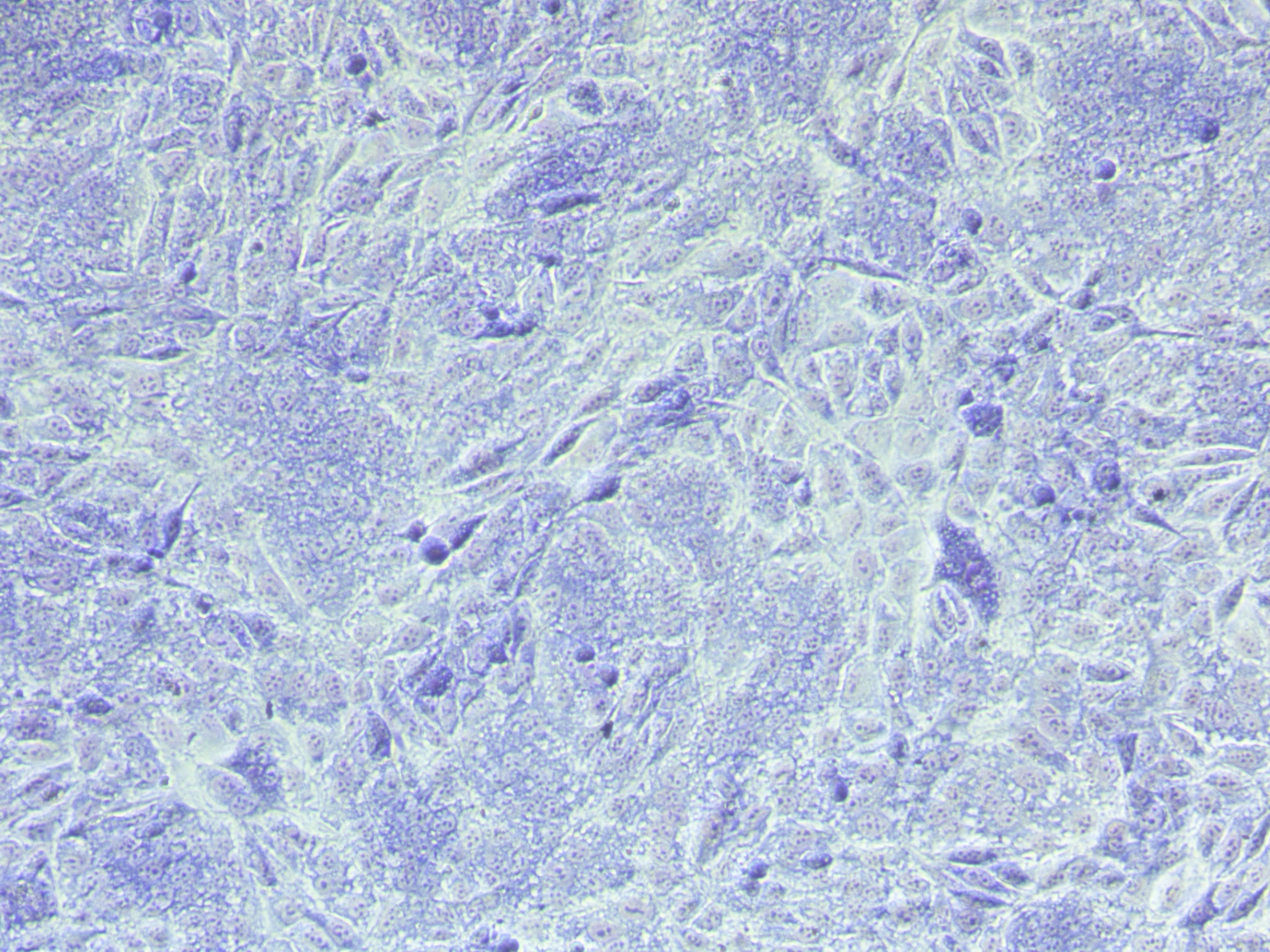

Supplement: Supplementary file 5 [file DataSheet4.ZIP › Sup Fig2B microscopy images/Toosendanin 1μM.jpg]

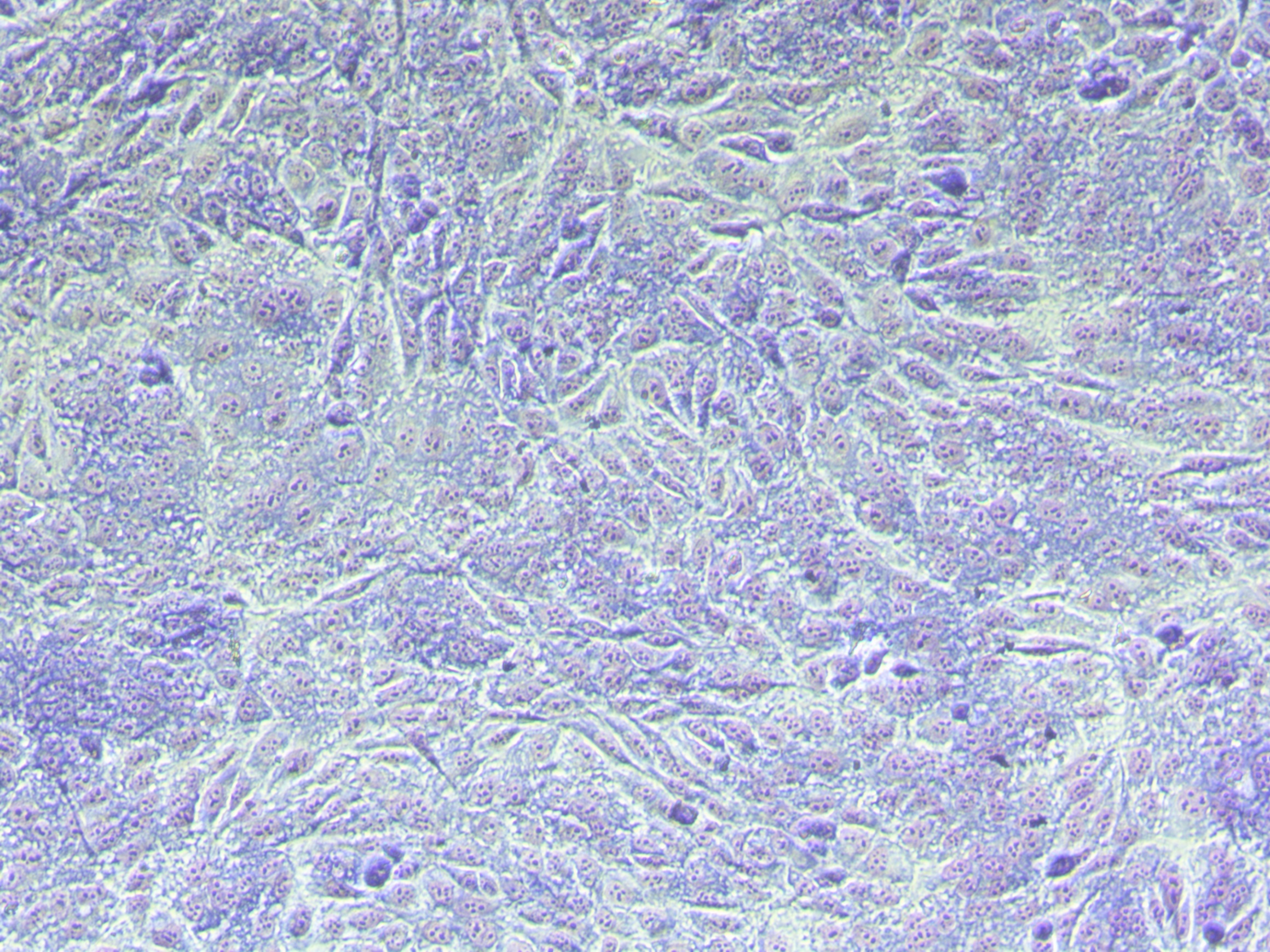

Supplement: Supplementary file 5 [file DataSheet4.ZIP › Sup Fig2B microscopy images/Toosendanin 5μM.jpg]

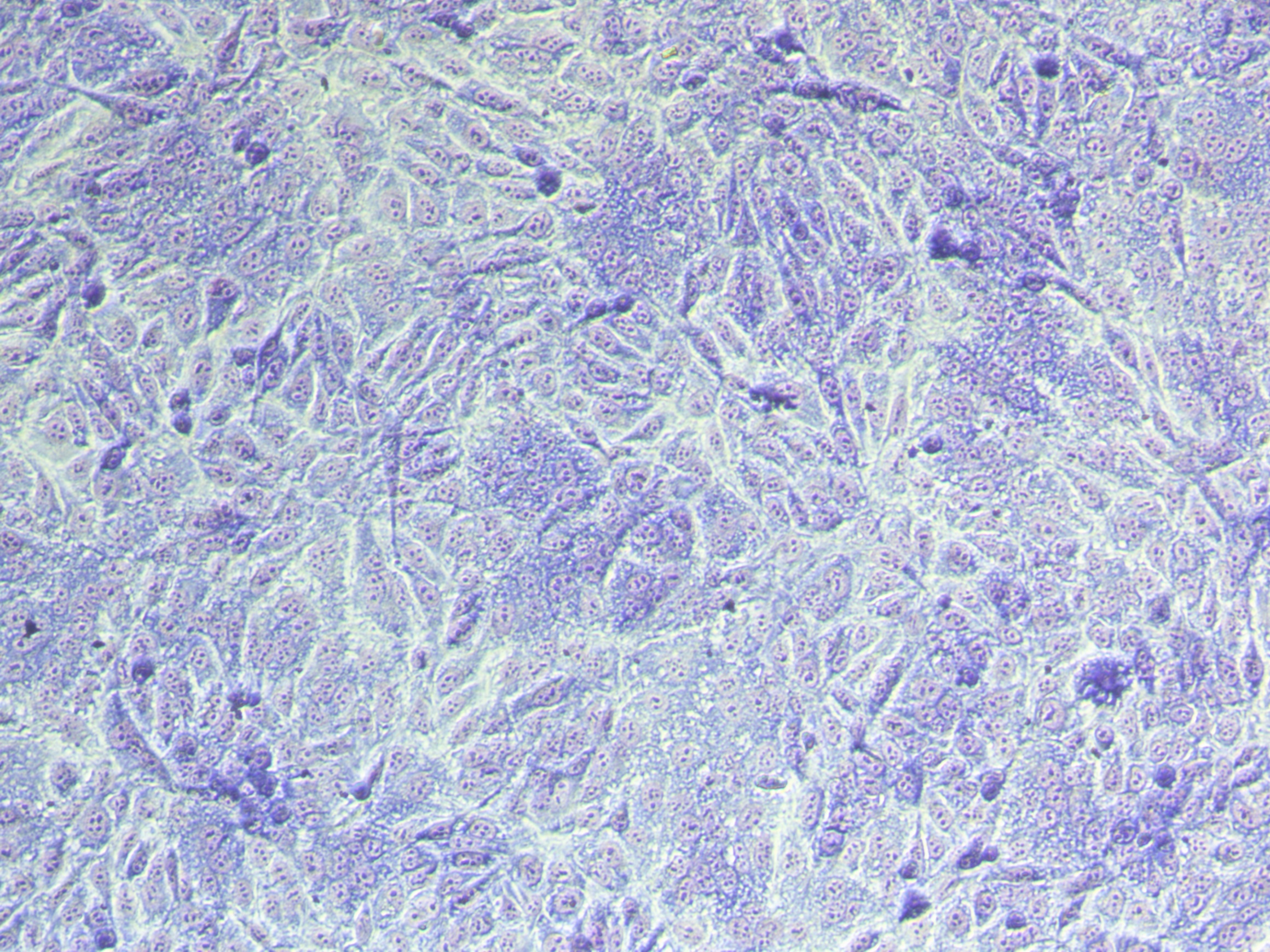

Supplement: Supplementary file 5 [file DataSheet4.ZIP › Sup Fig2B microscopy images/Vehicle.jpg]

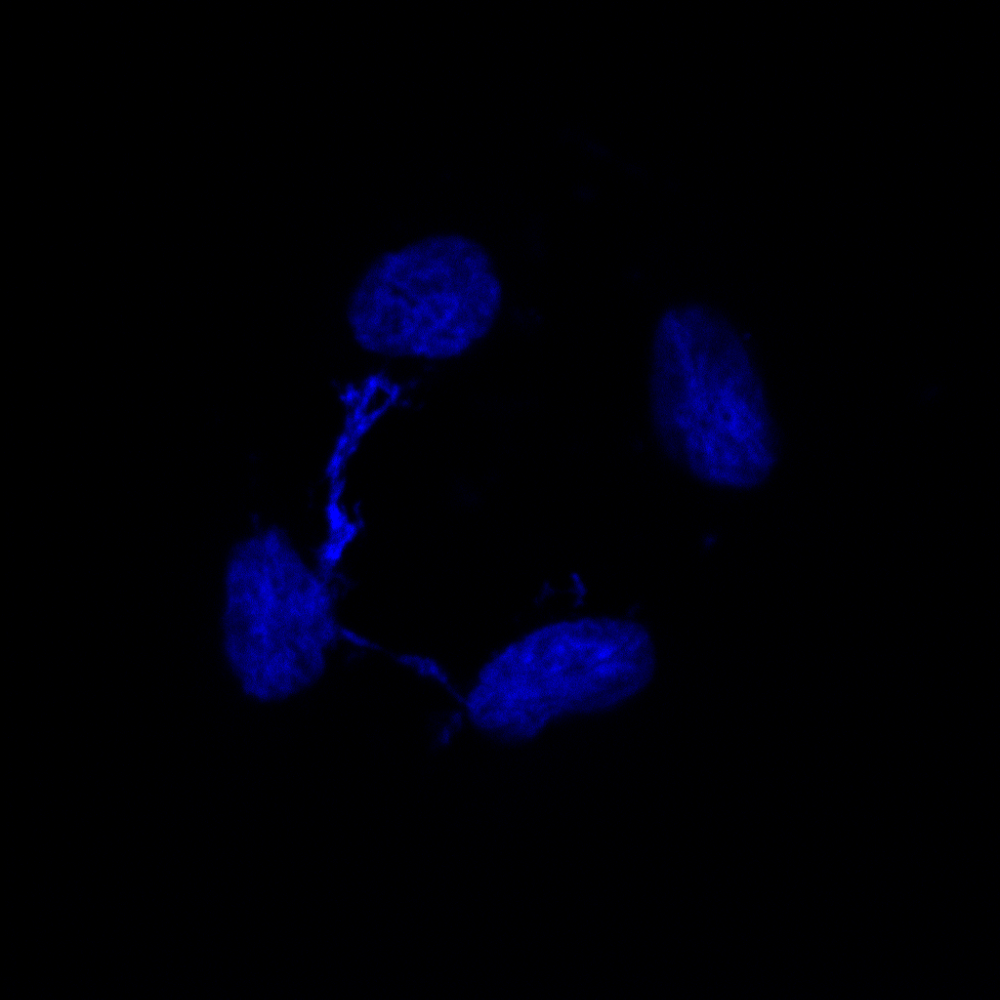

Supplement: Supplementary file 7 [file DataSheet2.ZIP › Fig4D microscopy images/Toosendanin-DAPI.tif]

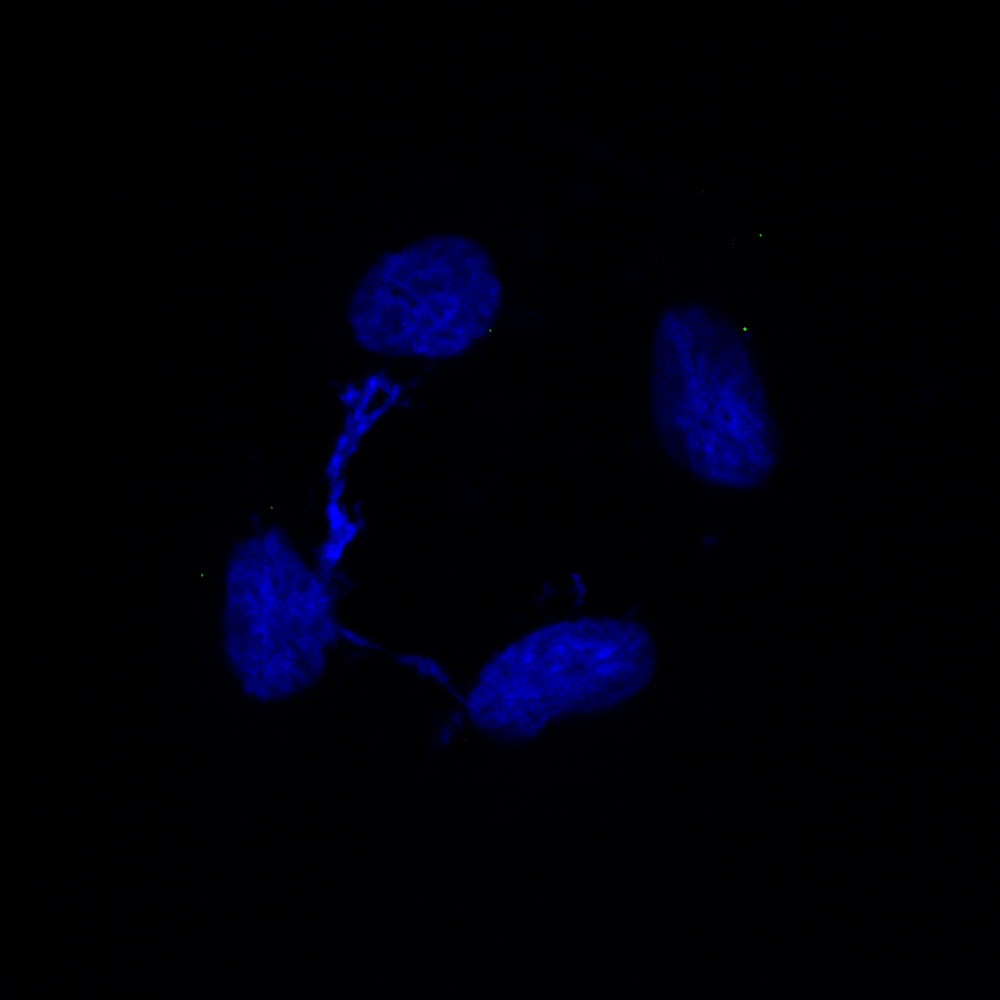

Supplement: Supplementary file 7 [file DataSheet2.ZIP › Fig4D microscopy images/Toosendanin-Merge.tif]

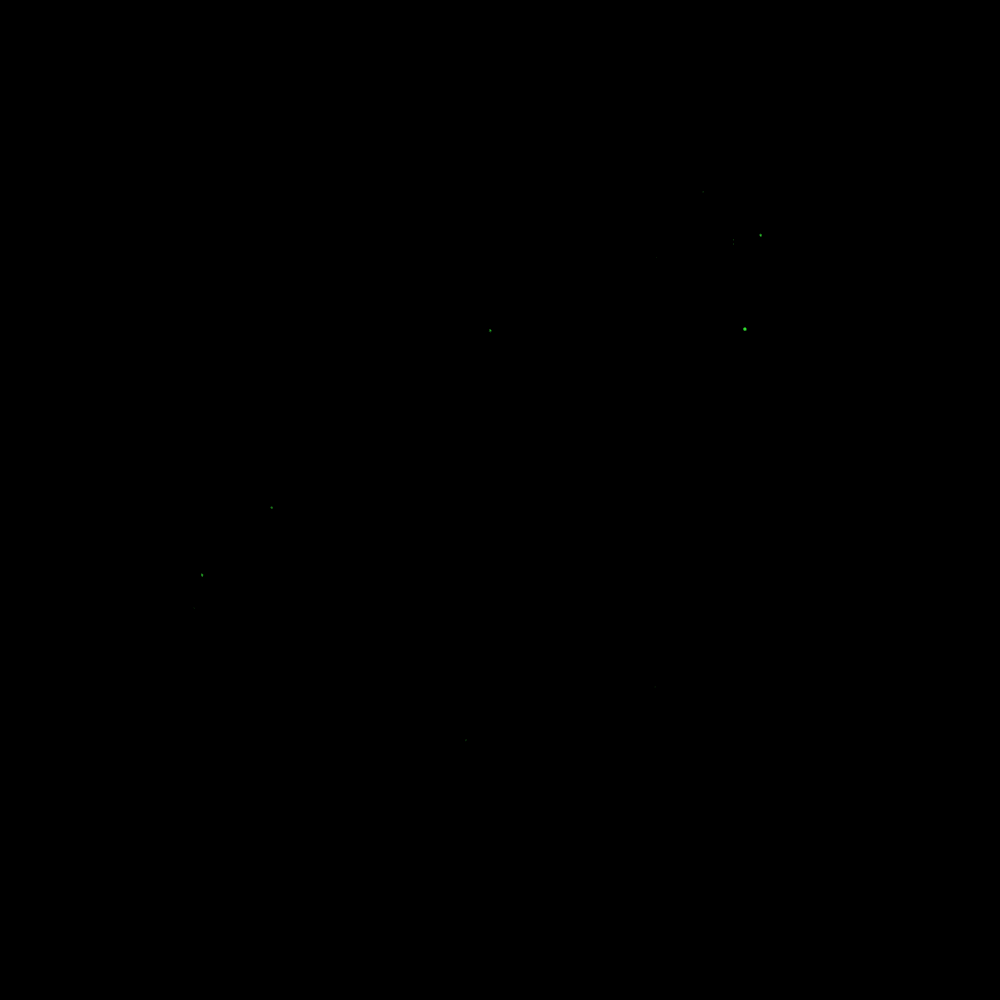

Supplement: Supplementary file 7 [file DataSheet2.ZIP › Fig4D microscopy images/Toosendanin-SF-NP.tif]

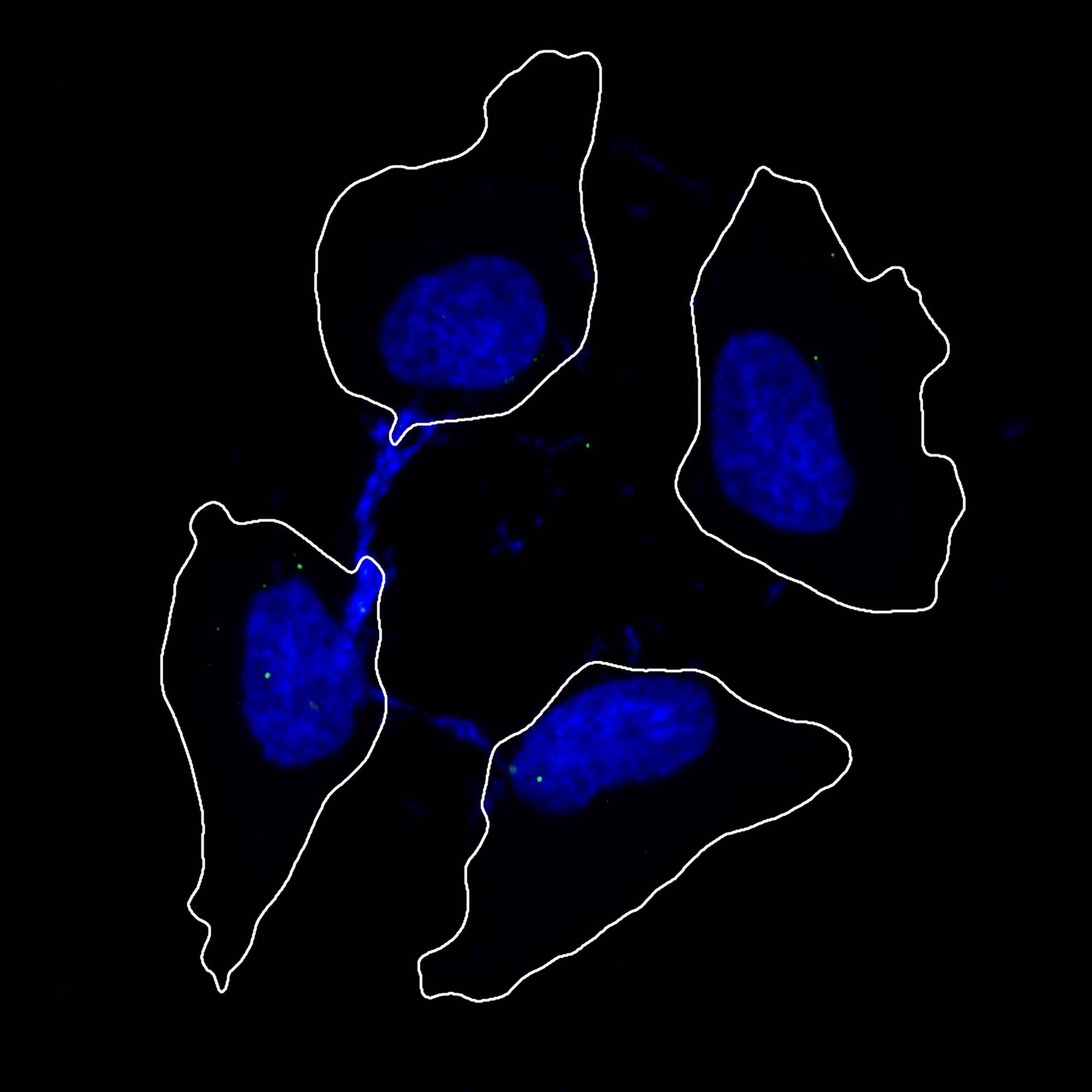

Supplement: Supplementary file 7 [file DataSheet2.ZIP › Fig4D microscopy images/Toosendanin.tif]

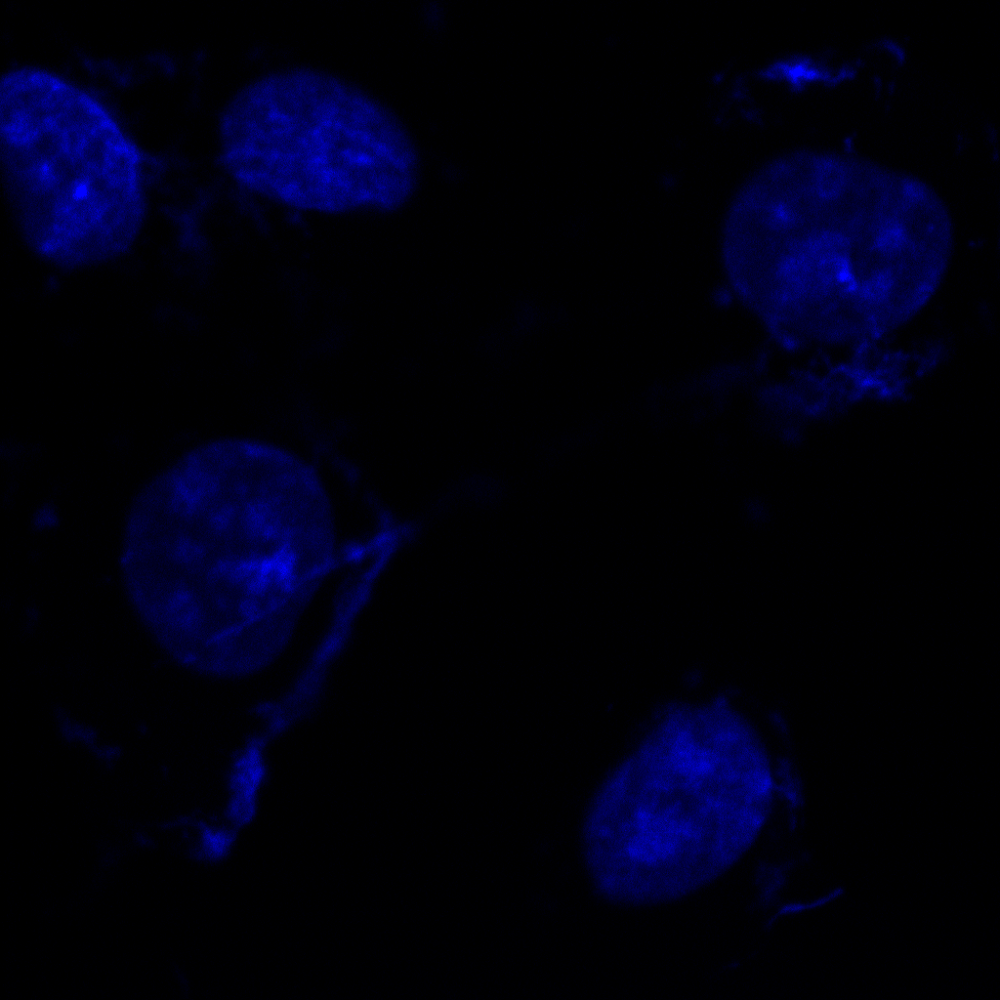

Supplement: Supplementary file 7 [file DataSheet2.ZIP › Fig4D microscopy images/Vehicle-DAPI.tif]

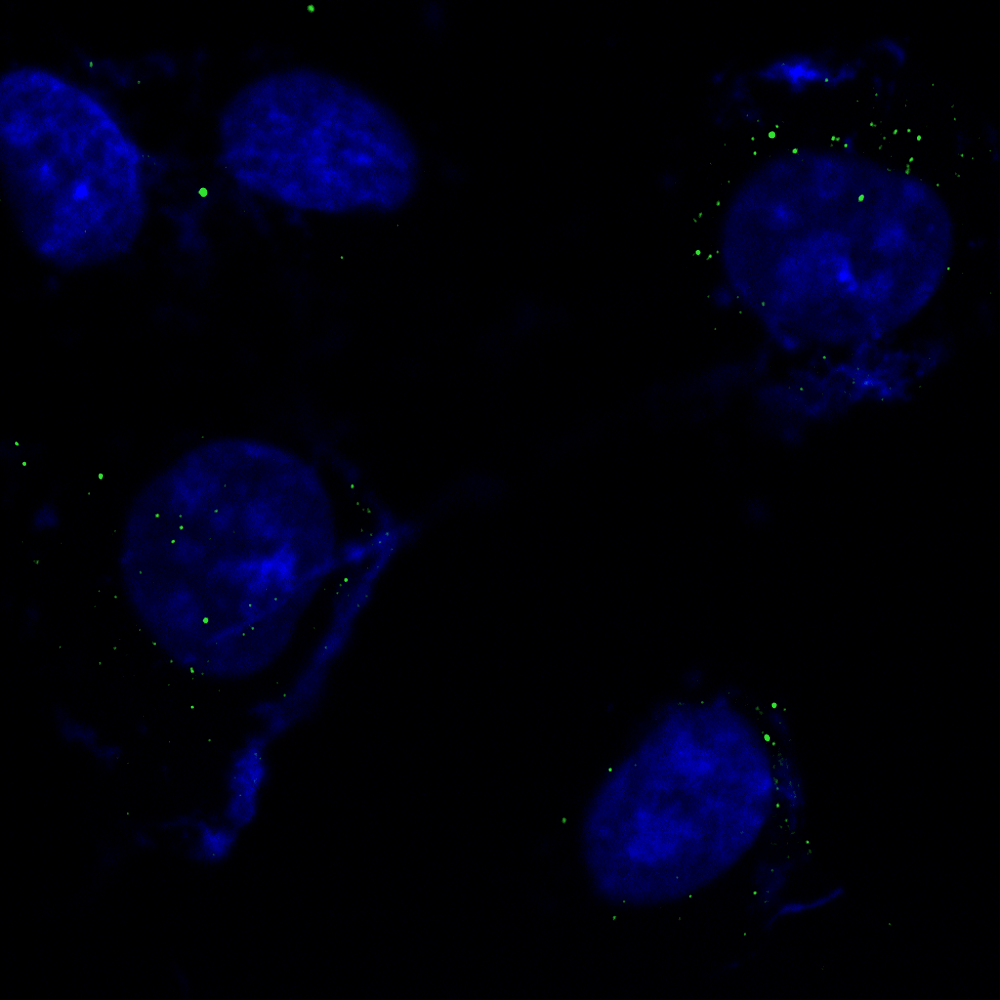

Supplement: Supplementary file 7 [file DataSheet2.ZIP › Fig4D microscopy images/Vehicle-Merge.tif]

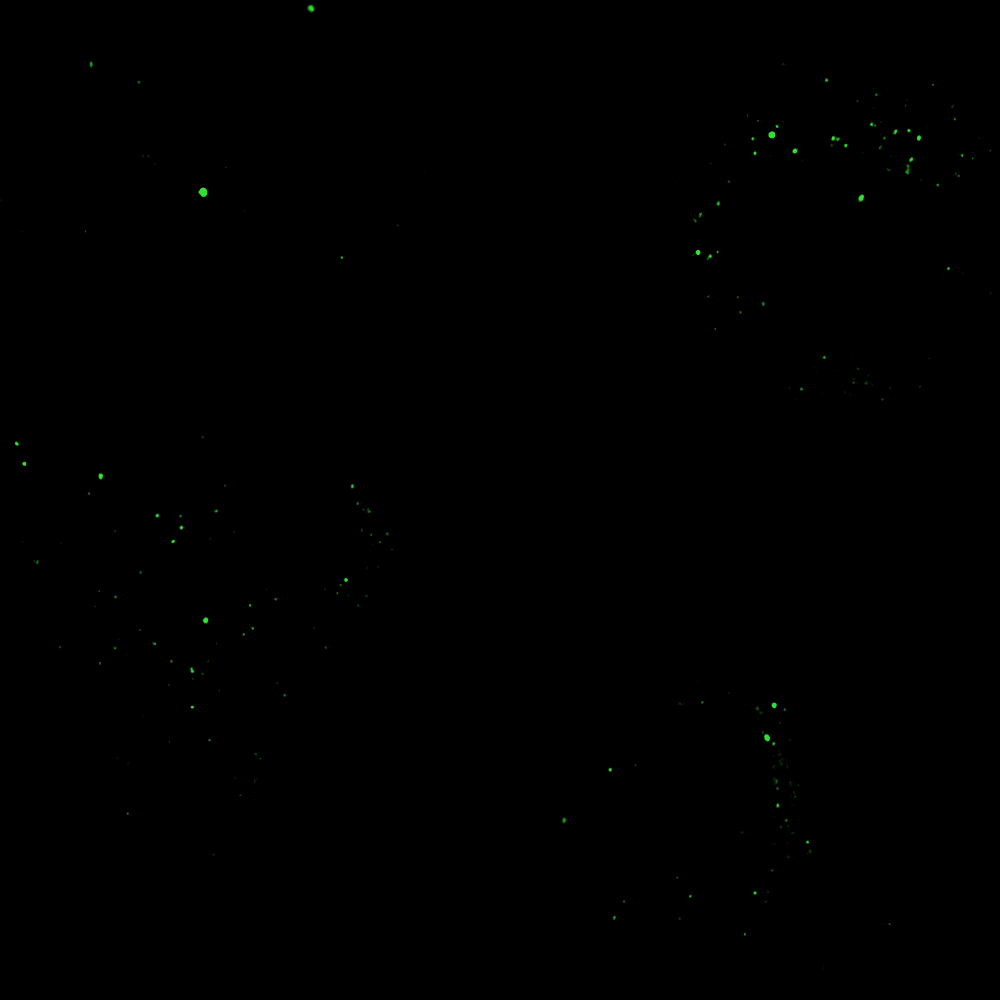

Supplement: Supplementary file 7 [file DataSheet2.ZIP › Fig4D microscopy images/Vehicle-SF-NP.tif]

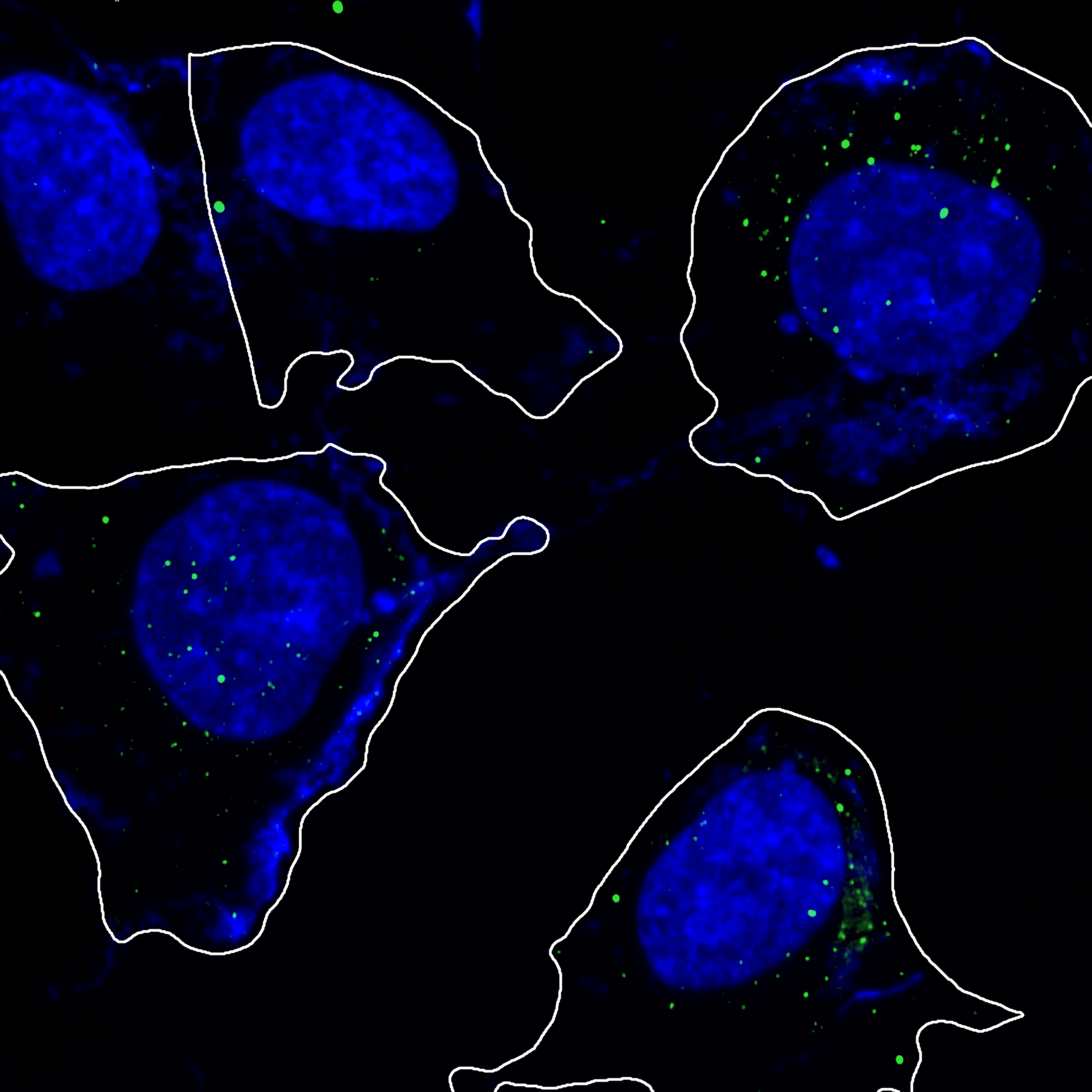

Supplement: Supplementary file 7 [file DataSheet2.ZIP › Fig4D microscopy images/Vehicle.tif]

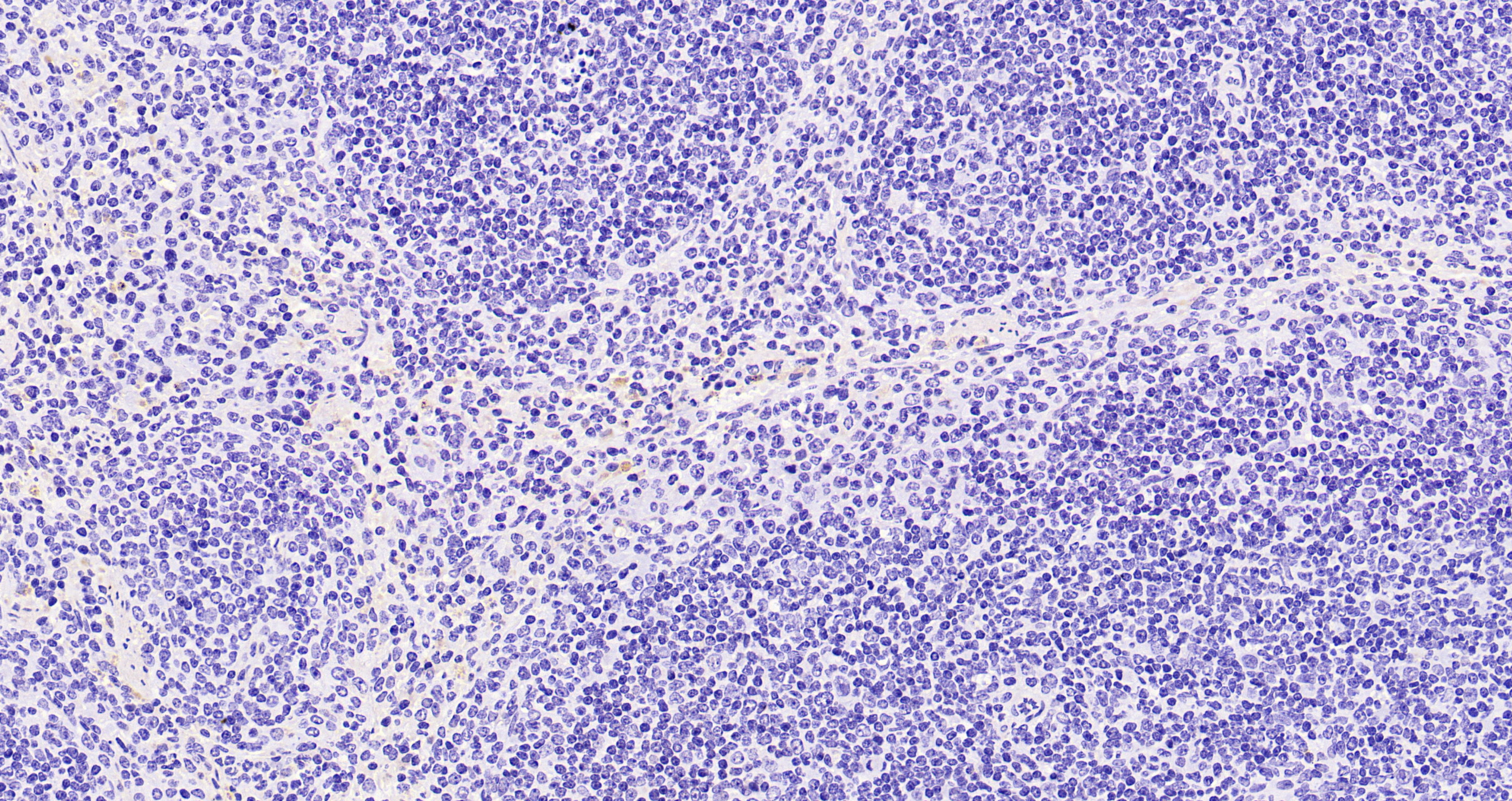

Supplement: Supplementary file 8 [file DataSheet5.ZIP › Fig5 IHC and HE staining images/Fig5B Toosendanin.jpg]

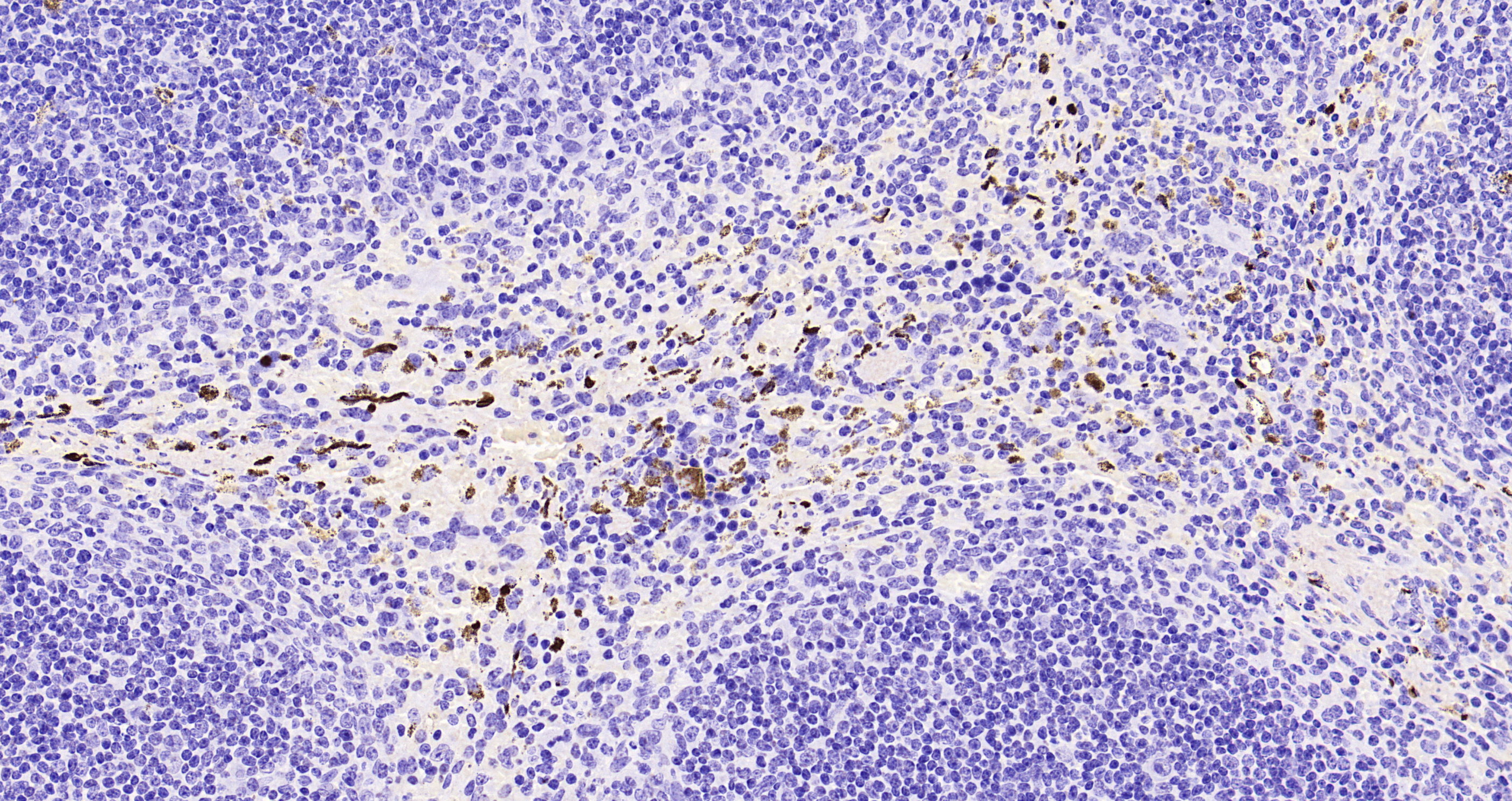

Supplement: Supplementary file 8 [file DataSheet5.ZIP › Fig5 IHC and HE staining images/Fig5B Vehicle.jpg]

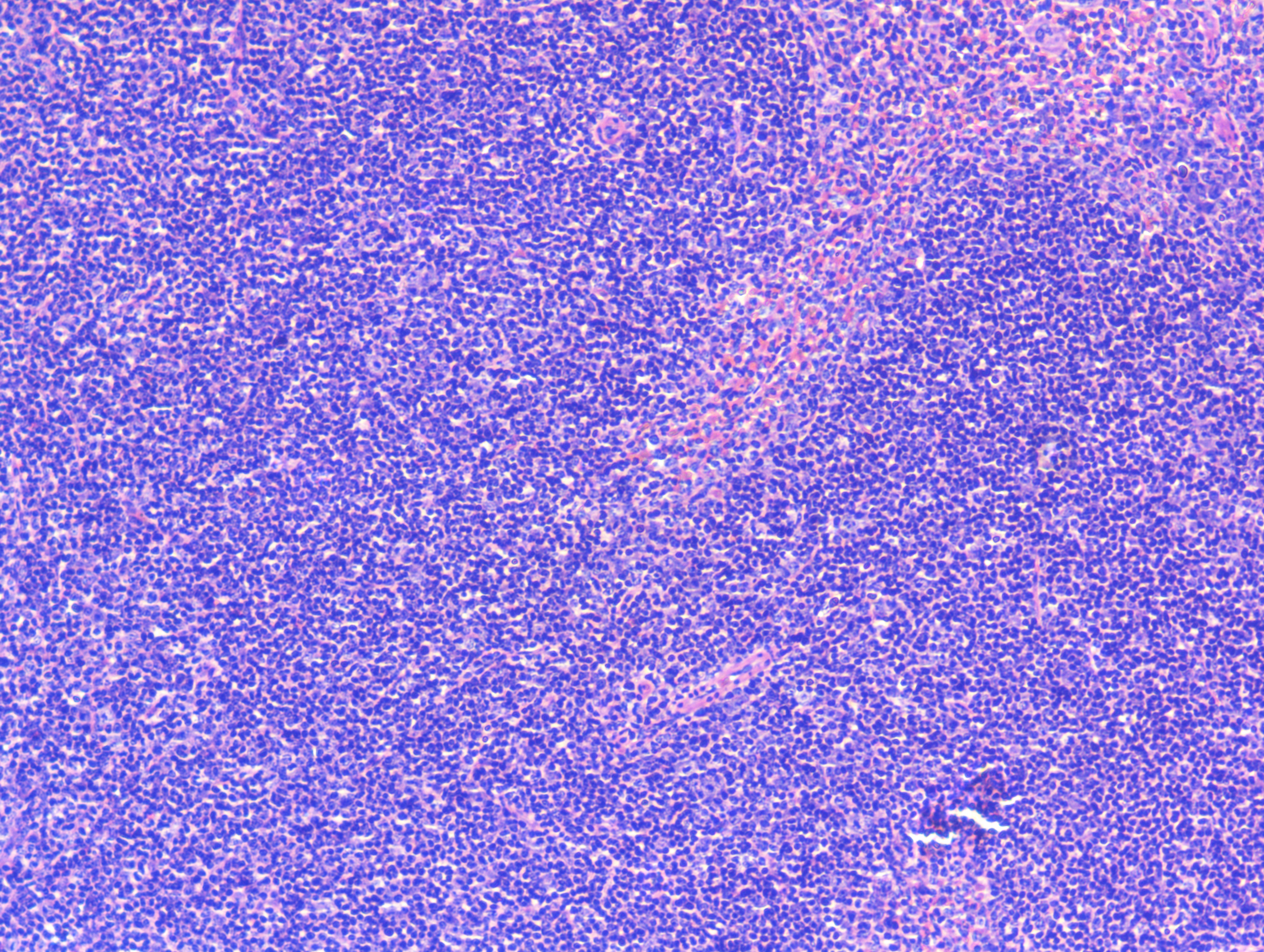

Supplement: Supplementary file 8 [file DataSheet5.ZIP › Fig5 IHC and HE staining images/Fig5C Toosendanin.jpg]

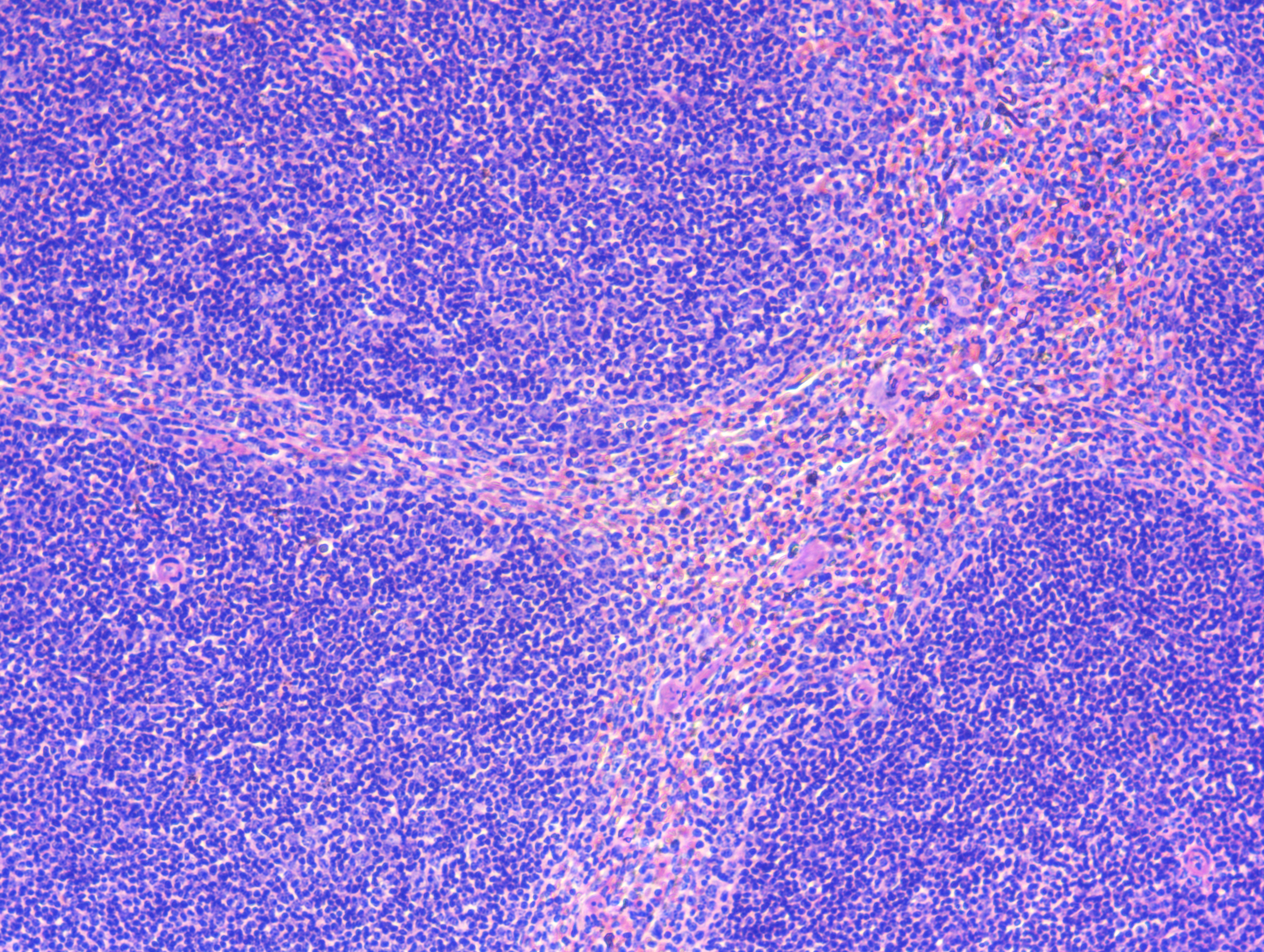

Supplement: Supplementary file 8 [file DataSheet5.ZIP › Fig5 IHC and HE staining images/Fig5C Vehicle.jpg]

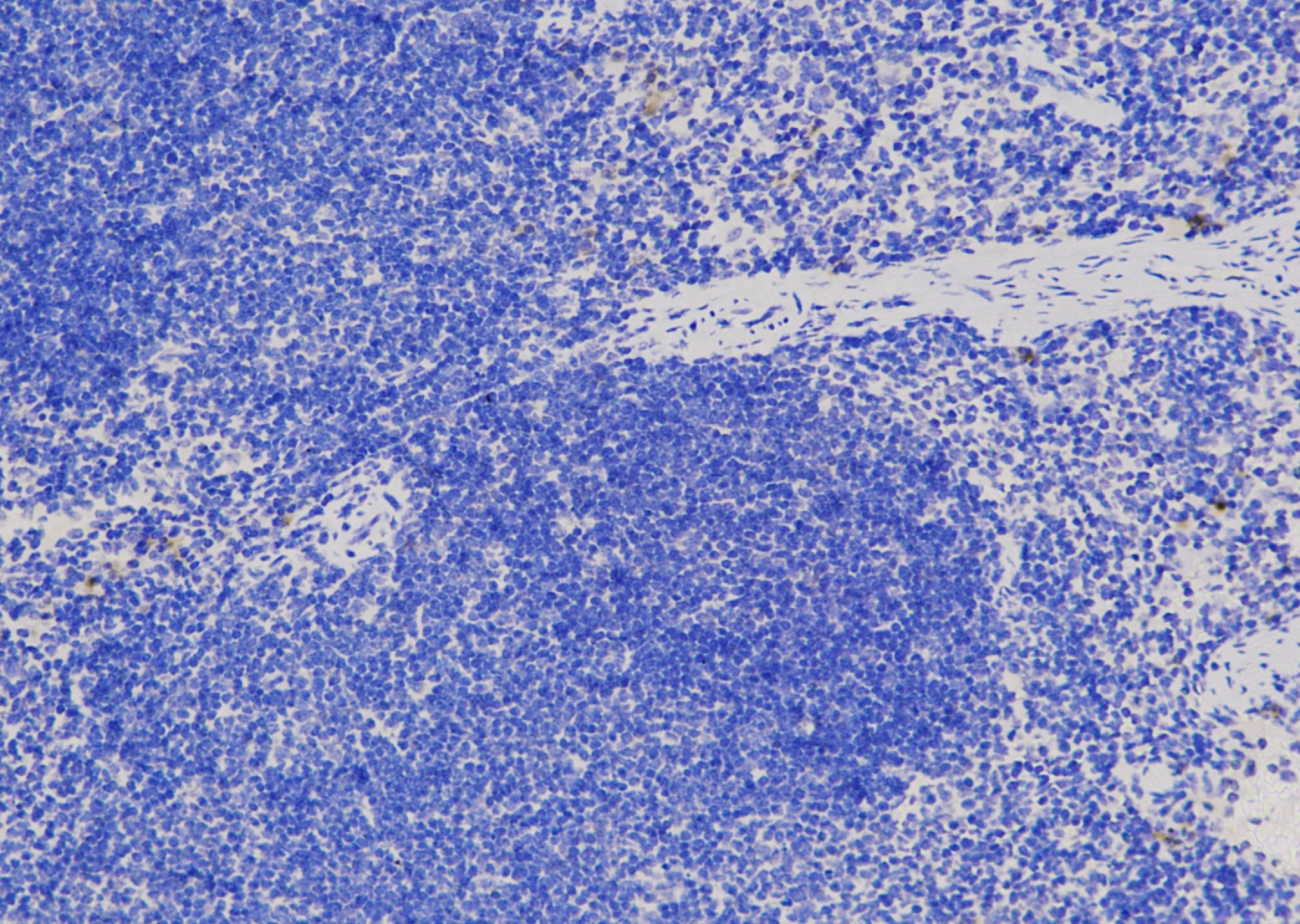

Supplement: Supplementary file 8 [file DataSheet5.ZIP › Fig5 IHC and HE staining images/Fig5E Toosendanin.jpg]

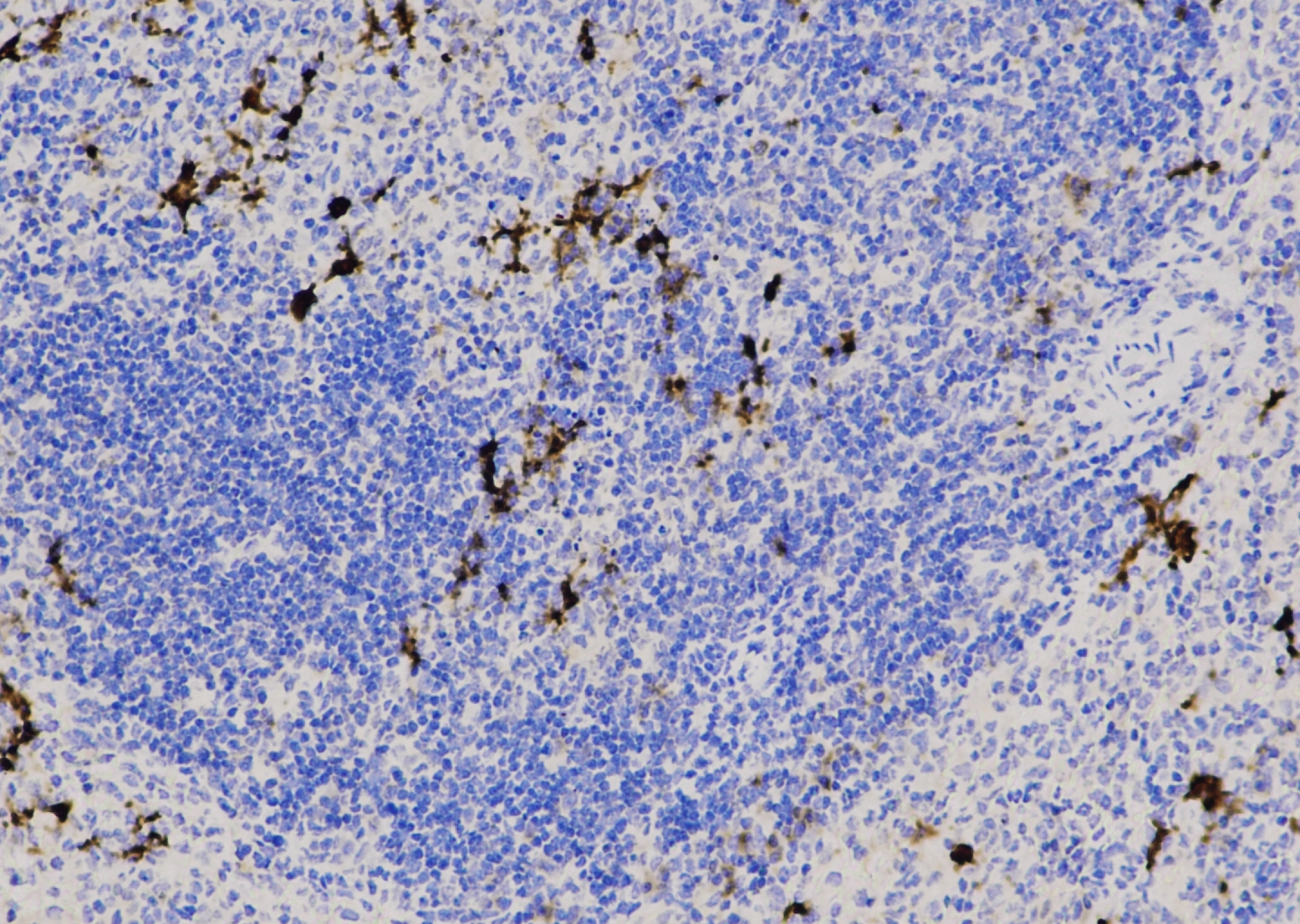

Supplement: Supplementary file 8 [file DataSheet5.ZIP › Fig5 IHC and HE staining images/Fig5E Vehicle.jpg]
