# Supplementary material for: Screening of a Small Molecule Compound Library Identifies Toosendanin as an Inhibitor Against Bunyavirus and SARS-CoV-2
Source: Front Pharmacol. 2021 Nov 11;12:735223. doi: 10.3389/fphar.2021.735223 (PMC8632254; doi:10.3389/fphar.2021.735223)
Supplement: Supplementary file 2 [file DataSheet7.docx]

**Supplementary Materials**

Table 1. The primer sequences used in qRT-PCR.

| Primer | Sequence 5’-3’ |
| --- | --- |
| SFTSV-NP-for | ATGTCAGAGTGGTCCAGGA |
| SFTSV-NP-rev | TCTCCACCTGTCTCCTTCAG |
| RVFV-NP-for | TAAGGGCGATATTGGATGCT |
| RVFV-NP-rev | TTGCAGCAACTTCCTCCTTT |
| SARS-CoV-2-RBD -for | CAATGGTTTAACAGGCACAGG |
| SARS-CoV-2-RBD -rev | CTCAAGTGTCTGTGGATCACG |
| GAPDH-for | CTCTGCTCCTCCTGTTCGAC |
| GAPDH-rev | AATCCGTTGACTCCGACCTT |
| 18S rRNA-for | TGAGAAACGGCTACCACATC |
| 18S rRNA-rev | TTACAGGGCCTCGAAAGAGT |

**
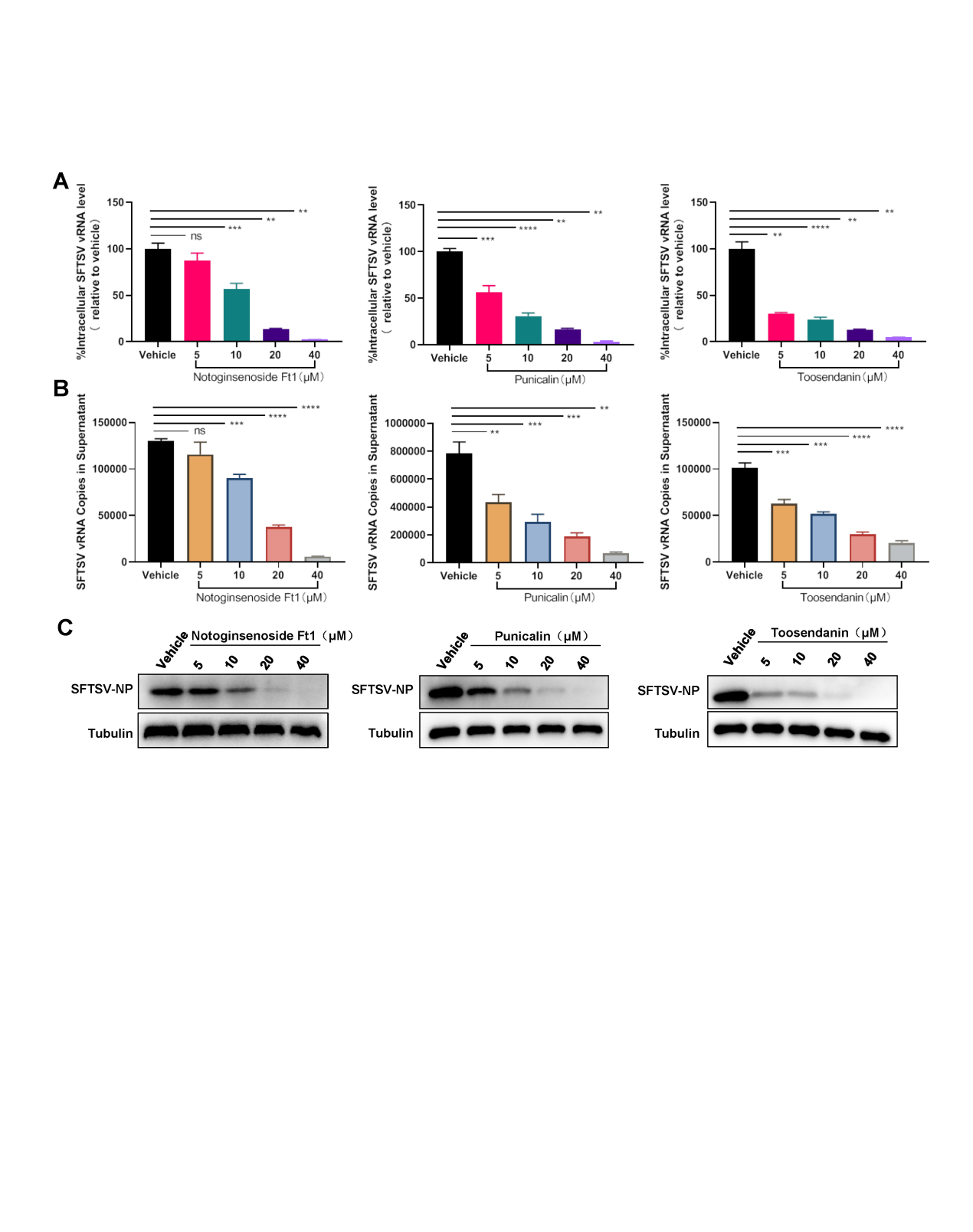
**

**Sup. Fig 1. Validation of the anti-viral activity of the hit compounds on Vero cells.** Vero cells pre-treated with different concentrations of drugs were infected with SFTSV at an MOI of 0.125. At 24 hpi, cells and supernatants were harvested for intracellular (A) and extracellular viral RNA (B) determination by qRT-PCR, and nucleoprotein expression detection by western blot (C).

Experiments were performed in triplicates (n=3). Data shown are means ± SD. Comparison of mean values (A,B) between two groups were analyzed by Student’s t test. **p < 0.01; ***p < 0.001; ****p < 0.0001; ns, no significance.

**
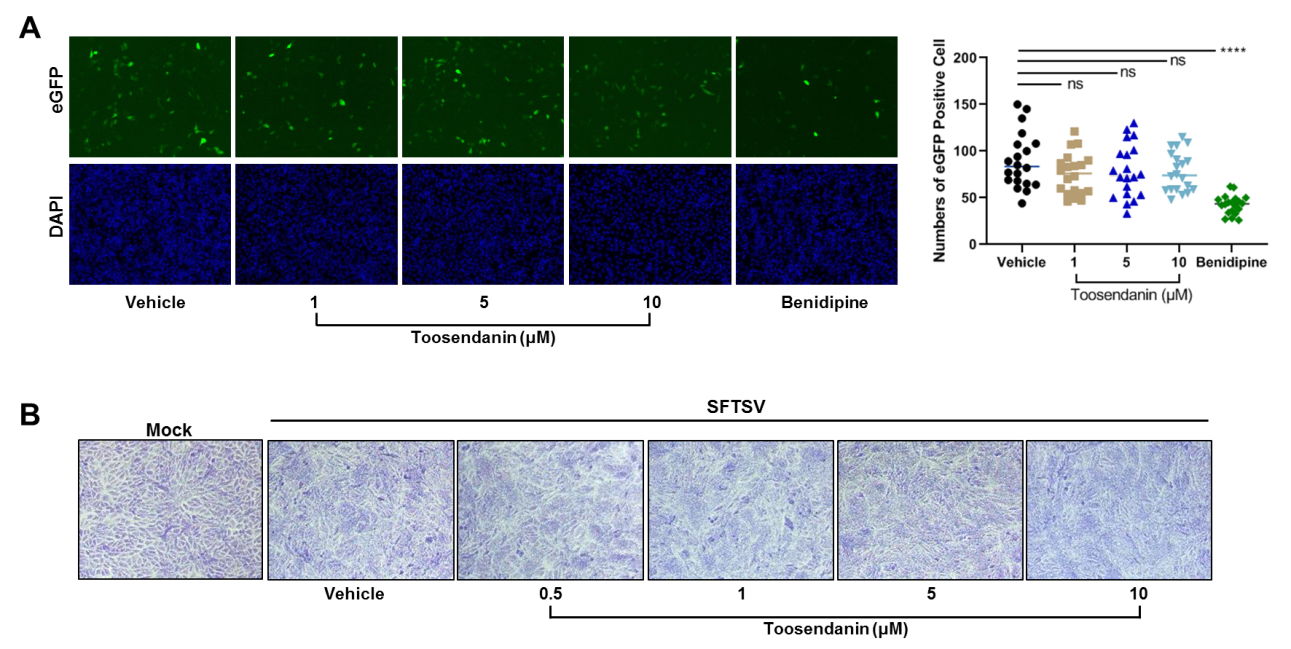
**

**Sup. Fig 2. Effect of toosendanin on SFTSV mini-genome activity and syncytium formation.** (A) Effect of toosendanin on SFTSV mini-genome activity. BSRT7 cells transfected with SFTSV mini-genome were treated with indicated concentrations of toosendanin or vehicle (DMSO). The eGFP signal was captured and analyzed. Median values of each group were indicated by lines. Comparisons of median value between two groups were performed by Mann-Whitney test. (B) Effect of toosendanin on SFTSV syncytium formation. HUVEC cells were infected with SFTSV (MOI=5) for 24 h and treated with indicated concentrations of toosendanin or vehicle (DMSO) for 1 h, followed by incubation of low pH buffer (pH 5.0) for 20 min. Cells were fixed by methanol and stained by Giemsa solution. Syncytium formation was imaged.

Comparison of median values (A) between two groups were analyzed by Mann-Whitney test. ****p < 0.0001; ns, no significance.

**Sup. Fig 3. Weight change of SFTSV infected mice under treatment of toosendanin.** C57BL/6 were pretreated with toosendanin for 3 days and infected intraperitoneally with SFTSV (10^5^ FFU). Toosendanin (1 mg/kg/d) or vehicle was continuously delivered for 3 days. Mice were weight daily.
