# Supplementary material for: Screening of a Small Molecule Compound Library Identifies Toosendanin as an Inhibitor Against Bunyavirus and SARS-CoV-2
Source: Front Pharmacol. 2021 Nov 11;12:735223. doi: 10.3389/fphar.2021.735223 (PMC8632254; doi:10.3389/fphar.2021.735223)
Supplement: Supplementary file 3 [file Presentation1.PPTX]

## Slide 1
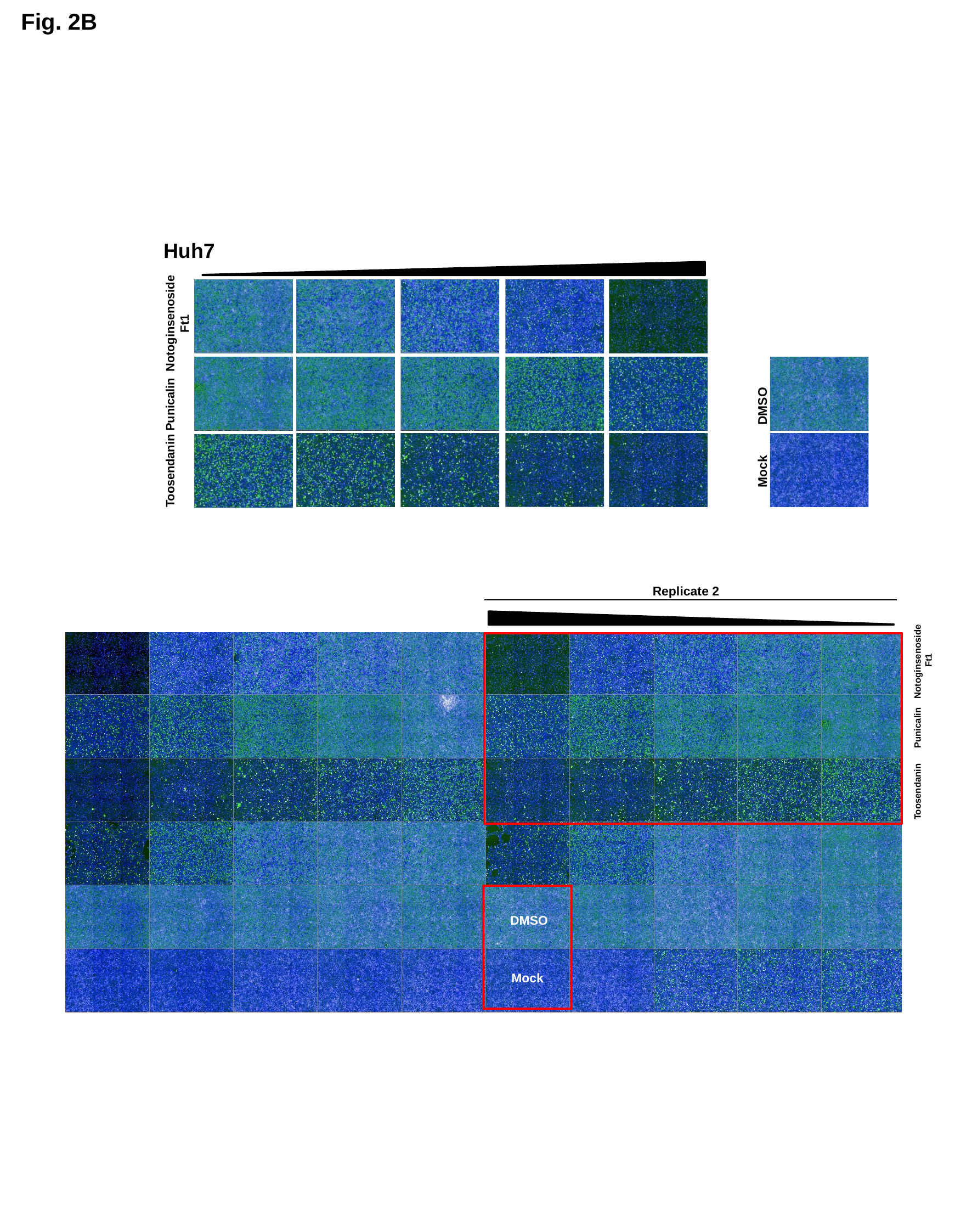

Fig. 2B
Huh7
Notoginsenoside Ft1
Punicalin
DMSO
Toosendanin
Mock
Replicate 2
Notoginsenoside
 Ft1
Punicalin
Toosendanin
DMSO
Mock

## Slide 2
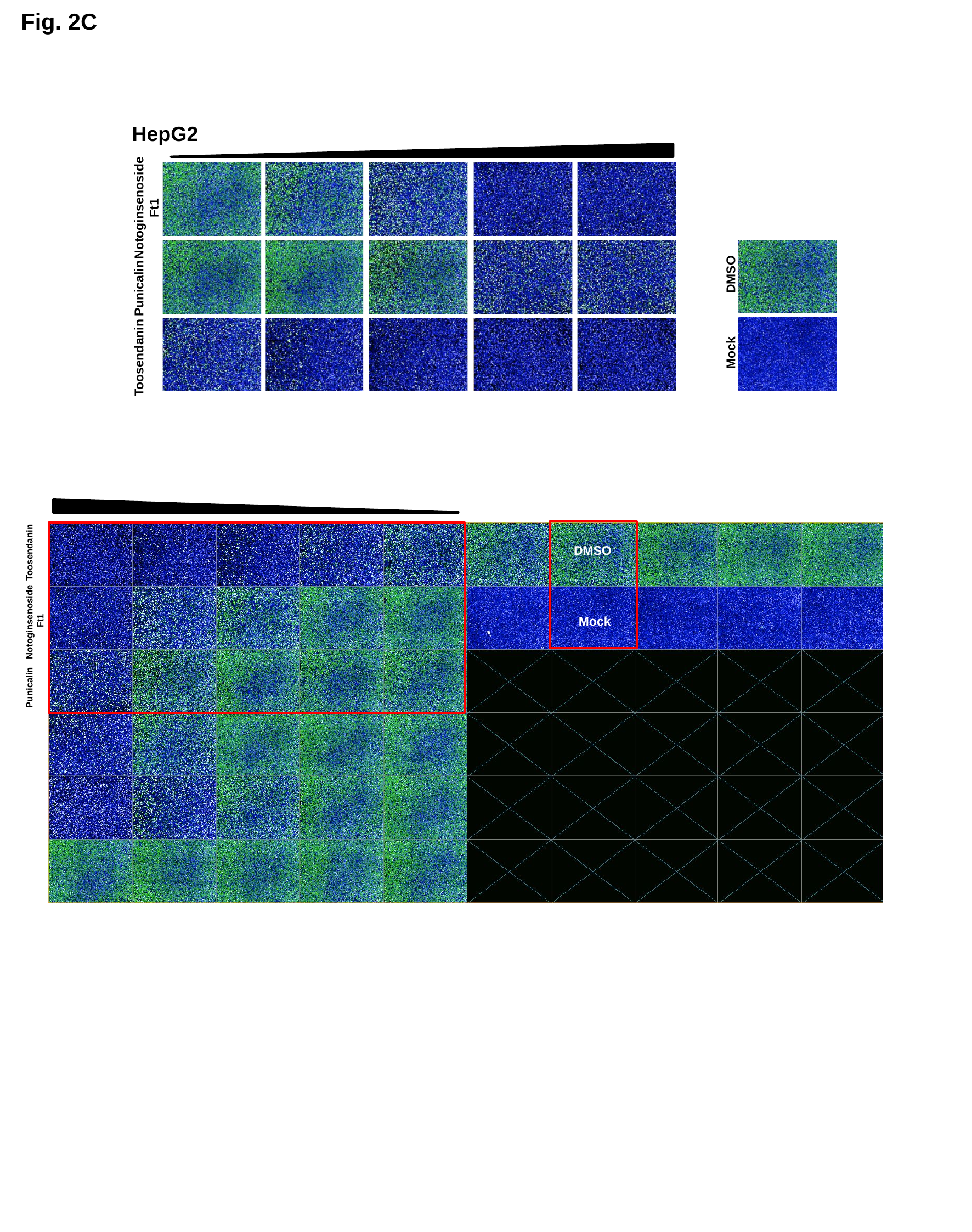

Fig. 2C
HepG2
Notoginsenoside Ft1
DMSO
Punicalin
Mock
Toosendanin
DMSO
Toosendanin
Notoginsenoside
 Ft1
Mock
Punicalin

## Slide 3
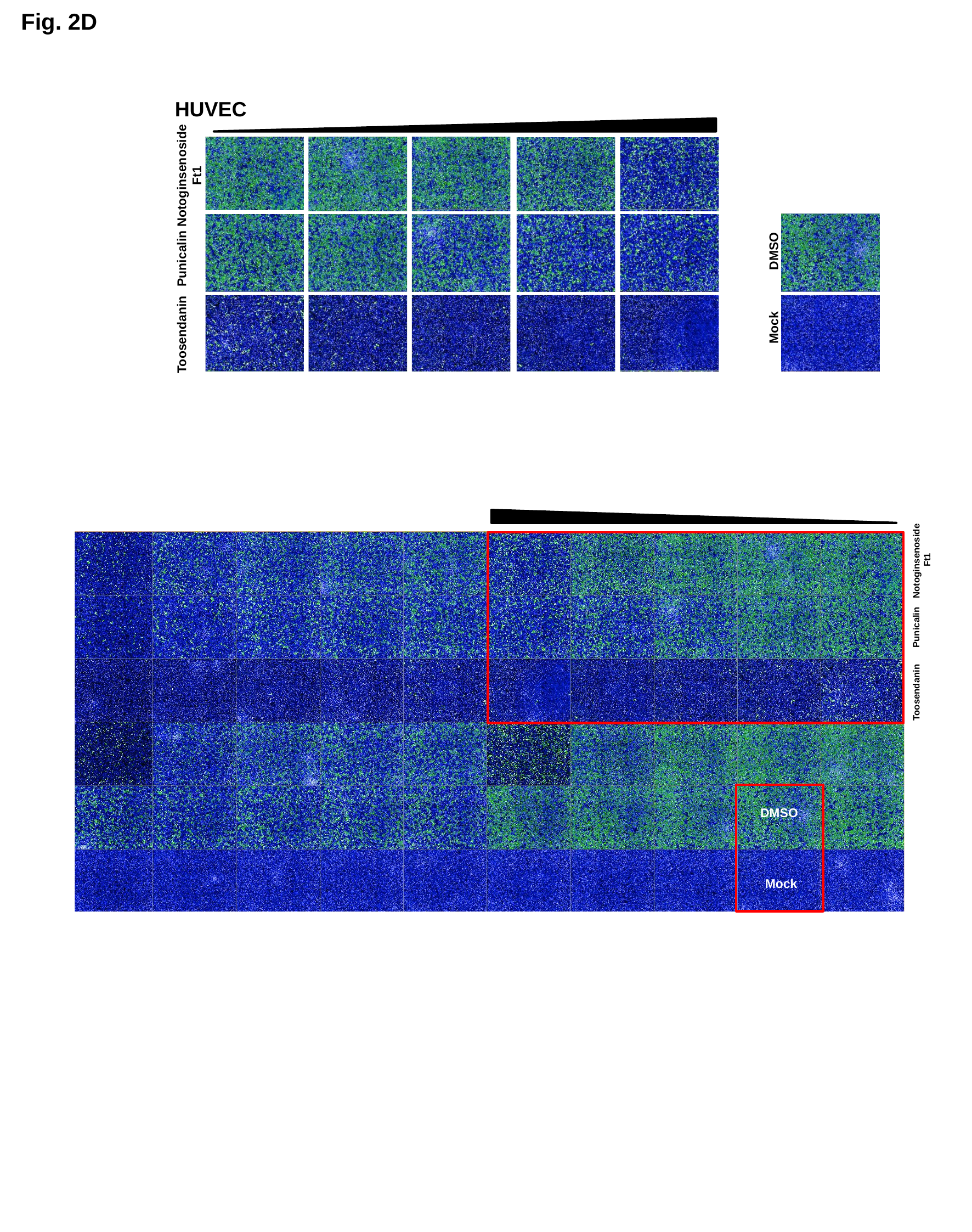

Fig. 2D
HUVEC
Notoginsenoside Ft1
DMSO
Punicalin
Mock
Toosendanin
Notoginsenoside
 Ft1
Punicalin
Toosendanin
DMSO
Mock
