# Supplementary material for: Screening of a Small Molecule Compound Library Identifies Toosendanin as an Inhibitor Against Bunyavirus and SARS-CoV-2
Source: Front Pharmacol. 2021 Nov 11;12:735223. doi: 10.3389/fphar.2021.735223 (PMC8632254; doi:10.3389/fphar.2021.735223)
Supplement: Supplementary file 6 [file DataSheet1.ZIP › Western Blot raw image data.pptx]

## Slide 1
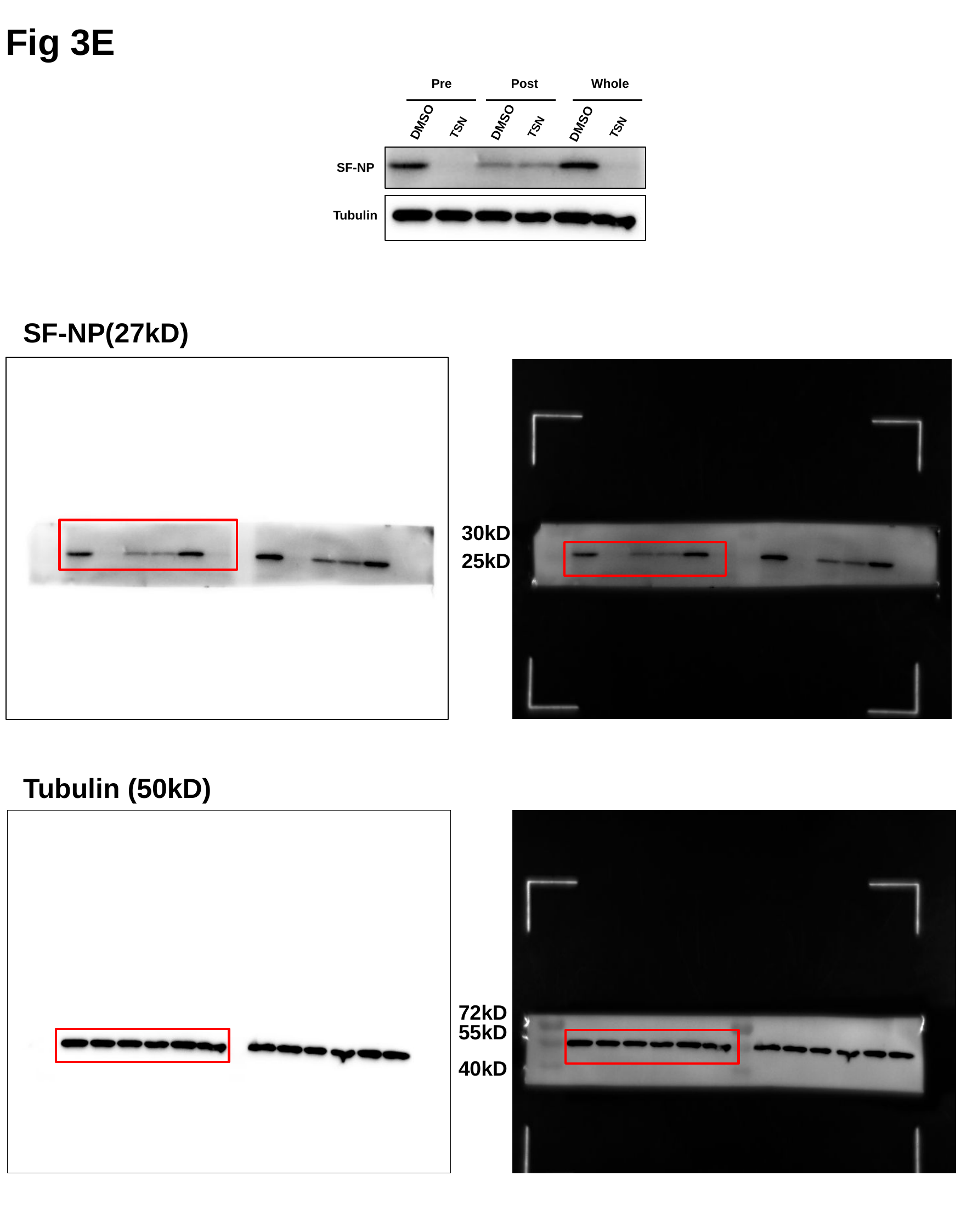

Fig 3E
Pre
Post
Whole
DMSO
DMSO
DMSO
TSN
TSN
TSN
SF-NP
SF-NP(27kD)
Tubulin
30kD
25kD
Tubulin (50kD)
72kD
55kD
40kD

## Slide 2
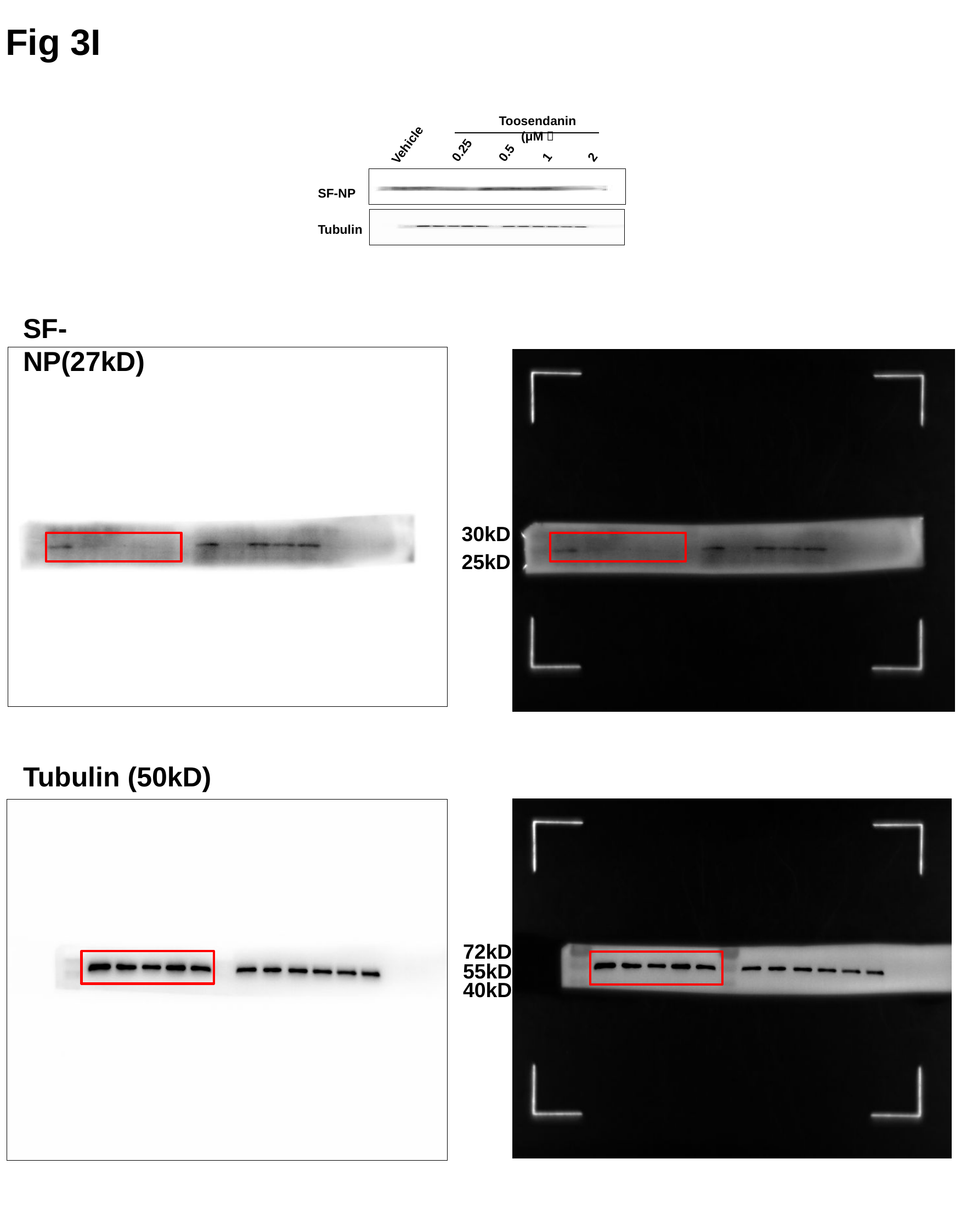

Fig 3I
Toosendanin (μM）
Vehicle
0.25
1
0.5
2
SF-NP
Tubulin
SF-NP(27kD)
30kD
25kD
Tubulin (50kD)
72kD
55kD
40kD

## Slide 3
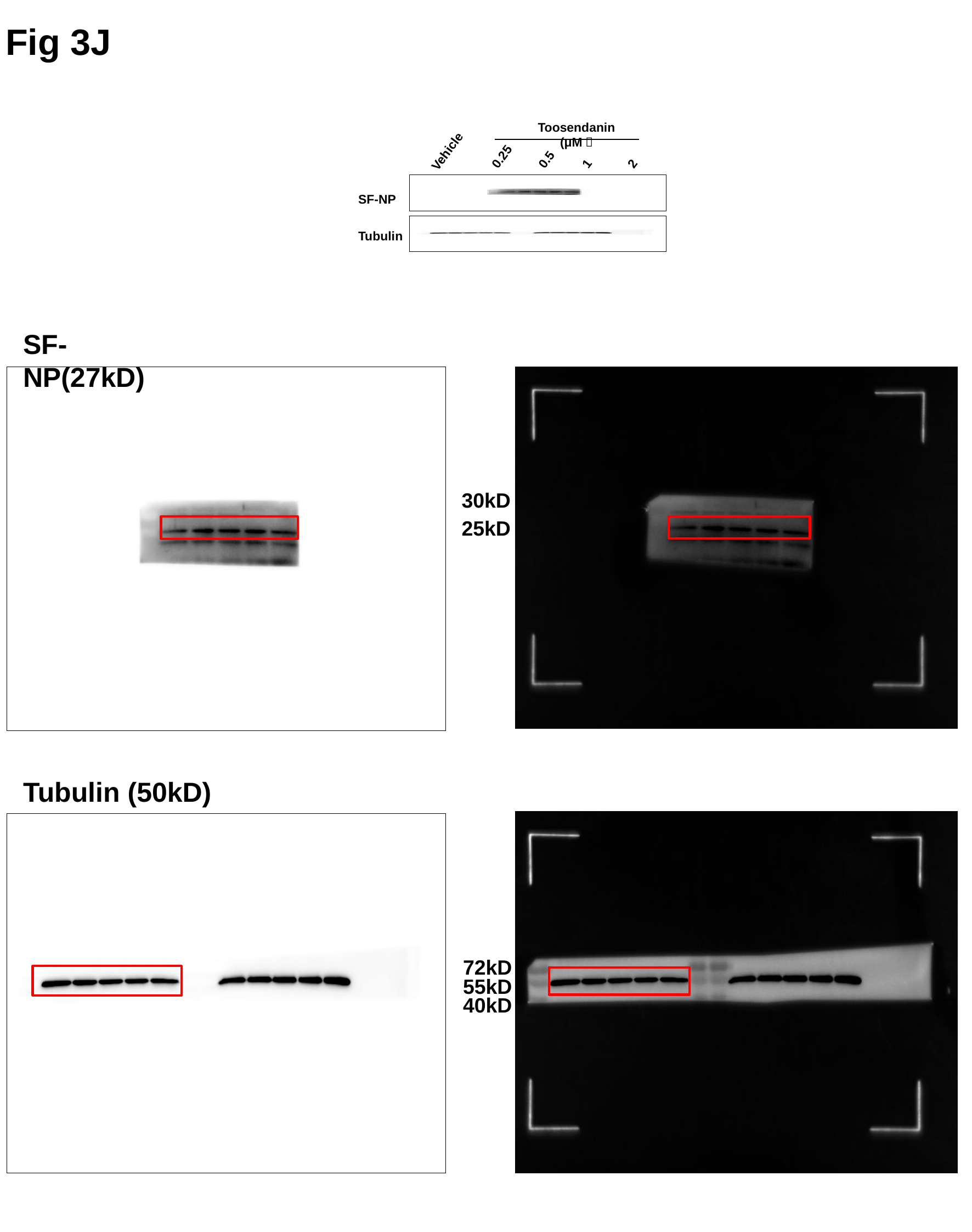

Fig 3J
Toosendanin (μM）
Vehicle
0.25
1
0.5
2
SF-NP
Tubulin
SF-NP(27kD)
30kD
25kD
Tubulin (50kD)
72kD
55kD
40kD

## Slide 4
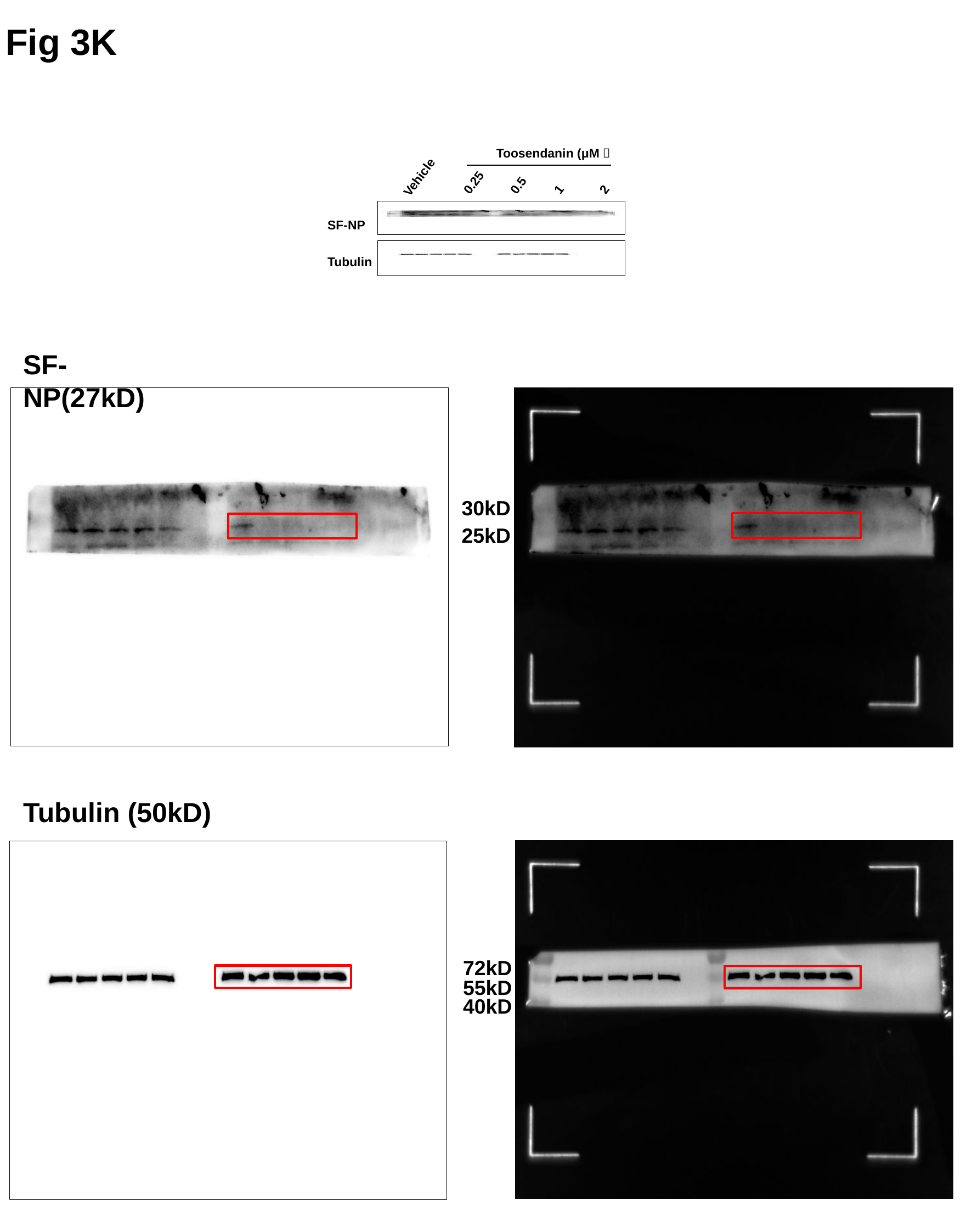

Fig 3K
Toosendanin (μM）
Vehicle
0.25
1
0.5
2
SF-NP
Tubulin
SF-NP(27kD)
30kD
25kD
Tubulin (50kD)
72kD
55kD
40kD

## Slide 5
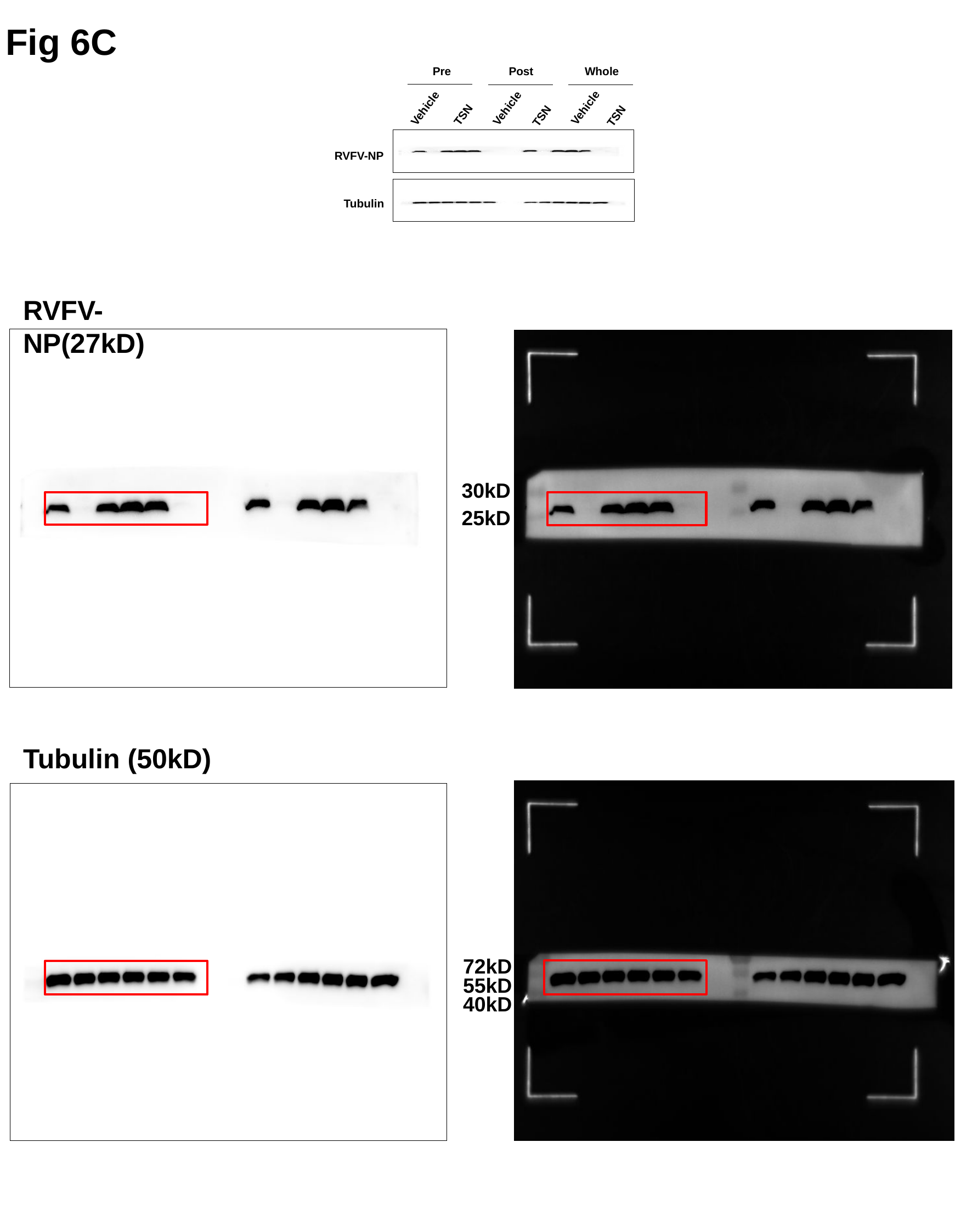

Fig 6C
Pre
Post
Whole
Vehicle
Vehicle
Vehicle
TSN
TSN
TSN
RVFV-NP
Tubulin
RVFV-NP(27kD)
30kD
25kD
Tubulin (50kD)
72kD
55kD
40kD

## Slide 6
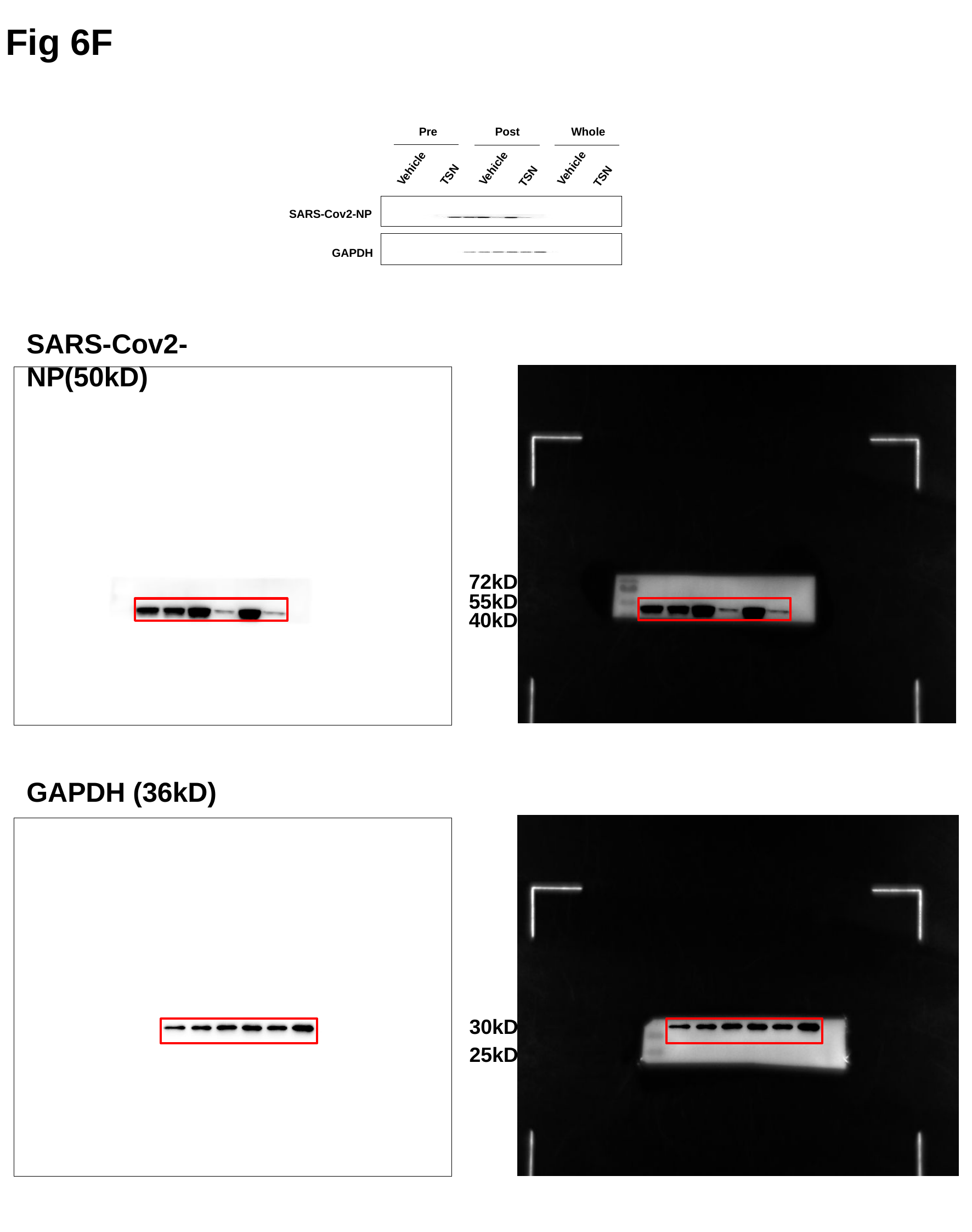

Fig 6F
Pre
Post
Whole
Vehicle
Vehicle
Vehicle
TSN
TSN
TSN
SARS-Cov2-NP(50kD)
SARS-Cov2-NP
GAPDH
72kD
55kD
40kD
GAPDH (36kD)
30kD
25kD

## Slide 7
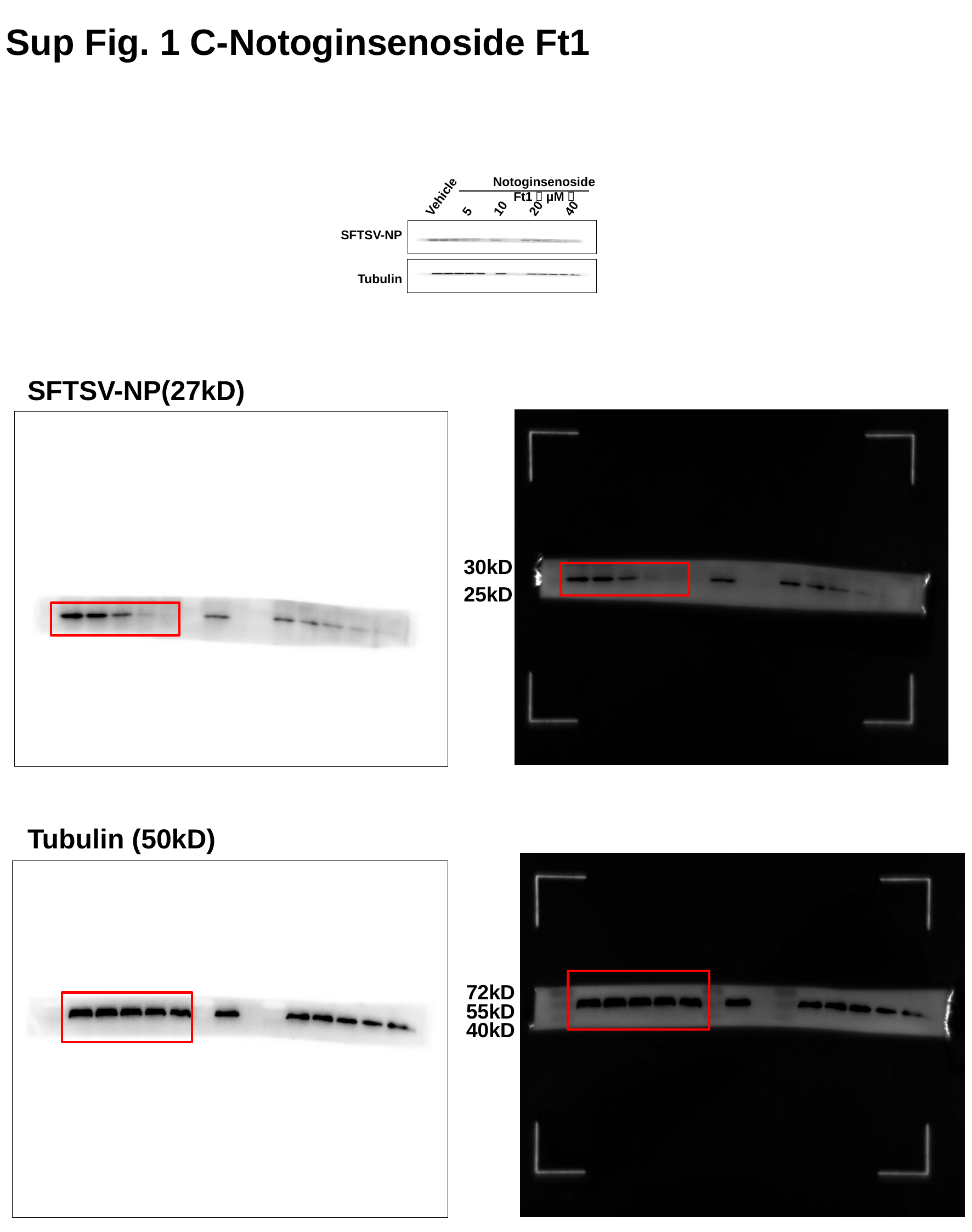

Sup Fig. 1 C-Notoginsenoside Ft1
Notoginsenoside Ft1（μM）
Vehicle
20
10
5
40
SFTSV-NP
SFTSV-NP(27kD)
Tubulin
30kD
25kD
Tubulin (50kD)
72kD
55kD
40kD

## Slide 8
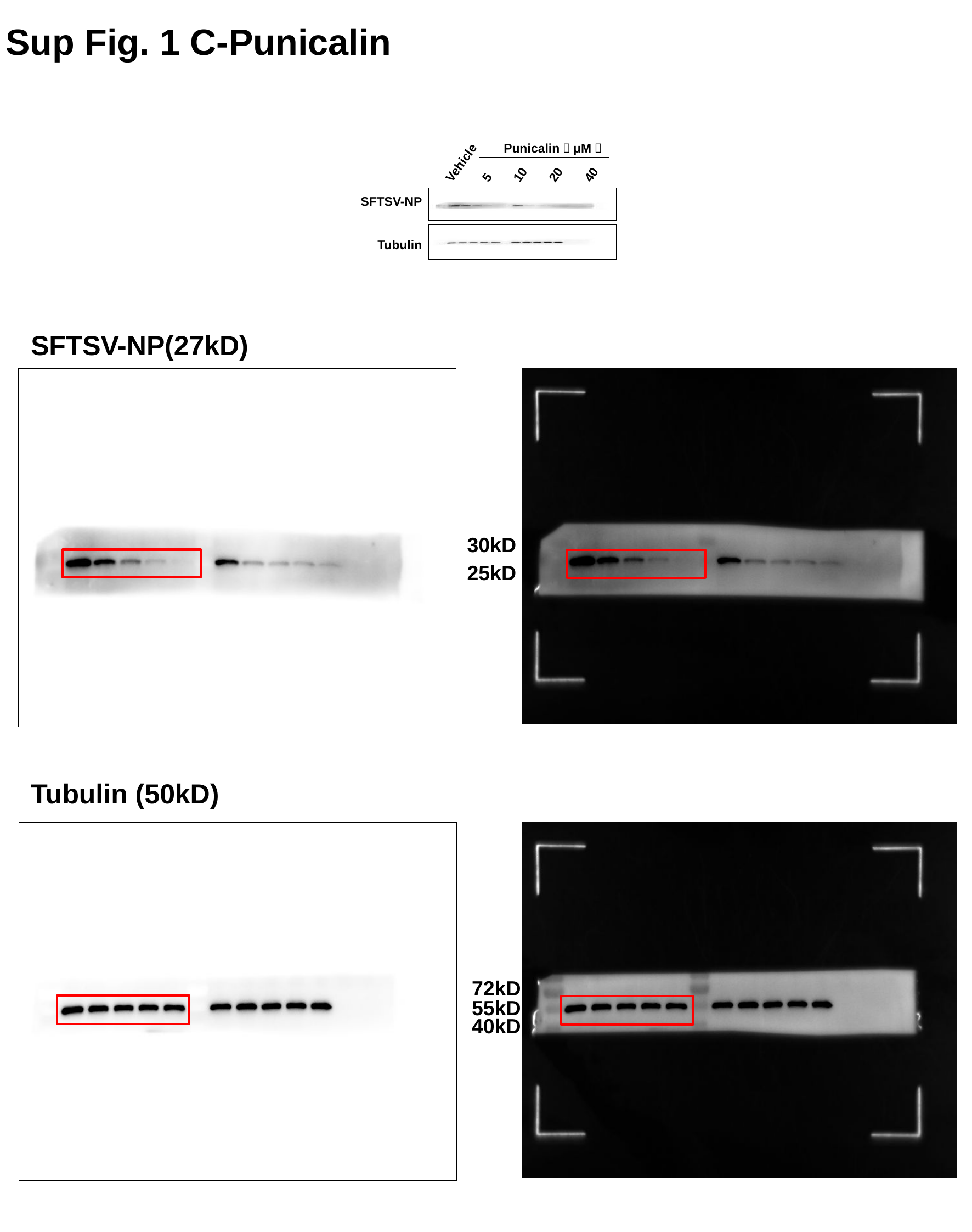

Sup Fig. 1 C-Punicalin
Punicalin（μM）
Vehicle
20
10
5
40
SFTSV-NP
SFTSV-NP(27kD)
Tubulin
30kD
25kD
Tubulin (50kD)
72kD
55kD
40kD

## Slide 9
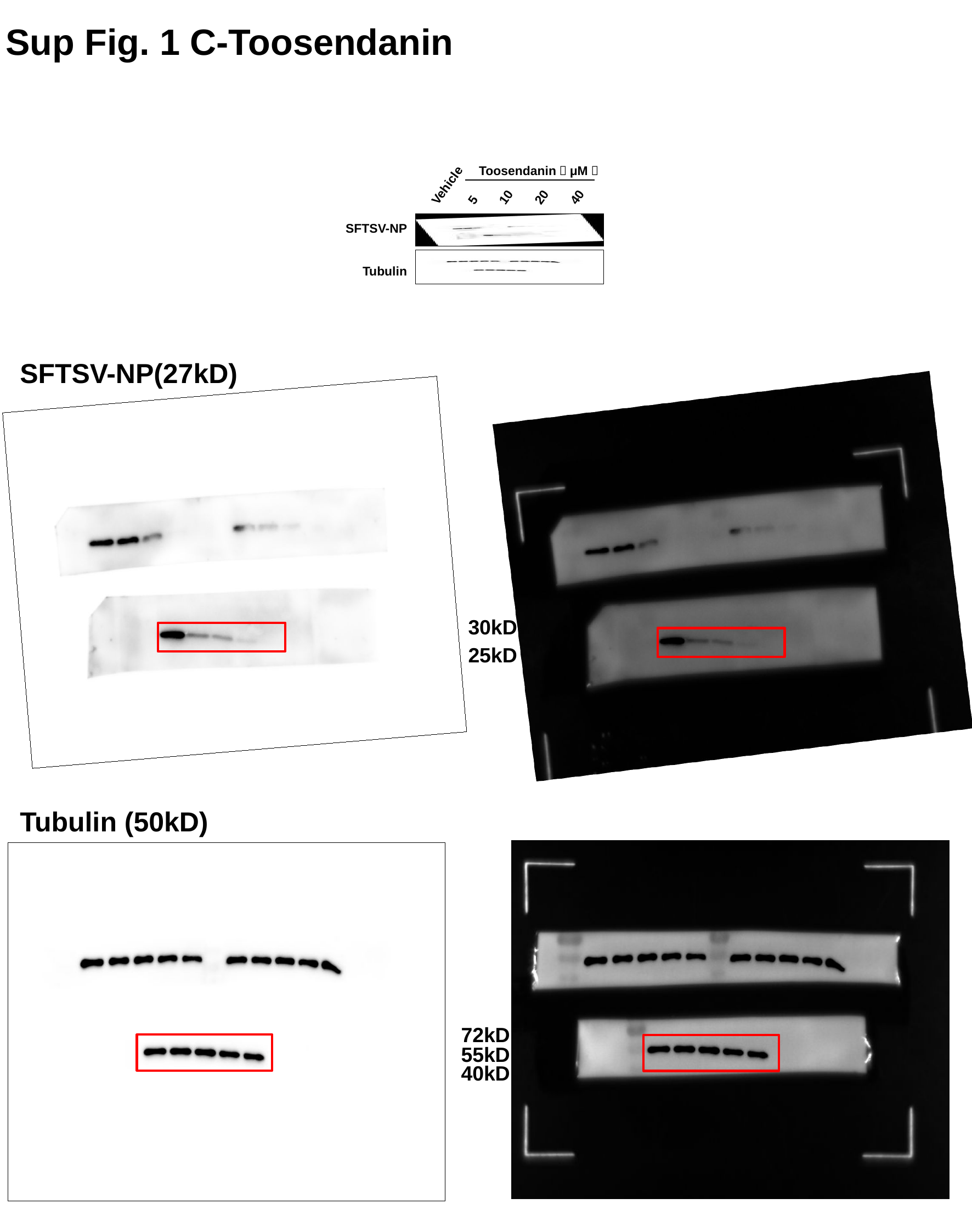

Sup Fig. 1 C-Toosendanin
Toosendanin（μM）
Vehicle
20
10
5
40
SFTSV-NP
SFTSV-NP(27kD)
Tubulin
30kD
25kD
Tubulin (50kD)
72kD
55kD
40kD
